# Open-source benchmarking of IBD segment detection methods for biobank-scale cohorts

**DOI:** 10.1093/gigascience/giac111

**Published:** 2022-12-06

**Authors:** Kecong Tang, Ardalan Naseri, Yuan Wei, Shaojie Zhang, Degui Zhi

**Affiliations:** Department of Computer Science, University of Central Florida, Orlando, FL 32816, USA; School of Biomedical Informatics, The University of Texas Health Science Center at Houston, Houston, TX 77030, USA; Department of Computer Science, University of Central Florida, Orlando, FL 32816, USA; Department of Computer Science, University of Central Florida, Orlando, FL 32816, USA; School of Biomedical Informatics, The University of Texas Health Science Center at Houston, Houston, TX 77030, USA

**Keywords:** identical-by-descent, biobank-scale data, IBD segment detection tools, benchmarking

## Abstract

In the recent biobank era of genetics, the problem of identical-by-descent (IBD) segment detection received renewed interest, as IBD segments in large cohorts offer unprecedented opportunities in the study of population and genealogical history, as well as genetic association of long haplotypes. While a new generation of efficient methods for IBD segment detection becomes available, direct comparison of these methods is difficult: existing benchmarks were often evaluated in different datasets, with some not openly accessible; methods benchmarked were run under suboptimal parameters; and benchmark performance metrics were not defined consistently. Here, we developed a comprehensive and completely open-source evaluation of the power, accuracy, and resource consumption of these IBD segment detection methods using realistic population genetic simulations with various settings. Our results pave the road for fair evaluation of IBD segment detection methods and provide an practical guide for users.

## Introduction

Identical-by-descent (IBD) segments (i.e., DNA segments inherited from a common ancestor) [1, 2] provide direct evidence of genetic relatedness. It plays a key role in population genetic research. Given whole-genome genetic data of a cohort of individuals, IBD segments could be used to detect relations among them [3, 4]. IBD segments are used in association analysis and can also be directly used in IBD mapping to detect signals of disease-causing markers in population samples [5–9]. IBD segments can also be used to estimate missing genotypes from the haplotype or genotype reference panels [10]. IBD segments have also been used to phase genotype data [11, 12]. Direct-to-consumer (DTC) genetic testing companies use IBD segments to offer services of inferred family history [13]. IBD segments may help to identify individuals without direct access to their genetic data in forensic settings [14].

Several IBD segment detection tools have been developed in the past decade. The early generation of tools was designed to detect IBD segments among hundreds to thousands of individuals from a genotype or a haplotype panel [1, 15–17]. In the biobank era, with the need for processing hundreds of thousands or even millions of haplotypes, a new generation of efficient IBD segment detection tools has been designed in the past few years [18–22].

While all these methods claim to be efficient and accurate, a direct and reproducible comparison of these methods is still missing. Each of the methods uses a unique solution to solve the IBD detection problem. As a result, some of the proposed methods may be more suitable for certain settings but may fail in some other cases. For example, one method may not work well in the presence of high genotyping errors. Moreover, the efficiency of some approaches could be more significant in very large data.

Therefore, in this work, we aim to systematically benchmark the new generation of IBD segment detection methods. To facilitate transparency, reproducibility, and convenience, we choose to leverage the latest advanced population genetics simulation tools. For the quality assessment of the IBD call, we define several metrics. The metrics aim to evaluate the accuracy of IBD calls and the detection power of the tools. We define 3 accuracy and 3 power metrics considering coverage and length in both single segments and multiple segments upon 3 sets of data. Moreover, we evaluate the tools regarding the run time and memory consumption. We measure run times and memory usages with the same abundant resources for all tools with increasing sizes of data input.

## Methods

When ground-truth IBD segments in real data are available, real data are always preferred. However, benchmarking using real data is limited to only close relatives and mostly for long IBD segments. With advanced population genetics simulation tools, we can have precise ground-truth IBD segments among the whole simulated population. Coalescent simulators have been used to evaluate the IBD calls previously [18–20]. Here, we also use the coalescent simulation tool msprime [23] to simulate the datasets. To benchmark the power and accuracy of the IBD detection tools, we used simulated data. On the other hand, for the run time and memory usage, we used UK Biobank data [24]. We used the phased haplotype panels to input the IBD segment detection tools for all of our benchmarks.

### Simulated datasets

We used msprime v1.0.1 to generate 3 population datasets: East Asian (EAS), European (EUR), and African (AFR). Additionally, we generated a mixed set of EAS, EUR, and AFR. These datasets contain the chromosome 20 sequences of 4,000 individuals (8,000 haplotypes), based on the out-of-Africa population model [25]. We used HapMap phase II GRCh37 [26] as the recombination map and 1.38 × 10^−8^ as the mutation rate. The true IBD segments are determined as the contiguous segment among the tree sequence generated by msprime, where the haplotype pairs share the same most recent common ancestor (MRCA). We sampled the trees for every 5,000 base pair physical distance, and the true IBD segments were extracted if their genetic lengths were at least 1 centiMorgan (cM). Once the datasets were simulated, we filtered out the sites having multiple allele values and singletons.

Then we generated array density datasets by downsampling the original sequencing datasets. To achieve an even marker density, a common practice in marker design, each sequencing panel was first given a target number of markers, and we used the number 17,197, same as the number of markers in UK Biobank chromosome 20. Then a centiMorgan interval (*I*) was calculated from dividing the total genetic length of the chromosome by the desired number of sites, and this (*I*) indicated the ideal distance between 2 markers. As mentioned, in many cases, there may not be any marker in many continuous (*I*); therefore, a window size (*w*) was considered by attempting to take *w* markers from a *w*-by-*I* range. After that, our program tried to select each marker from its original (*I*) first, then tried to take a marker from the leftover markers in the *w*-by-*I* range if there was not any marker in the original (*I*). During the marker selection, a site with the highest minor allele frequency (MAF) was selected. Acquiring a perfectly even marker density and reaching the desired number of markers are 2 conflicting goals. After multiple runs with different window sizes, we chose 5 as the window size. At the end, we had 15,958 sites in the EAS array data, 15,546 sites in the EUR array data, 15,313 sites in the AFR array data, and 16,013 sites in the mixed array data.

To simulate genotyping errors, we randomly implanted genotyping errors with the rates of 0.1%, 0.2%, 0.3%, and 0.4% per genotype over the variant sites. Although this is a simplification of the realistic error profiles in sequencing data (e.g., false positively called nonvariant sites were not included, nucleotide-specific and region-specific error profiles were not modeled), we did include a singleton filter, which we assumed would remove most of false positively called nonvariant sites. As we observed that several methods did not tolerate errors well in sequencing data, we also generated additional datasets with 0.0125%, 0.025%, and 0.05% error rates for sequencing data. Errors were added incrementally from lower to higher error-rated data to aid interpretability.

Furthermore, we also simulated phasing errors using a standard haplotype phasing method to investigate the effect of phasing errors. For the phasing error simulation, we simulated haplotypes of European ancestry using stdpopsim. The HapMap genetic map (GRCh37) was used to simulate haplotypes of chromosome 20. We used the population model OutOfAfrica_2T12 defined in stdpopsim and generated these 2,000 Europeans. Every 2 consecutive haplotypes were merged into 1 genotype. Then, 0.1% genotyping errors were added to the panel. At the end, SHAPEIT4 [27] was used to rephase the genotype data without using any reference. At the end, we had an average 0.17% switching error calculated by VCFtools [28].

### UK Biobank dataset

While real datasets are often not suited for evaluating power and accuracy, they can be used to estimate the efficiency of the tools regarding the run time and memory usage. For the run time and memory usage benchmark, we used chromosome 1 of the UK Biobank. This dataset contains 487,409 individuals and 53,260 markers with a total file size of  100 GB. We created subsets of the input by reducing the number of individuals from the full size to 250, 125, 62.5, 31.3, and 15.6 thousand to evaluate the scaling up of the methods. Due to potential license conflicts, we did not run TPBWT on UK Biobank data. A set of simulated panels was created for the run time and memory tests. This dataset had a similar number of individuals (500,000) and a similar number of sites (51,190), and it was tested to have a similar number of detected IBD segments from other tools compared to the original UK Biobank chromosome 1.

### Evaluation metrics

**Figure 1:**
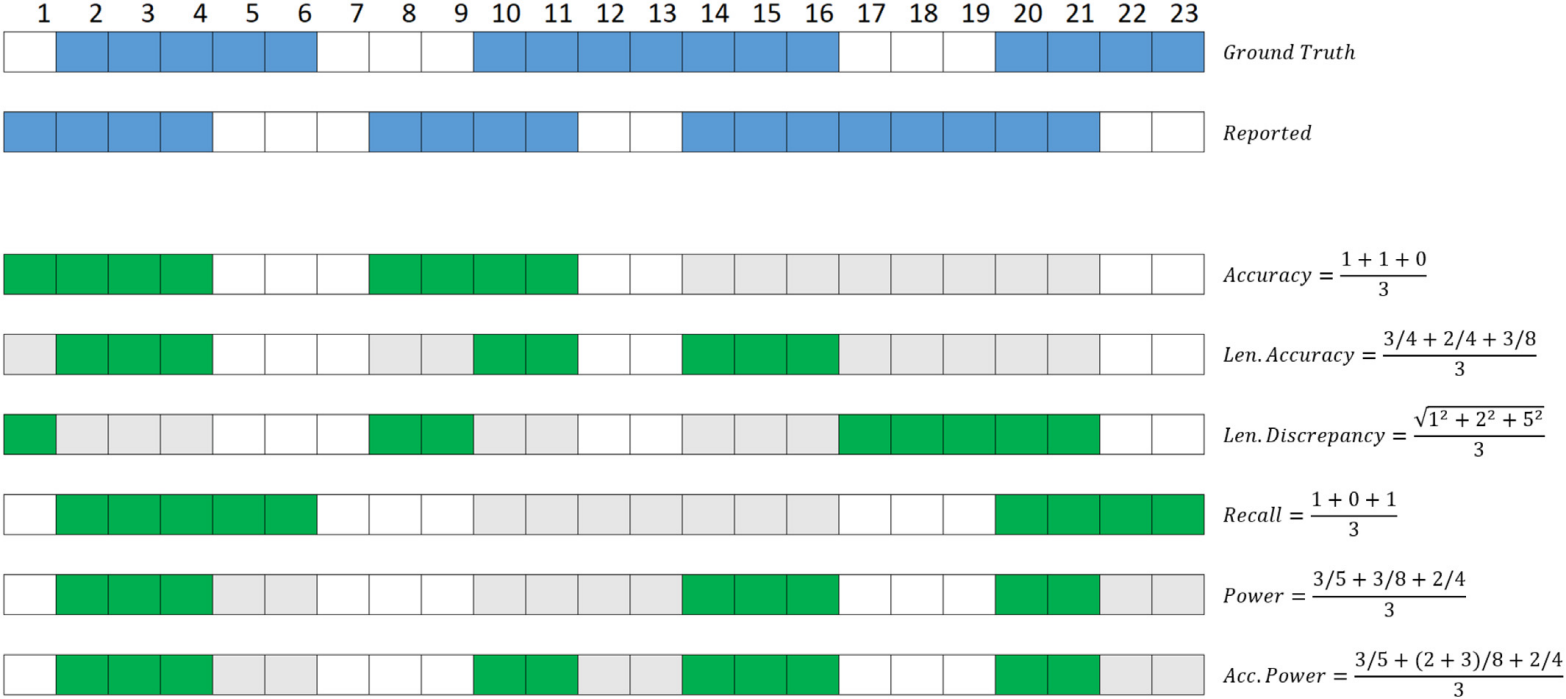
Demonstration of accuracy and power measures. The blue segments represent existing ground truth and reported IBD segments, the green segments donate the segments that are to be considered in calculations, and the gray segments are not to be considered during calculations. Accuracy is counted if a reported IBD segment could be covered by any 1 ground-truth IBD segment by 50%; as shown, the last reported IBD segment was not considered. Length accuracy is calculated with a best ground-truth IBD segment that could cover the reported IBD with maximum length. Length discrepancy is calculated by considering the length difference with a best-matching ground-truth IBD segment. Recall is courted if a ground-truth IBD segment could be covered by 50%; as demonstrated, the second ground-truth IBD segment was not counted since none of those reported IBD segments could cover 50% of this ground-truth IBD segment. Power is a similar measure to length accuracy but measures the segments of the ground-truth IBD segment that are covered by a best-reported IBD segment. Accumulative power considers multiple reported IBD segments to cover a ground-truth IBD segment.

One of the limitations of existing benchmarks is that they use nonunified definitions of metrics. Here we aim to provide a set of well-defined and standardized metrics. For IBD segment detection performance evaluation, similar to other information retrieval problems [29, 30], we consider 2 aspects that should be examined together. First is the precision of the reported IBD segments. Second is the ability of the method to recover the true IBD segments. The reported IBD segments cannot simply be evaluated using precision and recall since the reported segments might be partially true or a ground-truth IBD segment can be partially reported. Moreover, a ground-truth IBD segment can be reported as multiple segments. Fig. 1 shows the evaluation metrics to assess the quality of IBD segments. In the following subsections, all the metrics are described in detail.

Each evaluation metric compares the sets of ground-truth IBD segments and reported IBD segments. For the comparison, one can compare the variant sites, physical locations, or genetic locations. By comparing the genetic locations (in cM), the quality of the IBD segment call can be assessed in a wide range of applications, such as genealogical search and association analysis. Considering variant sites may be more suitable for applications such as IBD mapping. However, genetic location would still largely be consistent with the variant site comparison. In this work, we calculated the evaluation measures using the genetic locations of ground-truth and reported IBD segments. To observe the performance of different IBD segment lengths, ground-truth IBD segments and reported IBD segments were collected as full sets and binned sets as [2,3), [3,4), [4,5), [5,6), and [7,∞) cM bins. Then the accuracy calculations were carried out by taking each bin set as an evaluation target with the full set of ground truth as the reference set. This method overcame a major problem when a reported IBD segment’s length was very close to ground-truth IBD segment length but was binned into a different bin; for example, a 2.9 cM reported IBD segment was binned into the [2,3) cM bin. But when the ground truth was 3.0 cM and divided into the [3,4) cM bin, if we had only considered the [2,3) cM ground-truth set as the reference, this almost perfectly reported IBD segment would not have been counted. The same approach was applied to power calculations, and we considered a full set of reported IBD results as the reference set to compute with each ground-truth bin.

The reason we use these multiple of metrics is that there is no single metric that can describe all aspects of IBD segment detection performance. Indeed, depending on the downstream analysis tasks, different aspects of IBD segment detection are weighted differently. For example, genealogy inference algorithms use the total IBD segment length. Some use the total number of IBD segments, and some use the lengths of individual IBD segments. For IBD mapping, the actual set of markers that are called is more important.

#### Evaluation of reported segments

In order to evaluate the quality of reported segments, we define 3 measures: *accuracy, length accuracy*, and *length discrepancy*. The *accuracy* is calculated as the number of covered IBD segments divided by the total number of reported IBD segments. A reported IBD segment is considered covered if at least 50% of its length is covered by a ground-truth IBD segment. *Length accuracy* is a more fine-grained measure for reported IBD segments, and the measure accounts for the portions of falsely reported segments along a true IBD segment. To calculate the *length accuracy*, we first find the best-matching ground-truth IBD segment for each reported IBD segment (i.e., the one that covers the reported IBD segment with the longest overlap). Next, the percentage of the reported IBD segment covered is calculated. Finally, an average of these percentages across all reported IBD segments denotes the *length accuracy*. These accuracy measures can reflect the false-positive rate simply by 1 − accuracy. *Length discrepancy* captures the length difference between the reported IBD segment and its best-matching true IBD segment. Over all segments, the root-mean-square deviation is calculated as the *length discrepancy*. For this measure, the smaller the *length discrepancy*, the better the quality of the reported IBD segment. This measure focuses on the length of matching between the reported and the ground-truth IBD segments.

#### Detection power

We define 3 measures to evaluate the ability of the tools in detecting ground-truth IBD segments. The first measure, *recall*, denotes the proportion of the number of true IBD segments that have been reported. Here, we assume a ground-truth IBD segment has been detected if a reported segment covers at least 50% of the ground-truth segment. The second measure, *power*, denotes the average proportion of true IBD segments that are covered by its best-matching reported segment. For each ground-truth IBD segment, its best-matching reported IBD segment is the one that has the longest overlap. The third measure, *accumulative power*, is similar to the second measure but here we consider multiple reported IBD segments. All reported IBD segments that overlap with a ground-truth IBD segment are being considered.

### IBD coverage distribution

The whole chromosome IBD segment coverage was calculated by counting how many times each site was covered by different tools, and we focused on how close each tool matched to the ground truth. This visualization method could also be used to observe how each tool handled different regions. On the other hand, it also indicates special regions upon the chromosome. For the ground truth, the threshold cutoff was set to 2 cM. As a result, some tools might have had slightly higher values, especially if they overestimated the IBD boundaries.

### Relatedness inference

To evaluate the application of relatedness inference, we simply used the calculated total length of reported IBD segments between each individual pair. The threshold values  [31] were calculated by the total IBD sharing to assign the degrees of relatedness up to the fourth degree. We did not adjust the threshold values to account for the reduction of power that resulted from genotyping errors. The genotyping error rate was set to 0.1%. Datasets both with and without phasing error were used for this experiment.

### Population and influence of marker selection

We first simulated 3 separated population panels and a mixed panel that contained all 3 populations to observe the performance of each tool upon different population panels. In addition, considering each population has their own set of preferred markers, using a marker set designed for a different population may yield poor results. Therefore, we conducted another set of experiments to observe the influence of marker selection. First, 3 subpanels were extracted from the mixed panel by population. Then, the ideal set of markers for each subpanel was determined using the downsampling method in the simulated datasets section. After that, array datasets with all combinations between 3 populations and 3 marker sets were created with a 0.1% genotyping error rate. At the end, we input each tool with all these array datasets to evaluate the performance.

### Run time and memory usage

The idea of run time and memory benchmarking was to give practically sufficient resources and use increasing sizes of sample to input each tool, then observe each tool’s performance. Therefore, for each experiment, we allocated a maximum of 500 GB memory and 60 hyperthreading CPU cores with a 3.00-GHz clock speed in our computation node. We collected 3 reported pieces of information from the “sacct” command of Slurm Workload Manager version 20.02.2. The wall clock time corresponded to “Elapsed,” the CPU time was reflected by “TotalCPU,” and peak memory consumption was measured by “MaxRSS.”

The wall clock time tells the user how long it takes to get the job done in the real world. It gives the most direct impression of a software, regardless of time-sensitive cases, but most people still like to have the job done quickly. The CPU time, a traditional measure of time complexity in algorithms, measures how much total workload the task really is. With advanced multicore computer hardware, this number could be much different from the wall clock time. With the same sufficient resources, a small job and a massive job could be done in the same amount of real-world time, because the big job may take dozens of CPU cores but the small job may just use a fraction of a single core. Memory consumption is used to reflect the space complexity, since hard drive storage is no longer a major limit, and memory size is the major scale-limiting factor. If a task requires too much memory, it could be practically impossible. On the other hand, if an algorithm is efficient enough to solve a big problem with a small size of memory, it means tasks could be done in a practical and low-budget approach. Most important, this means that with the same amount of memory, this method can solve the problem on a larger scale. The degree of parallelism describes how well a tool could fully utilize modern computer resources. The degree of parallelism is defined as the ratio of CPU time and wall clock time. If the number is very close to 1, it means the tool does not take advantage of multicores. On the other hand, the tool is well parallel programmed if the number is much higher than 1 and even closer to the number of cores allocated. This number could also be divided by the number of cores allocated to represent CPU utilization. This measure indicates the decency of modern software engineering, and it potentially tells how fast the tool could solve the problem in the real world.

### Selected tools

We chose the latest generation of IBD segment detection tools that have been designed for biobank data scale in the past few years: FastSMC, hap-IBD, iLash, RaPID, and TPBWT. Since each tool has a wide range of parameter combinations, it is practically difficult to acquire the optimal parameter combination for each tool in each case. We spent the same amount of effort on each tool and tried to find proper parameters for different cases. We used default parameters if we could not find better parameters. All the parameters for different tools have been included in Supplementary Table S1.

FastSMC applies hashing methods to identify IBD segments, then uses a coalescent-based hidden Markov model (HMM) to verify the segments. FastSMC uses an ascertained sequentially Markovian coalescent (ASMC) to estimate the posterior of the time to most recent common ancestor (TMRCA) for pairs of individuals. It has been implemented in C++ and with optional Python bindings. The input Variant Call Format (VCF) file needs to be converted to an Oxford phased haplotype file, and the genetic map needs to be processed to have exact sites in the original VCF file. The software requires so-called Decoding quantities files. The files for some populations have been provided in the package.

hap-IBD uses a seed-and-extend technique with positional Burrows–Wheeler transform (PBWT) [32]. Due to the efficient extraction of haplotype matches using the PBWT, it is very time and memory efficient. hap-IBD has been implemented in Java, which provides convenient cross-platform execution. It does not require any data conversion of the VCF file. The required genetic map file format is PLINK, while the sites in the map file do not have to be the exact sites in the VCF file. hap-IBD is well engineered to execute in parallel.

iLash applies sliding, minhashing, locality sensitive hashing, and pairwise extension to report IBD segments. iLash was programmed in C++, and it also supports parallel execution. The input VCF and genetic map files need to be converted to PLINK PED and MAP formats.

RaPID leverages multiple random projections to perform approximate haplotype matching, then applies PBWT and merging methods for IBD detection. RaPID has been implemented in C++. The input file should be in compressed VCF format, and the genetic map needs to be preprocessed to match exact sites in the VCF file. The command line parameters can be calculated with the provided Python script by considering genotyping error rate, marker density, and the minimum length of IBD segments. The author also has recommended a set of parameters for general cases.

TPBWT extends PBWT by adding a new dimension to the PBWT that allows mismatches in detected IBD segments. The added dimension simply masks out potential errors in the haplotypes and extends IBD segments even if there is a mismatch between the haplotypes. The argument template, a 2-dimensional Python list, defines the configurations that can tolerate genotyping and/or phasing errors. Similar to RaPID, TPBWT also requires a genetic map to be formatted to match exact sites in the VCF input file. TPBWT has been implemented in Python.

## Results

### Overall evaluation of IBD segment detection tools

**Figure 2:**
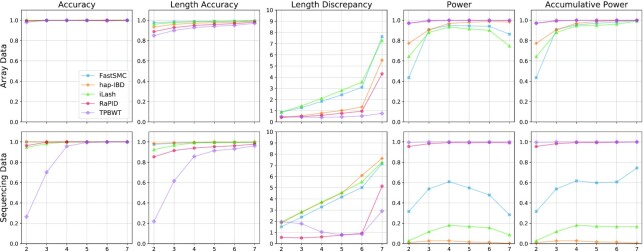
Benchmarking results of different IBD segment detection tools in the EUR array and sequencing data with a genotyping error rate of 0.1% stratified by segment lengths. Some tools have reduced detection power as IBD segment cutoff increases. It is likely caused by fractured IBD segments since this measure only considers 1 reported IBD segment. Accumulative power considers all reported overlap IBD segments, and all corresponding values are monotonically increasing. The length discrepancy is measured by cM; other measures are based on percentage.

Fig. 2 shows the values for 5 different metrics in both sequencing and array data: accuracy, length accuracy, length discrepancy, power, and accumulative power. These metrics can be applied to assess the quality of IBD calls for a variety of applications such as IBD mapping and genealogical inference. The other 2 metrics, recall and length discrepancy metrics, are especially useful for applications that summarize the IBD counts and length (e.g., investigation of population history, pedigree inference).

The genotyping error was set to 0.1%, which is the expected [33–35] error rate in available data. As shown in Fig. 2, FastSMC and iLash both had high accuracy in 2 cM segments and overall lower power. hap-IBD’s accuracy was slightly lower for 2 cM segments, but it had significantly higher detection power. RaPID had high power and competitive accuracy on longer segments. Both RaPID and TPBWT had relatively low length discrepancy in both sequencing and array data and higher detection power in sequencing data. The accuracy of TPBWT in sequencing data was lower, especially for shorter segments. For long segments (≥10 cM), all selected tools had high power and accuracy on inputs without genotyping error. When dealing with genotyping errors, reported segments tended to break into pieces, resulting in low power. Overall, TPBWT had the highest detection power on sequencing data with genotyping error followed by RaPID (see Supplementary Figs. S1 and S2).

### Regional coverage of IBD segments

**Figure 3:**
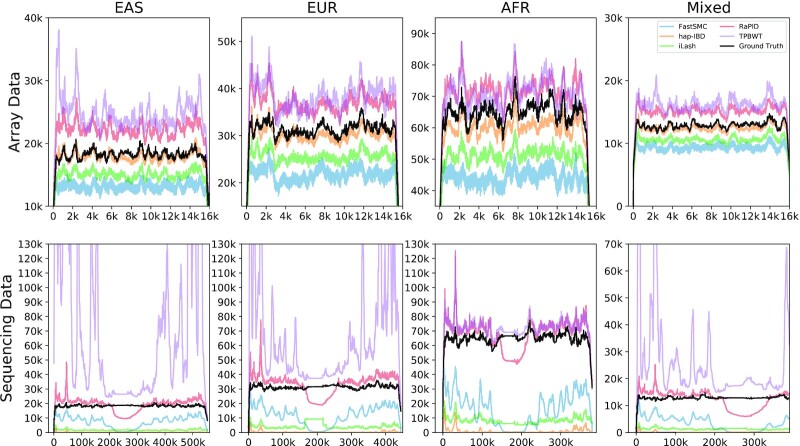
Visualization of IBD segment coverage (over 2 cM) in array and sequencing data with a genotyping error rate of 0.1% on 3 populations. The x-axes are the marker location indexes. The y-axes are number of IBD segments that covered each marker. The ground truths were displayed (black) for reference purposes.

Fig. 3 shows IBD segment coverage results with a 0.1% genotyping error rate. The results of IBD segment coverage without genotyping error are also included in Supplementary Fig. S3. Overall, while the ground-truth IBD coverage was roughly even across the chromosome, all methods produced IBD calls with variability in coverage. For array data, TPBWT and RaPID had overcalling while iLash and FastSMC had undercalling. hap-IBD had the best calibration in terms of overall coverage. For sequencing data, all methods had much greater variability in IBD coverage. IBD coverage of RaPID was the closest to the ground truth in sequencing data.

Based on our results, we inferred that most of the tools were well configured to handle array data. hap-IBD and iLASH had very low detection power for sequencing data with errors. We conjecture that the default parameters for these tools do not offer competitive results for sequencing data. We noticed that TPBWT had much higher false-positive rates in EAS and EUR than in AFR; this further tells that either new sets of sequencing-specific parameters are needed or a preprocessing to thin the panel is required.

### Effect of genotyping errors

**Figure 4:**
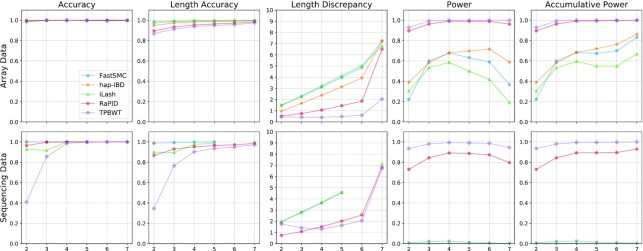
Benchmarking results of different IBD segment detection tools in the EUR array and sequencing data with a genotyping error rate of 0.4% stratified by segment lengths. The length discrepancy is measured by cM; other measures are based on percentage. Some lines may be discontinued or dipped due to low power.

Although most current human genetic panels have a very low genotyping error rate, some historic data and some nonhuman data may still have significantly higher genotyping error rates. Increasing genotyping errors will likely decrease the detection power. Fig. 4 shows the results of different tools in both array and sequencing data using a relatively high genotyping error rate (0.4%). The EUR had 0%, 0.2%, and 0.3% genotyping error rates. The results for other 2 populations and the mixed population are shown in Supplementary Figs. S4 to S21. Overall, TPBWT and RaPID seem to be more robust against high genotyping errors while TBPWT’s detection power remains higher with slightly lower accuracy compared to RaPID in sequencing data. The detection power of hap-IBD, FastSMC, and iLASH is impacted noticeably by higher genotyping errors. The results with lower genotyping errors in sequencing data have been included in the Supplementary Figs. S22 to S25. The recall results are included in the Supplementary Figs. S26 to S28. The recall values are overall consistent with the power values. Length discrepancies of RaPID and TPBWT are lower, especially for longer segments in the presence of genotyping errors. This reflects that other methods tend to break long IBD segments into pieces, thus increasing length discrepancy significantly.

### Relatedness inference

**Figure 5:**
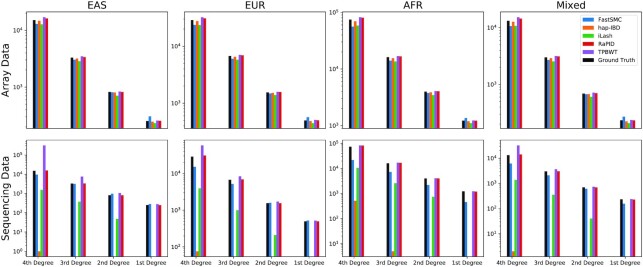
Relatedness detection results in array and sequencing data with a genotyping error rate of 0.1% on 4 population panels. The y-axes are the number of individual pairs found in each category. The ground truths are displayed (black bars) for reference purposes.

As shown in Fig. 5, the simulated data had a realistic distribution of close relatives, where the number of relative pairs increased exponentially with the degree of relatedness. For relatedness inference, we found that, on array data, all methods achieved a decent calls. This is understandable as calling close relatives mainly relies on the accumulated power for long segments, and all methods are very capable of doing that. hap-IBD, TPBWT, and RaPID had the closest reported pairs to ground truth. hap-IBD had slightly less reported pairs, while TPBWT and RaPID tended to report a few more pairs. Both FastSMC and iLash tended to report a fewer number of pairs in most of the cases, while FastSMC tended to report more pairs in the first degree. On sequencing data, not all methods are well calibrated. The power of hap-IBD was most severely reduced, followed by iLash. FastSMC had less impact by genotyping error and had a closer number of reported pairs. Both TPBWT and RaPID had decent power, although TPBWT had a tendency of overcalling in fourth- and third-degree relatives in EAS and EUR. All tools had similar good performance on data without genotyping error (see Supplementary Fig. S29). Although phasing errors may break long IBD segments into smaller pieces, Supplementary Fig. S30 shows that phasing errors had little impact on the relatedness detection.

### Performance in different populations and marker sets

As shown in Figs.  3 and 5, although different populations had a different marker density and average IBD coverage, the performance of all methods was relatively stable. A main outlier was that TPBWT had a much higher false-positive rate in EAS and EUR than in AFR. This may have been due to a lower level of linkage disequilibrium in AFR. Based on our observations, there was no significant difference between the mixed population panel and the separated panels.

The results on the marker set selection (Supplementary Figs. S31 and S32) show that using a set of markers designed for a different population had noticeably lower accuracies, especially in terms of length accuracy and length discrepancy at shorter target lengths. Powers, on the other hand, were slightly increased. This is because the set of markers designed for a different population had a lower MAF than the set of markers designed for the population of interest, thus increasing the chance of random allele matches. However, the overall effects of suboptimal marker set selection were minor in our experiments.

### Robustness against phasing errors

The benchmarking results for all tools in the presence of phasing error are included in Supplementary Tables S2 to S7. The reduction of power was from 4% to 7% for different tools for short segments (2–3 cM). The reduction in power for very long segments (≥15 cM) ranged from 5% to 20% without any special treatment of phasing errors, with a strict threshold cutoff length. However, including shorter segments resulted in a 4% to 10% difference in detection power for very long segments. TPBWT with phasing error tolerance had only a 5% reduction in detection power for very long segments (≥15 cM) with a minor reduction in length accuracy. TPBWT with phasing error tolerance mode was also able to increase its detection power for shorter segments by almost 2% for 2 cM segments, while the reduction in accuracy/length accuracy was more noticeable. In general, the reduction of detection power after the data were rephased was not very significant for short IBD segments. This is due to the high accuracy of the current phasing algorithms with the availability of large biobank scale cohorts. The phased data may contain some long-range switch errors or blips, but they will not contribute to a noticeable reduction in the detection power except for strict and very long IBD cutoff thresholds. The possible reduction of power can also be alleviated for some downstream analysis, especially if the total shared IBDs between 2 individuals is of interest [36]. However, the impact of phasing errors could be more consequential if the number of segments is being considered.

### Run time and memory useage

**Figure 6:**
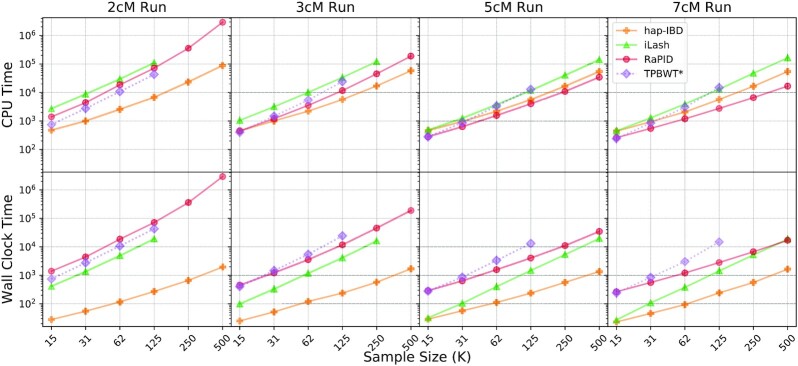
Run time results based on UK Biobank chromosome 1. CPU time and wall clock time are displayed in the first and second rows. Each column of the subfigures represents runs with a different IBD segment cutoff (2, 3, 5, and 7 cM). The x-axes in each subfigure are the input size in a thousand individuals. The TPBWT’s results were based on the simulated panels.

The run time and memory consumption experiments were carried out on our 500-GB memory server as mentioned; iLash and TPBWT could not finish all the experiments with 500 GB memory. Due to potential license conflicts, we did not run TPBWT on UK Biobank data, and the time and memory consumption results for TPBWT were based on the simulated dataset, as mentioned in the Methods section. Since FastSMC requires the most recent updated operating system and libraries, this could be a problem since most of the servers do not use the latest versions. We were able to make FastSMC executable on a 32-GB memory PC for accuracy and power assessment through some efforts. Thus, we were not able to run FastSMC for large panels. We ran FastSMC on panels with smaller sample sizes (from 1,000 to 31,000) on the 32-GB PC. By measuring run times and memory consumptions of these smaller inputs, with an extrapolation using second-order polynomial regressions, we estimated that the FastSMC run time would be 126 days and memory consumption would be 6.5 TB for the whole chromosome 1 of UK Biobank data. Therefore, we did not include FastSMC in Fig. 6. More details can be found in the Supplementary Table S8.

hap-IBD had the shortest wall clock time due to the efficient underlying method and efficient parallelization. It took around 0.5 hours for hap-IBD to complete the IBD segment calls for UK Biobank chromosome 1 with a 2 cM IBD cutoff. iLash won second place on wall clock time, but as mentioned, it could not finish some large cases. Both hap-IBD and iLash took a good advantage of parallel execution. hap-IBD was able to utilize about 75% of the CPU resource as the degree of parallelization was 45.27 out of 60. iLash reached an average parallelization of 9.23 out of 60. CPU time of RaPID was the shortest among all the tools for longer segments (e.g., 5 cM). hap-IBD had the lowest CPU time for short IBD segments (2 cM). The CPU time for TPBWT was also noticeably short for smaller panels (e.g., 15 K), but the time increased fast with the increasing sample size. iLash and TPBWT did not complete the task with the largest panels within the provided memory.

As shown in Tables  1 and  2, RaPID requires the least amount of memory. The memory consumption of RaPID was limited to less than 8 GB, while some other tools required hundreds of gigabytes of memory. hap-IBD also had an efficient memory usage, with the maximum memory usage of about 112 GB memory. The memory consumption results for 3 cM and 5 cM length cutoffs can be found in Supplementary Tables S12 and S13. We observed that the memory consumption of hap-IBD and iLash for the simulated dataset doubled compared to the original UK Biobank data, so we predict that TPBWT could finish the original 250,000 individual subset of UK Biobank chromosome 1. The run time and memory consumption results for this simulated set can be found in Supplementary Tables S8 to S10.

**Table 1.** Memory consumptions based on UK Biobank chromosome 1 with 2 cM length cutoffs

	Sample size (K)					
	15	31	62	125	250	500
hap-IBD	1.76 MB	7.88 GB	8.79 GB	22.81 GB	59.03 GB	110.15 GB
iLash	7.85 GB	20.05 GB	58.73 GB	191.46 GB	na	na
RaPID	728.72 MB	1.03 GB	1.54 GB	2.47 GB	4.14 GB	7.25 GB
TPBWT*	7.70 GB	30.59 GB	122.16 GB	488.40 GB	na	na

* The TPBWT’s results were based on the simulated panels.

**Table 2.** Memory consumptions based on UK Biobank chromosome 1 with 7 cM length cutoffs

	Sample size (K)					
	15	31	62	125	250	500
hap-IBD	1.20 MB	9.93 GB	23.24 GB	15.62 GB	31.78 GB	110.03 GB
iLash	1.77 MB	5.39 GB	15.25 GB	30.59	77.48 GB	178.83 GB
RaPID	347.13 MB	718.97 MB	1.22 GB	2.21 GB	3.90 GB	6.33 GB
TPBWT*	7.70 GB	30.60 GB	122.16 GB	488.40 GB	na	na

* The TPBWT’s results were based on the simulated panels.

## Conclusion and discussion

We conducted the first open-source, fully transparent comprehensive benchmarking of modern efficient IBD segment detection methods. Based on our benchmarking, most tools yielded decent results in most settings, even in the presence of phasing errors. However, genotyping error rates and marker densities affected the performance of IBD detection methods in different degrees.

For short IBD segments (e.g., 2 cM), FastSMC and iLash had high accuracies but low power in both sequencing and array density. Both hap-IBD and RaPID had high power and relatively lower accuracy in array data, and TPBWT achieved the highest detection power in sequencing data followed by RaPID, especially in the presence of high genotyping errors. For longer IBD segments (≥5 cM), all 5 tools had comparable accuracy, while hap-IBD, RaPID, and TPBWT had high power in array data. iLASH and FastSMC may have reported IBD segments as multiple short segments, especially for longer segments (e.g., 5 cM). The accuracy of most tools for long segments (≥10 cM) was high, but the detection power of some tools was affected significantly with genotyping errors. TPBWT and RaPID had the highest detection power for sequencing data in the presence of genotyping errors, with TPBWT being the most robust against high genotyping error rates.

In general, the minimum length threshold impacts the accuracy, as reflected in our results. We acknowledge that the methods are more likely to report an IBS (Identity by State) as an IBD segment for shorter segments. The minimum threshold length can vary for different applications; for example, for genealogical search, long IBD segments (e.g., 5 or 7 cM) may be used, while for IBD mapping, shorter IBD segments (e.g., 2 or 3 cM) might be used. Our aim was to benchmark the performance of different tools for different IBD segment lengths that provide the researchers with the expected accuracy for their applications.

FastSMC, hap-IBD, and iLASH had high accuracy in sequencing data, especially for short segments (e.g., 2 cM), but their detection power was low. The accuracy of all tools was relatively high for long IBD segments (e.g., 5 cM). Genotyping errors in the sequencing data may affect the results of hap-IBD, FastSMC, and iLASH significantly. A possible solution to run these tools could be downsampling the data using MAF.

Most IBD segment detection tools can handle VCF files. hap-IBD does not require any data preparation while RaPID and TPBWT require minor data preparation. FastSMC and iLash require some data conversion if the available data are in VCF format. FastSMC also requires additional files that are population specific.

Regarding the resource requirements, only RaPID and hap-IBD were able to be used with limited resources, while others required significantly more memory for large panels. RaPID was the most memory-efficient tool, taking 12 times less memory than hap-IBD. On the other hand, hap-IBD had the highest resource utilization, followed by iLash. hap-IBD was also the fastest method for short IBD cutoff lengths in our experiments, while RaPID had less CPU time for a longer IBD cutoff (e.g., 5 cM).

Our study has several limitations. First, as mentioned, the parameter combination for each tool may not be optimal for each case. Finding out the best combinations for each tool is beyond the scope of this work. However, we open-sourced our entire benchmarking protocol and thus we hope others can contribute and release their optimized parameters on the same datasets. Second, although simulated datasets are somehow reflected in the real world, there is still a possibility that the real data could have some unique characteristics. In general, it is not straightforward to extract ground-truth IBD segments from real data. The availability of several pedigree data may provide the opportunity to extract ground truth from real data. The same evaluation metrics, however, then can be used to evaluate the performance of IBD segment detection tools. Moreover, we simulated the data with only 1 simulation model. Benchmarking of diverse populations with different models is warranted for future research. The singletons in the simulated data were filtered before introducing genotyping error; thus, we did not consider the fact that genotyping errors are more likely to happen to singletons.

## Availability of Source Code and Requirements

- Project name: IBD Detection Tool Benchmark Project
- Project homepage: https://github.com/ZhiGroup/IBD_benchmark
- Operating systems: Linux and Windows
- Programming language: C#
- License: MIT
- RRID: SCR_022850
- biotools: ibd_benchmark_tool

## Data Availability

The source code and a set of demonstration data are available at GitHub[37]. The datasets supporting the results of this article are available in the *GigaScience* Database [38]. The UK Biobank Resource is retrieved under Application Number 24247.

The FastSMC [19] software package and source code are available at FastSMC’s GitHub [39]. The hap-IBD [20] software package and source code are available at hap-IBD’s GitHub [40]. The iLash [21] software package and source code are available at iLash’s GitHub [41]. The RaPID [18] software package and source code are available at RaPID’s GitHub [42]. The TPBWT [22] software package and source code are available at TPBWT’s GitHub [43].

### Additional Files

**Supplementary Fig. S1**. Benchmarking results of different tools in EUR data on longer IBD segments without genotyping error.

**Supplementary Fig. S2**. Benchmarking results of different tools in EUR data on longer IBD segments with a 0.1% genotyping error rate.

**Supplementary Fig. S3**. IBD segment coverage in array and sequencing data with a genotyping error rate of 0.1% for 3 different populations. The x-axis represents the site index, and the y-axis denotes the number of IBD segments that covered each site. The ground truth is displayed in black.

**Supplementary Fig. S4**. Benchmarking results of different IBD detection tools in the EUR array and sequencing data with a genotyping error rate of 0%. The length discrepancy is measured in cM.

**Supplementary Fig. S5**. Benchmarking results of different IBD detection tools in the EUR array and sequencing data with a genotyping error rate of 0.2%. The length discrepancy is measured in cM.

**Supplementary Fig. S6**. Benchmarking results of different IBD detection tools in the EUR array and sequencing data with a genotyping error rate of 0.3%. The length discrepancy is measured in cM.

**Supplementary Fig. S7**. Benchmarking results of different IBD detection tools in the EAS array and sequencing data with a genotyping error rate of 0%. The length discrepancy is measured in cM.

**Supplementary Fig. S8**. Benchmarking results of different IBD detection tools in the EAS array and sequencing data with a genotyping error rate of 0.1%. The length discrepancy is measured by cM.

**Supplementary Fig. S9**. Benchmarking results of different IBD detection tools in the EAS array and sequencing data with a genotyping error rate of 0.2%. The length discrepancy is measured in cM.

**Supplementary Fig. S10**. Benchmarking results of different IBD detection tools in the EAS array and sequencing data with a genotyping error rate of 0.3%. The length discrepancy is measured in cM.

**Supplementary Fig. S11**. Benchmarking results of different IBD detection tools in the EAS array and sequencing data with a genotyping error rate of 0.4%. The length discrepancy is measured by cM.

**Supplementary Fig. S12**. Benchmarking results of different IBD detection tools in the AFR array and sequencing data with a genotyping error rate of 0%. The length discrepancy is measured by cM.

**Supplementary Fig. S13**. Benchmarking results of different IBD detection tools in the AFR array and sequencing data with a genotyping error rate of 0.1%. The length discrepancy is measured by cM.

**Supplementary Fig. S14**. Benchmarking results of different IBD detection tools in the AFR array and sequencing data with a genotyping error rate of 0.2%. The length discrepancy is measured by cM. Some lines may be discontinued or dipped due to low power.

**Supplementary Fig. S15**. Benchmarking results of different IBD detection tools in the AFR array and sequencing data with a genotyping error rate of 0.3%. The length discrepancy is measured by cM; other measures are based on percentage. Some lines may be discontinued or dipped due to low power.

**Supplementary Fig. S16**. Benchmarking results of different IBD detection tools in the AFR array and sequencing data with a genotyping error rate of 0.4%. The length discrepancy is measured by cM; other measures are based on percentage. Some lines may be discontinued or dipped due to low power.

**Supplementary Fig. S17**. Benchmarking results of different IBD detection tools in the mixed array and sequencing data without genotyping. The length discrepancy is measured by cM; other measures are based on percentage.

**Supplementary Fig. S18**. Benchmarking results of different IBD detection tools in the mixed array and sequencing data with a genotyping error rate of 0.1%. The length discrepancy is measured by cM; other measures are based on percentage. Some lines may be discontinued or dipped due to low power.

**Supplementary Fig. S19**. Benchmarking results of different IBD detection tools in the mixed array and sequencing data with a genotyping error rate of 0.2%. The length discrepancy is measured by cM; other measures are based on percentage. Some lines may be discontinued or dipped due to low power.

**Supplementary Fig. S20**. Benchmarking results of different IBD detection tools in the mixed array and sequencing data with a genotyping error rate of 0.3%. The length discrepancy is measured by cM; other measures are based on percentage. Some lines may be discontinued or dipped due to low power.

**Supplementary Fig. S21**. Benchmarking results of different IBD detection tools in the mixed array and sequencing data with a genotyping error rate of 0.4%. The length discrepancy is measured by cM; other measures are based on percentage. Some lines may be discontinued or dipped due to low power.

**Supplementary Fig. S22**. Benchmarking results of different tools in the EUR sequencing data with low genotyping error rates.

**Supplementary Fig. S23**. Benchmarking results of different tools in the EAS sequencing data with low genotyping error rates.

**Supplementary Fig. S24**. Benchmarking results of different tools in the AFR sequencing data with low genotyping error rates.

**Supplementary Fig. S25**. Benchmarking results of different tools in Mixed sequencing data with low genotyping error rates.

**Supplementary Fig. S26**. IBD recall of array datasets. Genotyping errors can affect the recall. The x-axis in each subplot denotes the IBD length cutoff. TPBWT and RaPID’s recall values are not impacted significantly.

**Supplementary Fig. S27**. IBD recall of sequencing dataset with low genotyping error rates. Genotyping errors can affect the recall. The x-axis in each subplot denotes the IBD length cutoff. TPBWT and RaPID’s recall values are not impacted significantly.

**Supplementary Fig. S28**. IBD recall of sequencing dataset with high genotyping error rates (≥0.1%). Genotyping errors can affect the recall. The x-axis in each subplot denotes the IBD length cutoff. TPBWT and RaPID’s recall values are not impacted significantly.

**Supplementary Fig. S29**. Relatedness detection results in array and sequencing data without genotyping error rate on 4 population panels. The y-axes are the number of individual pairs found in each category. The ground truths are displayed (black bars) for reference purposes.

**Supplementary Fig. S30**. Relatedness detection results in array data with a 0.1% genotyping error rate and phasing errors (0.31% for AFR, 0.45% for EAS, 0.38% for EUR, and 0.61% for the mixed) on 4 population panels. The y-axes are the number of individual pairs found in each category. “TPBWT PC” denotes the result of TPBWT with phasing error correction enabled. The ground truths are displayed (black bars) for reference purposes.

**Supplementary Fig. S31**. Benchmarking results of different IBD detection tools in AFR array data using the AFR and EUR marker set with a genotyping error rate of 0.1%. The length discrepancy is measured by cM; other measures are based on percentage.

**Supplementary Figure S32**. Benchmarking results of different IBD detection tools in EAS array data using the EAS and EUR marker set with a genotyping error rate of 0.1%. The length discrepancy is measured by cM; other measures are based on percentage.

**Supplementary Table S1**. Parameters and command lines for benchmarking different IBD detection tools.

**Supplementary Table S2**. Effect of phasing error on the absolute delta accuracy of IBD detection tools using relaxed IBD segment cutoff. “TPBWT NPC” represents runs without enabling phasing error correction, and “TPBWT PC” represents runs with phasing error correction enabled. As observed, the effect of phasing error is negligible in our experiment. However, enabling TPBWT phasing error correction reduces the accuracy, since there is a possibility of overcorrection by wrongfully extending/merging adjacent matches.

**Supplementary Table S3**. Effect of phasing error on the absolute delta length accuracy of IBD detection tools using relaxed IBD segment cutoff. “TPBWT NPC” represents runs without enabling phasing error correction, and “TPBWT PC” represents runs with phasing error correction enabled. As observed, the effect of phasing error is negligible in our experiment. However, enabling TPBWT phasing error correction reduces the accuracy, since there is possibility of overcorrection by wrongfully extending/merging adjacent matches.

**Supplementary Table S4**. Effect of phasing error on the absolute delta length discrepancy of IBD detection tools using relaxed IBD segment cutoff. “TPBWT NPC” represents runs without enabling phasing error correction, and “TPBWT PC” represents runs with phasing error correction enabled. As observed, the effect of phasing error is negligible in our experiment. However, enabling TPBWT phasing error correction reduces the accuracy, since there is possibility of overcorrection by wrongfully extending/merging adjacent matches.

**Supplementary Table S5**. Effect of phasing error on the absolute delta power of IBD detection tools using relaxed IBD segment cutoff. “TPBWT NPC” represents runs without enabling phasing error correction, and “TPBWT PC” represents runs with phasing error correction enabled. As observed, the effect of phasing error is negligible in our experiment.

**Supplementary Table S6**. Effect of phasing error on the absolute delta accumulated power of IBD detection tools using relaxed IBD segment cutoff. “TPBWT NPC” represents runs without enabling phasing error correction, and “TPBWT PC” represents runs with phasing error correction enabled. As observed, the effect of phasing error is negligible in our experiment.

**Supplementary Table S7**. Effect of phasing error on the absolute delta power of IBD detection tools using restricted IBD segment cutoff. “TPBWT NPC” represents runs without enabling phasing error correction, and “TPBWT PC” represents runs with phasing error correction enabled. As observed, the effect of phasing error is negligible in our experiment.

**Supplementary Table S8**. CPU time results of a simulated large dataset. The na entry means the tool could not finish the task due to limited resources.

**Supplementary Table S9**. Wall clock time results of a simulated large dataset. The na entry means the tool could not finish the task due to limited resources.

**Supplementary Table S10**. Memory consumption results of a simulated large dataset. The na entry means the tool could not finish the task due to limited resources.

**Supplementary Table S11**. FastSMC run time and memory result on a 6-core 3.5-GHz CPU and 32-GB memory PC. The regression formula used to estimate memory consumption was y = 26026x2 + 82909x + 2E + 06 with *R*^2^ = 0.9989. The predicted memory consumption for UK Biobank whole chromosome 1 was 6.50 TB. The regression formula used to estimate run time was y = 37.574x2 − 80.999x + 294.52 with *R*^2^ = 0.9982. At the end, matched to the server 3.0-GHz clock speed, the final predicted run time was 126 days.

**Supplementary Table S12**. Memory consumption based on UK Biobank chromosome 1 with IBD 3 cM length cutoffs by increasing sample size. The TPBWT’s results were based on the simulated panels. The na entry means the tool cannot finish the task due to limited resources.

**Supplementary Table S13**. Memory consumption based on UK Biobank chromosome 1 with IBD 5 cM length cutoffs by increasing sample size. The TPBWT’s results were based on the simulated panels. The na entry means the tool cannot finish the task due to limited resources.

## Abbreviations

ASMC: ascertained sequentially Markovian coalescent; cM: centiMorgan; DTC: direct to consumer; HMM: hidden Markov model; IBD: identical by descent; IBS: Identity by State; MAF: minor allele frequency; MRCA: most recent common ancestor; PBWT: positional Burrows–Wheeler transform; TMRCA: time to most recent common ancestor; VCF: Variant Call Format.

## Competing Interests

The authors declare that they have no competing interests.

## Funding

This work was supported by the National Institutes of Health grants R01 HG010086 and R56 HG011509.

## Authors’ Contributions

S.Z. and D.Z. conceived and designed the study. K.T., A.N., Y.W., S.Z., and D.Z. developed the method. All authors conducted the analyses and interpretation of the results. All authors contributed to the writing of the manuscript. All authors read and approved the final manuscript.

## Supplementary Material

giac111_GIGA-D-22-00078_Note_on_Original_Submission

giac111_GIGA-D-22-00078_Revision_1

giac111_GIGA-D-22-00078_Revision_2

giac111_GIGA-D-22-00078_Revision_3

giac111_Response_to_Reviewer_Comments_Original_Submission

giac111_Response_to_Reviewer_Comments_Revision_1

giac111_Response_to_Reviewer_Comments_Revision_2

giac111_Reviewer_1_Report_Original_Submission Ying Zhou -- 5/12/2022 Reviewed

giac111_Reviewer_2_Report_Original_Submission Will Freyman -- 5/13/2022 Reviewed

giac111_Reviewer_2_Report_Revision_1 Will Freyman -- 8/19/2022 Reviewed

giac111_Supplemental_File
