# Correction to: Open-source benchmarking of IBD segment detection methods for biobank-scale cohorts

**DOI:** 10.1093/gigascience/giac129

**Published:** 2022-12-29

This is a correction to: Kecong Tang, Ardalan Naseri, Yuan Wei, Shaojie Zhang, Degui Zhi, Open-source benchmarking of IBD segment detection methods for biobank-scale cohorts, GigaScience, Volume 11,2022, giac111, https://doi.org/10.1093/gigascience/giac111

Following article publication, the supplementary file entitled “giac111_GIGA-D-22–00 078_Original_Submission” was removed, in order to remove the benchmarking of TPBWT on the UK Biobank dataset due to potential licensing conflicts. Simulated data for TPBWT has been included as a replacement in the other supplementary data files. The publisher apologizes for initially posting the incorrect file.

