# Supplementary material for: Open-source benchmarking of IBD segment detection methods for biobank-scale cohorts
Source: Gigascience. 2022 Dec 6;11:giac111. doi: 10.1093/gigascience/giac111 (PMC9724555; doi:10.1093/gigascience/giac111)
Supplement: giac111_GIGA-D-22-00078_Note_on_Original_Submission [file giac111_giga-d-22-00078_note_on_original_submission.pdf]

Note on original submission file

Following article publication, the supplementary file entitled "**giac111_GIGA-D-22-00078_Original_Submission**" was updated to remove the benchmarking of TPBWT on the UK Biobank dataset due to potential licensing conflicts. Simulated data for TPBWT has been included as a replacement in the other supplementary data files. The publisher apologizes for initially posting the incorrect file.
