# Supplementary material for: Open-source benchmarking of IBD segment detection methods for biobank-scale cohorts
Source: Gigascience. 2022 Dec 6;11:giac111. doi: 10.1093/gigascience/giac111 (PMC9724555; doi:10.1093/gigascience/giac111)
Supplement: giac111_GIGA-D-22-00078_Revision_1 [file giac111_giga-d-22-00078_revision_1.pdf]

GigaScience

Open-source benchmarking of IBD segment detection methods for biobank-scale cohorts

--Manuscript Draft--

**Manuscript Number:**	GIGA-D-22-00078R1
**Full Title:**	Open-source benchmarking of IBD segment detection methods for biobank-scale cohorts
**Article Type:**	Research
**Funding Information:**	National Human Genome Research Institute (R01 HG010086) — Dr. Degui Zhi<br>National Human Genome Research Institute (R56 HG011509) — Dr. Degui Zhi
**Abstract:**	In the recent biobank era of genetics, the problem of Identical-By-Descent (IBD) segment detection received renewed interest, as IBD segments in large cohorts offer unprecedented opportunities in the study of population and genealogical history, as well as genetic association of long haplotypes. While a new generation of efficient methods for IBD segment detection become available, direct comparison of these methods is difficult: existing benchmarks were often evaluated in different datasets, some are not openly accessible; methods benchmarked were run under sub-optimal parameters; benchmark performance metrics were not defined consistently. Here, we developed a comprehensive and completely open-source evaluation of the power, accuracy, and resource consumption of these IBD segment detection methods using realistic population genetic simulations with various settings. Our results pave the road for fair evaluation of IBD segment detection methods and provide an practical guide for users.
**Corresponding Author:**	Degui Zhi<br>University of Texas Health Science Center at Houston<br>Houston, TX UNITED STATES
**Corresponding Author Secondary Information:**	
**Corresponding Author's Institution:**	University of Texas Health Science Center at Houston
**Corresponding Author's Secondary Institution:**	
**First Author:**	Kecong Tang, MS, Computer Science
**First Author Secondary Information:**	
**Order of Authors:**	Kecong Tang, MS, Computer Science
	Ardalan Naseri, Ph.D, Computer Science
	Yuan Wei
	Shaojie Zhang, Ph.D, Computer Science
	Degui Zhi, Ph.D, Bioinformatics
**Order of Authors Secondary Information:**	
**Response to Reviewers:**	We have addressed all concerns. Please see the two attached files for details: cover letter and response to reviewers' comments
**Additional Information:**	
**Question**	**Response**
Are you submitting this manuscript to a special series or article collection?	No

Powered by Editorial Manager® and ProduXion Manager® from Aries Systems Corporation

**Experimental design and statistics**<br><br>Full details of the experimental design and statistical methods used should be given in the Methods section, as detailed in our Minimum Standards Reporting Checklist. Information essential to interpreting the data presented should be made available in the figure legends.<br><br>Have you included all the information requested in your manuscript?	Yes
**Resources**<br><br>A description of all resources used, including antibodies, cell lines, animals and software tools, with enough information to allow them to be uniquely identified, should be included in the Methods section. Authors are strongly encouraged to cite Research Resource Identifiers (RRIDs) for antibodies, model organisms and tools, where possible.<br><br>Have you included the information requested as detailed in our Minimum Standards Reporting Checklist?	Yes
**Availability of data and materials**<br><br>All datasets and code on which the conclusions of the paper rely must be either included in your submission or deposited in publicly available repositories (where available and ethically appropriate), referencing such data using a unique identifier in the references and in the "Availability of Data and Materials" section of your manuscript.<br><br>Have you have met the above requirement as detailed in our Minimum Standards Reporting Checklist?	Yes

Manuscript

Click here to access/download;Manuscript;manuscript_IBD_Benckmark_Gig

OXFORD (GIGA)^n SCIENCE

GigaScience, 2017, 1–10

doi: xx.xxxx/xxxx
Manuscript in Preparation
Paper

PAPER

Open-source benchmarking of IBD segment detection methods for biobank-scale cohorts

Kecong Tang[1], Ardalan Naseri[2], Yuan Wei[1], Shaojie Zhang[1,*] and Degui Zhi[2,*]

[1]Department of Computer Science, University of Central Florida, Orlando, FL,32816, USA and [2]School of Biomedical Informatics, The University of Texas Health Science Center at Houston, Houston, TX 77030, USA

[*]Shaojie.Zhang@ucf.edu; Degui.Zhi@uth.tmc.edu

Abstract

In the recent biobank era of genetics, the problem of Identical-By-Descent (IBD) segment detection received renewed interest, as IBD segments in large cohorts offer unprecedented opportunities in the study of population and genealogical history, as well as genetic association of long haplotypes. While a new generation of efficient methods for IBD segment detection become available, direct comparison of these methods is difficult: existing benchmarks were often evaluated in different datasets, some are not openly accessible; methods benchmarked were run under sub-optimal parameters; benchmark performance metrics were not defined consistently. Here, we developed a comprehensive and completely open-source evaluation of the power, accuracy, and resource consumption of these IBD segment detection methods using realistic population genetic simulations with various settings. Our results pave the road for fair evaluation of IBD segment detection methods and provide an practical guide for users.

Key words: identical-by-descent; biobank-scale data; IBD segment detection tools; benchmarking

Introduction

Identical-By-Descent (IBD) segments, i.e. DNA segments inherited from a common ancestor [1, 2] provide direct evidence of genetic relatedness. It plays a key role in population genetic research. Given whole-genome genetic data of a cohort of individuals, IBD segments could be used to detect relations among them [3, 4]. IBD segments are used in association analysis and can also be directly used in IBD mapping to detect signals of disease-causing markers in population samples [5, 6, 7, 8, 9]. IBD segments can also be used to estimate missing genotypes from the haplotype or genotype reference panels [10]. IBD segments have also been used to phase genotype data [11, 12]. Direct to consumer (DTC) genetic testing companies use IBD segments to offer services of inferred family history [13]. IBD segments may help to identify individuals without direct access to their genetic data in forensic settings [14].

Several IBD segment detection tools have been developed in the last decade. The early generation of tools were designed to detect IBD segments among hundreds to thousands of individuals from a genotype or a haplotype panel [15, 16, 1, 17]. In the biobank era, with the need for processing hundreds of thousands or even millions of haplotypes, a new generation of efficient IBD segment detection tools have been designed in the past few years [18, 19, 20, 21, 22].

While all these methods claim to be efficient and accurate, a direct and reproducible comparison of these methods is still missing. Each of the methods uses a unique solution to solve the IBD detection problem. As a result, some of the proposed methods may be more suitable for certain settings while it may fail in some other cases. For example, one method may not work well in the presence of high genotyping errors. Moreover, the efficiency of some approaches could be more significant in very large data.

Therefore, in this work, we aim to systematically benchmark the new generation of IBD segment detection methods. To facilitate transparency, reproducibility, and convenience, we choose to leverage the latest advanced population genetics simulation tools. For the quality assessment of the IBD call, we define several metrics. The metrics aim to evaluate the accuracy of IBD calls and the detection power of the tools. We define three accuracy and three power metrics considering coverage and length in both single segment and multiple segments upon three sets of data. Moreover, we evaluate the tools regarding the run time and memory consumption. We measure run times and memory usages with the same abundant resources for all tools with increasing sizes of data input.

Compiled on: August 5, 2022.
Draft manuscript prepared by the author.

Methods

When ground truth IBD segments in real data are available, real data is always preferred. However, benchmarking using real data is limited to only close relatives, and mostly for long IBD segments. With advanced population genetics simulation tools, we can have precise ground truth IBD segments among the whole simulated population. Coalescent simulators have been used to evaluate the IBD calls previously [19, 20, 18]. Here, we also use the coalescent simulation tool msprime [23] to simulate the datasets. To benchmark the power and accuracy of the IBD detection tools, we used simulated data. On the other hand, for the run time and memory usage, we used UK Biobank data. We used the phased haplotype panels to input the IBD segment detection tools for all of our benchmarks.

Simulated datasets

We used msprime v1.0.1 to generate three population data sets: East Asian (EAS), European (EUR), and African (AFR). Additionally we generated a mixed set of EAS, EUR, and AFR. These data sets contain the chromosome 20 sequences of 4,000 individuals (8,000 haplotypes), based on the out-of-Africa population model [24]. We used HapMap phase II GRCh37 [25] as the recombination map and 1.38×10^{-8} as the mutation rate. The true IBD segments are determined as the contiguous segment among the tree sequence generated by msprime, where the haplotype pairs share the same most recent common ancestor (MRCA). We sampled the trees for every 5,000 base pair physical distance, and the true IBD segments were extracted if their genetic lengths are at least 1 centiMorgan (cM). Once the data sets were simulated, we filter out the sites having multiple allele values and singletons.

Then we generated array density data sets by down sampling the original sequencing data sets. To achieve an even marker density, a common practice in marker design, each sequencing panel was first given a target number of markers, we used the number 17,197 same as the number of markers in UK Biobank chromosome 20. Then a centiMorgan interval (I) was calculated from dividing the total genetic length of the chromosome by the desired number of sites, this (I) indicated the ideal distance between two markers. As mentioned, in many cases there may not be any marker in many continuous (I), a window size (w) was considered by attempting to take w markers from a w *by* I range. After that, our program tried to select each marker from its original (I) first, then tried to take a marker from the leftover markers in the w *by* I range if there was not any marker in the original (I). During the marker selection, a site with the highest minor allele frequency (MAF) would be selected. Acquiring perfect even marker density and reaching desired number of markers are two goals with conflict. After multiple runs with different window sizes, we chose 5 as the window size, at the end, we had 15,958 sites in the EAS array data, 15,546 sites in the EUR array data, 15,313 sites in the AFR array data, and 16,013 sites in the mixed array data.

To simulate genotyping errors, we randomly implanted genotyping errors with the rates of 0.1%, 0.2%, 0.3%, and 0.4% per genotype over the variant sites. Although this is a simplification of the realistic error profiles in sequencing data (e.g., false positively called non-variant sites were not included, nucleotide-specific and region-specific error profiles were not modeled), we did include a singleton-filter which we assume would remove most of false positively called non-variants sites. As we observed that several methods are not tolerating errors well in sequencing data, we also generated additional data sets with 0.0125%, 0.025%, and 0.05% error rates for sequencing data. Errors are added incrementally from lower to higher error rated data to aid interpretability.

Furthermore, we also simulated phasing errors using a standard haplotype phasing method to investigate the effect of phasing errors. For the phasing error simulation, we simulated haplotypes of European ancestry using stdpopsim. The HapMap genetic map (GRCh37) was used to simulate haplotypes of chromosome 20. We used the population model OutOfAfrica_2T12 defined in stdpopsim and generated these 2,000 Europeans. Every two consecutive haplotypes were merged into one genotype. Then 0.1% genotyping errors were added to the panel. At the end, SHAPEIT4 [26] was used to re-phase the genotype data without using any reference. At end we had average 0.17% switching error that calculated by VCFtools [27].

UK Biobank dataset

While real data sets are often not suited for evaluating power and accuracy, they can be used to estimate the efficiency of the tools regarding the run time and memory usage. For the run time and memory usage benchmark, we used chromosome 1 of the UK Biobank. This data set contains 487,409 individuals and 53,260 markers with a total file size of 100 GB. We created subsets of the input by reducing the number of individuals from the full size to 250, 125, 62.5, 31.3, and 15.6 thousand to evaluate the scaling up of the methods. Due to potential licence conflicts, we did not run TPBWT on UK Biobank data. A set of simulated panels were created for the run time and memory tests. This data set had similar number of individuals (500,000), similar number of sites (53,161), and tested to have similar number of detected IBD segments from other tools compared to the original UK Biobank chromosome 1.

Evaluation metrics

One of the limitations of existing benchmarks is that they use non-unified definitions of metrics. Here we aim to provide a set of well-defined and standardized metrics. For IBD segment detection performance evaluation, similar to other information retrieval problems [28, 29], we consider two aspects that should be examined together. First is the precision of the reported IBD segments. Second is the ability of the method to recover the true IBD segments. The reported IBD segments cannot simply be evaluated using precision and recall since the reported segments might be partially true or a ground true IBD segment can be partially reported. Moreover, a ground truth IBD segment can be reported as multiple segments. Figure 1 shows the evaluation metrics to assess the quality of IBD segments. In the following subsections, all the metrics are described in detail.

Each evaluation metric compares the sets of ground truth IBD segments and reported IBD segments. For the comparison, one can compare the variant sites, physical locations, or genetic locations. Comparing the genetic locations (in cM) assesses the quality of IBD segment call in a wide range of applications such as genealogical search and association analysis. Considering variant sites may be more suitable for applications such as IBD mapping. However, genetic location would still largely be consistent with the variant site comparison. In this work, we calculated the evaluation measures using the genetic locations of ground truth and reported IBD segments. To observe the performance of different IBD segment lengths, ground truth IBD segments and reported IBD segments were collected as full sets, and binned sets as [2,3), [3,4), [4,5), [5,6), and [7,∞) cM bins. Then the accuracy calculations were carried on by taking each bin set as an evaluation target with the full set of ground truth as the reference set, this method overcomes a major case that a reported IBD segment's length is very close to ground truth IBD segment's length but was binned into different bin, e.g. a 2.9 cM reported IBD segment is binned into the [2,3) cM bin. But the ground truth is 3.0 cM and was divided into the [3,4) cM bin, if we only consider [2,3) cM ground truth set as the reference this almost perfect reported IBD segment will not be counted. The same approach was applied to power calculations, and we considered a full set of reported IBD results as the reference set to compute with

each ground truth bin.

The reason we use these multiple of metrics is that there is no single metric that can describe all aspects of IBD segment detection performance. Indeed, depending on the downstream analysis tasks, different aspects of IBD segment detection are weighted differently. For example, genealogy inference algorithms use the total IBD segment length. Some use the total number of IBD segments, and some use the lengths of individual IBD segments. For IBD mapping, the actual set of markers that are called is more important.

Evaluation of reported segments

In order to evaluate the quality of reported segments, we define three measures: *accuracy*, *length accuracy*, and *length discrepancy*. The *accuracy* is calculated as the number of covered IBD segments divided by the total number of reported IBD segments. A reported IBD segment is considered covered, if at least 50% of its length is covered by a ground truth IBD segment. *Length accuracy* is a more fine-grained measure for reported IBD segments, and the measure accounts for the portions of falsely reported segments along a true IBD segment. To calculate the *length accuracy*, we first find the best-matching ground truth IBD segment for each reported IBD segment, i.e. the one that covers the reported IBD segment with the longest overlap. Next, the percentage of the reported IBD segment covered is calculated. Finally an average of these percentages across all reported IBD segments denotes the *length accuracy*. These accuracy measures can reflect the concept *False Positive Rate* simply by *1 - Accuracy*. *Length discrepancy* captures the length difference between the reported IBD segment and its best-matching true IBD segment. Over all segments, the root-mean-square deviation is calculated as the *length discrepancy*. For this measure, the smaller the *length discrepancy* is, the better the quality of the reported IBD segment. This measure focuses on the length of matching between the reported and the ground true IBD segments.

Detection power

We define three measures to evaluate the ability of the tools in detecting ground true IBD segments. The first measure, *recall*, denotes the proportion of the number of true IBD segments that have been reported. Here, we assume a ground truth IBD segment has been detected if a reported segment covers at least 50% of the ground truth segment. The second measure, *power*, denotes the average proportion of true IBD segments that are covered by its best-matching reported segment. For each ground truth IBD segment, its best-matching reported IBD segment is the one that has the longest overlap. The third measure, *accumulative power*, is similar to the second measure but here we consider multiple reported IBD segments. All reported IBD segments that overlap with a ground true IBD segment are being considered.

IBD coverage distribution

The whole chromosome IBD segment coverage was calculated by counting how many times each site was covered by different tools, we focused on how close each tool matched to ground truth. This visualization method could also be used to observe how each tool handled different regions. On the other hand, it also indicates special regions upon the chromosome. For the ground truth the threshold cut-off was set to 2 cM. As a result, some tools might have slightly higher values especially if they overestimate the IBD boundaries.

Relatedness inference

To evaluate the application of relatedness inference, we simply used the calculated total length of reported IBD segments between each individual pair. The threshold values [30] were calculated by the total IBD sharing to assign the degrees of relatedness up to 4th degree. We did not adjust the threshold values to account for the reduction of power that resulted from genotyping errors. The genotyping error rate was set to 0.1%. No phasing error was introduced for this experiment.

Population and influence of marker selection

We first simulated three separated population panels and a mixed panel that contains all the three populations to observe the performance of each tool upon different population panels. In addition, considering each population has their own set of preferred markers, using a marker set designed for a different population may yield poor results. Therefore we conducted another set of experiment to observe the influence of marker selection. First, three sub panels were extracted from the mixed panel by population. Then, the ideal set of markers for each sub panel was determined using the down sampling method in simulated datasets section. After that, array data sets with all combinations between three populations and three marker sets were created with 0.1% genotyping error rate. At the end we input each tool with all these array data sets to evaluate the performance.

Run time and memory usage

The idea of run time and memory benchmarking was to give practically sufficient resources, and use increasing sizes of sample to input each tool, then observe how each tool's performance. Therefor, for each experiment, we allocated a maximum of 500 GB memory and 60 hyper threading CPU cores with 3.00 GHz clock speed in our computation node. We collected three reported pieces of information from the "sacct" command of Slurm Workload Manager version 20.02.2. The wall clock time corresponds to the "Elapsed", the CPU Time is reflected by "TotalCPU", and peak memory consumption is measured by "MaxRSS".

The wall clock time tells the user how long it takes to get the job done in real world. It gives the most direct impression of a software, regardless of time sensitive cases, however, most people still like to have the job done quickly. The CPU Time, a traditional measure of time complexity in algorithms, measures how much total workload the task really is. With advanced multi-core computer hardware, this number could be much different to the wall clock time. With the same sufficient resources a small job and a massive job could be done in the same amount of real world time, because the big job may take dozens of CPU cores but the small job may just use a fraction of a single core. The Memory consumption is used to reflect the space complexity, since hard drive storage is no longer a major limit, and memory size is the major scale-limiting factor. If a task requires too much memory, it could be practically impossible. On the other hand if an algorithm is efficient enough to solve a big problem in a small size of memory, it means tasks could be done in a practical and low-budget approach. Most importantly this means with the same amount of memory this method can solve the problem on a larger scale. The degree of parallelism describes how well a tool could fully utilize modern computer resources. The degree of parallelism is defined as the ratio of CPU Time and Wall Clock Time. If the number is very close to 1, it means the tool does not take advantage of multi-cores. On the other hand the tool is well parallel programmed if the number is much higher than 1 and even closer to the number of cores allocated. This number could also be divided by the number of cores allocated to represent CPU utilization. This measure indicates the decency of modern software engineering, and it potentially tells how fast the tool could solve the problem in the real world.

Selected tools

We chose the latest generation of IBD segment detection tools that have been designed for biobank data scale in the past few years,

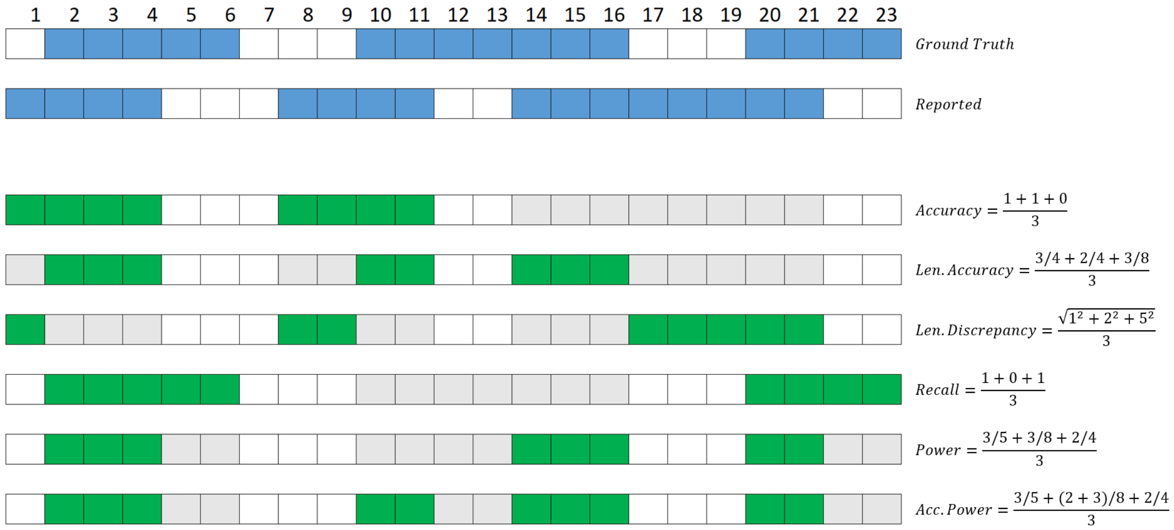

Figure 1. Demonstration of accuracy and power measures. The blue segments represent existing ground truth and reported IBD segments, the green segments donate the segments that are to be considered in calculations and the gray segments are not to be considered during calculations. Accuracy is counted if a reported IBD segment could be covered by any one ground truth IBD segment with 50%, as shown the last reported IBD segment was not considered. Length accuracy is calculated with a best ground truth IBD segment that could cover the reported IBD with maximum length. Length Discrepancy is calculated by considering the length difference with a best matching ground truth IBD segment. Recall is courted if a ground truth IBD segment could be covered with 50%, as demonstrated, the second ground truth IBD segment is not counted since none of those reported IBD segments could cover 50% of this ground truth IBD segment. Power is a similar measure to length accuracy but measures the segments of ground truth IBD segment that are covered by a best reported IBD segment. Accumulative power considers multiple reported IBD segments to cover a ground truth IBD segment.

FastSMC, hap-IBD, iLash, RaPID, and TPBWT. Since each tool has a wide range of parameter combinations, it is practically difficult to acquire the optimal parameter combination for each tool in each case. We spent the same amount of effort on each tool and tried to find proper parameters for different cases. We used default parameters if we could not find better parameters. All the parameters for different tools have been included in Supplementary Table S1.

FastSMC applies hashing methods to identify IBD segments, then uses a coalescent-based HMM (hidden Markovian model) to verify the segments. FastSMC uses an ascertained sequentially Markovian coalescent (ASMC) to estimate the posterior of the time to most recent common ancestor (TMRCA) for pairs of individuals. It has been implemented in C++ and with optional Python bindings. The input Variant Call Format (VCF) file needs to be converted to Oxford phased haplotype file, and the genetic map needs to be processed to have exact sites in the original VCF file. The software requires so-called Decoding quantities files. The files for some populations have been provided in the package.

hap-IBD uses a seed-and-extend technique with positional Burrows–Wheeler transform (PBWT)[31]. Due to the efficient extraction of haplotype matches using the (PBWT) , it is very time and memory efficient. hap-IBD has been implemented in Java, which provides conveniently cross platform execution. It does not require any data conversion of the VCF file. The required genetic map file format is PLINK, while the sites in the map file do not have to be the exact sites in the VCF file. hap-IBD is well engineered to execute in parallel.

iLash applies sliding, minhashing, locality sensitive hashing and pairwise extension to report IBD segments. iLash was programmed in C++, it also supports parallel execution. The input VCF and genetic map files need to be converted to PLINK PED and MAP formats.

RaPID leverages multiple random projections to perform approximate haplotype matching, then applies PBWT and merging methods for IBD detection. RaPID has been implemented in C++. The input file should be in compressed VCF format, and the genetic map needs to be preprocessed to match exact sites in the VCF file. The command line parameters can be calculated with provided python script considering genotyping error rate, marker density, and the minimum length of IBD segments. The author also has recommended a set of parameters for general cases.

TPBWT extends PBWT by adding a new dimension to the PBWT that allows mismatches in detected IBD segments. The added dimension simply masks out potential errors in the haplotypes and extends IBD segments even if there is a mismatch between the haplotypes. The argument template, a two dimensional python list, defines the configurations that can tolerate genotyping and/or phasing errors. Similar to RaPID, TPBWT also requires a genetic map to be formatted to match exact sites in the VCF input file. TPBWT has been implemented in Python.

Results

Overall evaluation of IBD segment detection tools

Figure 2 shows the values for five different metrics in both sequencing and array data: accuracy, length accuracy, length discrepancy, power, and accumulative power. These metrics can be applied to assess the quality of IBD calls for a variety of applications such as IBD mapping, genealogical inference. The other two metrics, recall and length discrepancy metrics are specially useful for applications that summarize the IBD counts and length. e.g. investigation of population history, pedigree inference.

The genotyping error was set to 0.1% which is the expected [32, 33, 34] error rate in available data. As shown in Figure 4, FastSMC and iLash both had high accuracy in 2 cM segments and overall lower power. hap-IBD's accuracy is slightly lower for 2 cM segments, but it has significantly higher detection power. RaPID had high power and competable accuracy on longer segments. Both RaPID and TPBWT have relatively low length discrepancy in both sequencing and array data, and higher detection power in sequencing data. The accuracy of TPBWT in sequencing data is lower, especially for shorter segments. For long segments ($\geq$ 10 cM), all selected tools have high power and accuracy on inputs without genotyping error. When dealing with genotyping errors, reported segments tend to break into pieces resulting in low power. Overall, TPBWT has the highest detection power on sequencing data with genotyping error followed by RaPID (see Supplementary Figures S1 and S2).

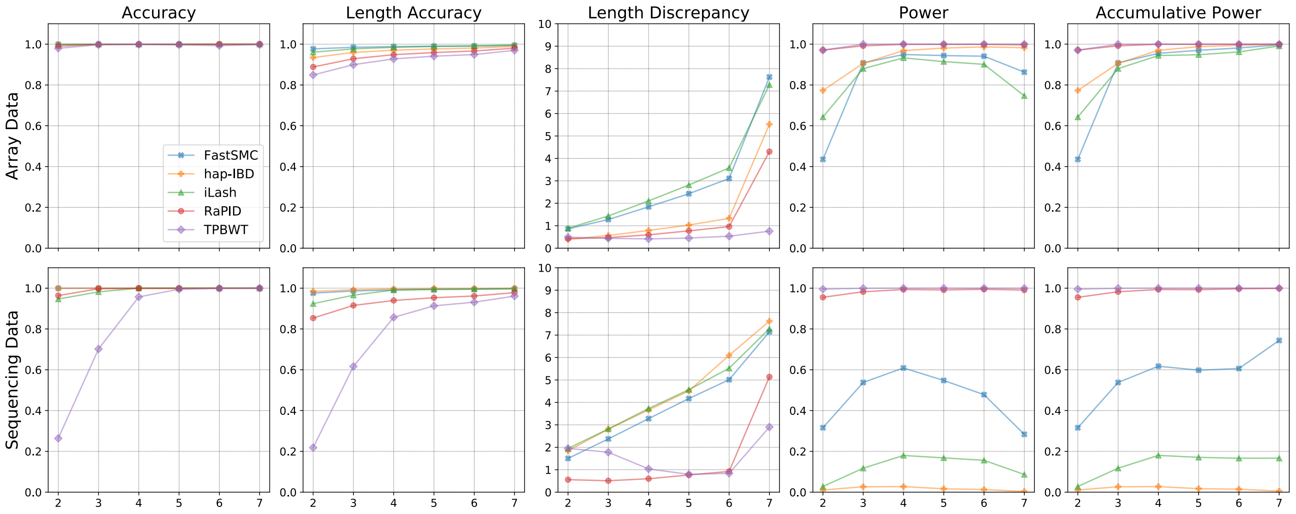

Figure 2. Benchmarking results of different IBD segment detection tools in EUR array and sequencing data with a genotyping error rate of 0.1% stratified by segment lengths. Some tools have reduced detection power as IBD segment cut-off increases. It is likely caused by fractured IBD segments since this measure only considers one reported IBD segment. Accumulative power considers all reported overlap IBD segments and all corresponding values are monotonically increasing. The length discrepancy is measured by cM, other measures are based on percentage.

Regional coverage of IBD segments

Figure 3 shows IBD segments coverage results with 0.1% genotyping error rate. The results of IBD segments coverage without genotyping error are also included in Supplementary Figure S3. Overall, while the ground truth IBD coverage is roughly even across the chromosome, all methods produced IBD calls with variability in coverage. For array data, TPBWT and RaPID have over-calling while iLash and FastSMC have under-calling. hap-IBD has the best calibration in terms of overall coverage. For sequencing data, all methods have much greater variability in IBD coverage. IBD coverage of RaPID was the closest to the ground truth in sequencing data.

Based on our results, we inferred that most of the tools were well-configured to handle array data. hap-IBD and iLASH have very low detection power for sequencing data with errors. We conjecture that the default parameters for these tools do not offer competitive results for sequencing data. We noticed that TPBWT has a much higher false positive rates in EAS and EUR than in AFR, this further tells that either new sets of sequencing-specific parameters are needed or a pre-processing to thin the panel is required.

Effect of genotyping errors

Although most current human genetic panels have very low genotyping error rate, some historic data and some non-human data may still have significantly higher genotyping error rates. Increasing genotyping errors will likely decrease the detection power. Figure 4 shows the results of different tools in both array and sequencing data using a relatively high genotyping error rate (0.4%). The EUR results for 0%, 0.2% and 0.3% genotyping error rates. The results for other two populations and the mixed population are shown in the Supplementary Figures S4-S21. Overall, TPBWT and RaPID seem to be more robust against high genotyping errors while TBPWT's detection power remains higher with slightly lower accuracy compared to RaPID in sequencing data. The detection power of hap-IBD, FastSMC and iLASH are impacted noticeably by higher genotyping errors. The results with lower genotyping errors in sequencing data have been included in the Supplementary Figures S22-S25. The recall results are included in the Supplementary Figures S26-S28. The recall values are overall consistent with the power values. Length discrepancies of RaPID and TPBWT are lower, especially for longer segments in the presence of genotyping errors. This reflects that other methods tend to break long IBD segments into pieces and thus length discrepancy increased significantly.

Relatedness inference

As shown in Figure 5, the simulated data has a realistic distribution of close relatives, where the number of relative pairs increases exponentially with the degree of relatedness. For relatedness inference, we found that, on array data, all methods achieved a decent calls. This is understandable as calling close relatives mainly relies on the accumulated power for long segments, and all methods are very capable of doing that. hap-IBD, TPBWT, and RaPID have closest reported pairs to ground truth. hap-IBD had slightly less reported pairs, while TPBWT and RaPID tended to report a few more pairs. Both FastSMC and iLash tended to report fewer number of pairs in most of the cases while FastSMC tended report more pairs in first degree. On sequencing data, not all methods are well-calibrated. The power of hap-IBD was most-severely reduced, followed by iLash. FastSMC had less impact by genotyping error, had closer number of reported pairs. Both TPBWT and RaPID had decent power, though TPBWT has a tendency of over-calling in 4-th and 3-rd degree relatives in EAS and EUR. All tools had similar good performance on data without genotyping error (See Supplementary Figure S29).

Performance in different populations and marker sets

As shown in Figure 3 and Figure 5, although different populations has different marker density and average IBD coverage, the performance of all methods were relatively stable. A main outlier was that TPBWT has a much higher false positive rates in EAS and EUR than in AFR. This may be due to lower level of linkage disequilibrium in AFR. Based on our observations there was no significant difference between the mixed population panel and the separated panels.

The results on the marker set selection (Supplementary Figures S30 and S31) show that using a set of markers designed for a different population has noticeably lower accuracies, especially in terms of length accuracy and length discrepancy at shorter target lengths. Powers, on the other hand, were slightly increased. This is because that the set of markers designed for a different population has lower MAF than the set of markers designed for the population of interest and thus increases the chance of random allele matches. However, the overall effects of suboptimal marker set selection are minor in

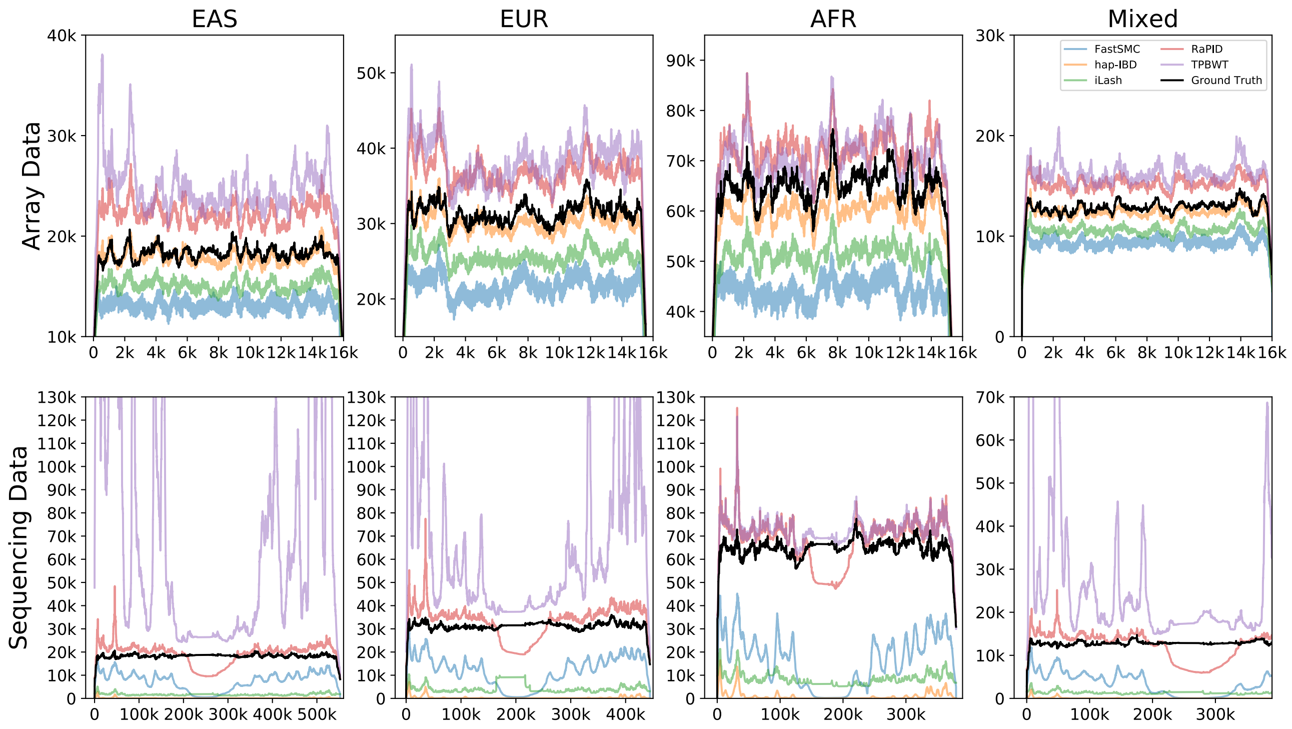

Figure 3. Visualization of IBD segment coverage (over 2 cM) in array and sequencing data with a genotyping error rate of 0.1% on three populations. The x-axises are the marker location indexes. The y-axises are number of IBD segments that covered each marker. The ground truths were displayed (black) for reference purpose.

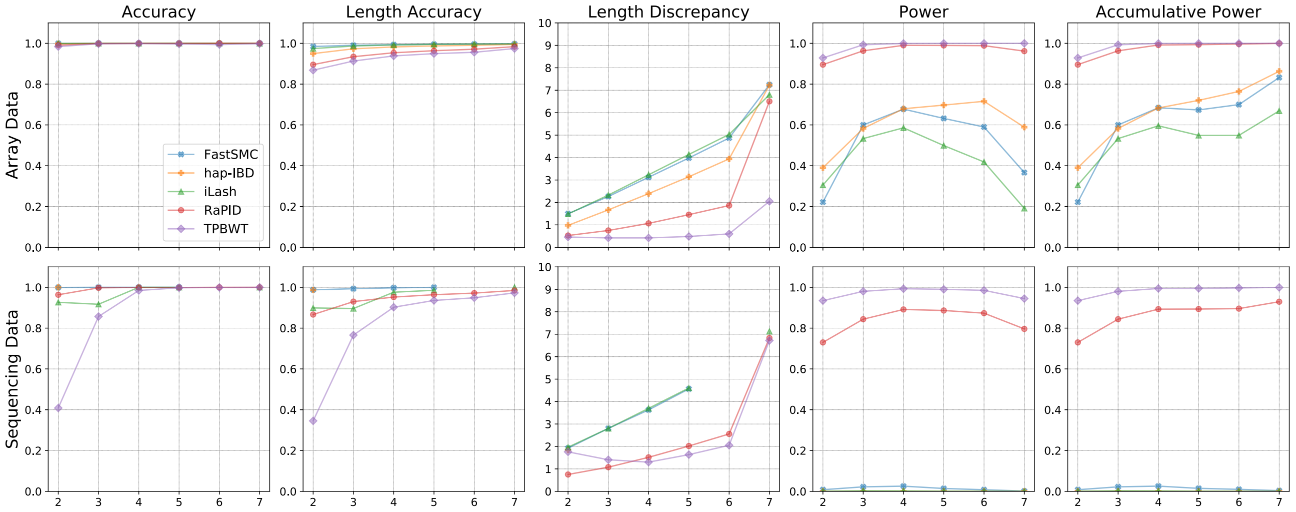

Figure 4. Benchmarking results of different IBD segment detection tools in EUR array and sequencing data with a genotyping error rate of 0.4% stratified by segment lengths. The length discrepancy is measured by cM, other measures are based on percentage. Some lines may be discontinued or dipped due to low power.

our experiments.

Robustness against phasing errors

The benchmarking results for all tools in the presence of phasing error are included in Supplementary Tables S2-S7. The reduction of power is from 4-7% for different tools for short segments (2-3 cM). The reduction in power for very long segments ($\geq$ 15 cM) ranges from 5-20% without any special treatment of phasing errors, with a strict threshold cut-off length. However, including shorter segments results in 4-10% differences in detection power for very long segments. TPBWT with phasing error tolerance had only 5% reduction in detection power for very long segments ($\geq$ 15 cM) with minor reduction in length accuracy. TPBWT with phasing error tolerance mode was also able to increase its detection power for shorter segments by almost 2% for 2 cM segments, while the reduction in accuracy/length accuracy was more noticeable. In general, the reduction of detection power after the data were re-phased is not very significant for short IBD segments. This is due to the high accuracy of the current phasing algorithms with the availability of large biobank scale cohorts. The phased data may contain some long-range switch errors or blips, but they will not contribute to a noticeable reduction in the detection power except for strict and very long IBD cut-off thresholds. The possible reduction of power can also be alleviated for some downstream analysis, especially if the total shared IBDs between two individuals is of interest [35]. However, the impact of phasing errors could be more consequential if the number of segments is being considered.

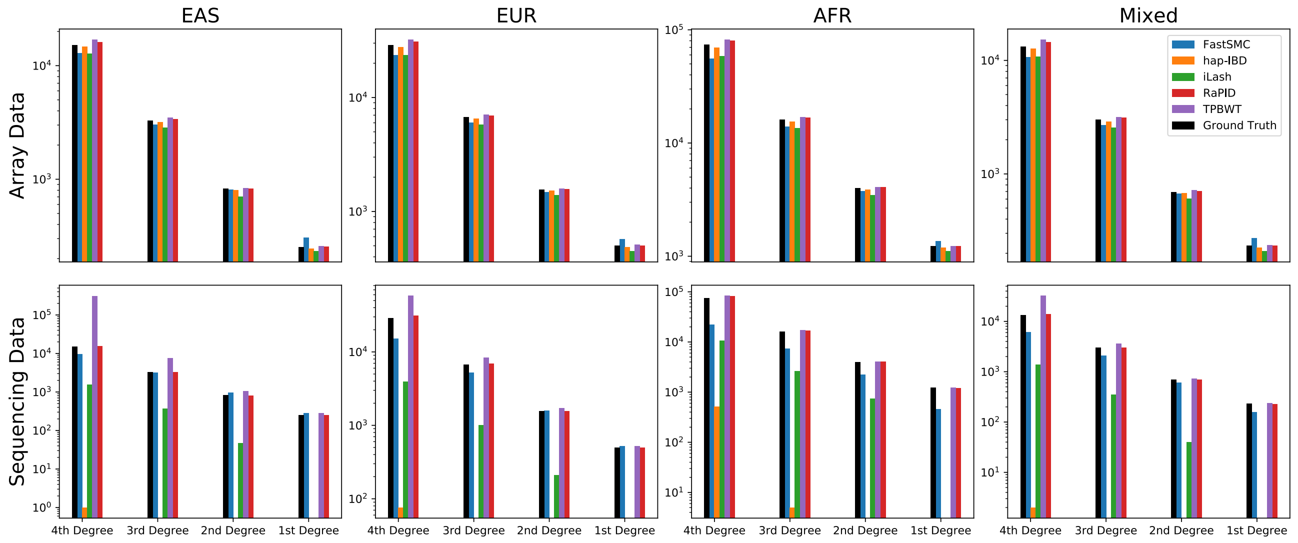

Figure 5. Relatedness detection results in array and sequencing data with a genotyping error rate of 0.1% on three populations. The y-axises are number of individual pairs were found in each category. The ground truths were displayed (black bars) for reference purpose.

Run time and memory useage

The run time and memory consumption experiments were carried out on our 500 GB memory server as mentioned, iLash and TPBWT could not finish all the experiments with 500 GB memory. Due to potential licence conflicts we did not run TPBWT on UK Biobank data, the time and memory consumption results for TPBWT were based on the simulated dataset as mentioned in the method section. Since FastSMC requires the most recent updated operating system and libraries, this could be a problem since most of the servers do not use the latest versions. We were able to make FastSMC executable on a 32 GB memory PC for accuracy and power assessment through some efforts. Thus, we were not able to run FastSMC for large panels. We ran FastSMC on panels with smaller sample sizes (from 1,000 to 31,000) on the 32 GB PC. By measuring run times and memory consumptions of these smaller inputs, with an extrapolation using second order polynomial regressions, we estimated the FastSMC run time would be 126 days and memory consumption would be 6.5 TB for the whole chromosome 1 of UK Biobank data. Therefore we did not include FastSMC in Figure 6. More details can be found in the Supplementary Table S8.

hap-IBD had the shortest wall clock time due to the efficient underlying method and efficient parallelization. It took around 0.5 hours for hap-IBD to complete the IBD segment calls for UK Biobank chromosome 1 with 2 cM IBD cut-off. iLash won the second place on wall-clock time, but as mentioned it could not finish some large cases. Both hap-IBD and iLash take a good advantage of parallel execution. hap-IBD was able to utilize about 75% of the CPU resource as the degree of parallelization of 45.27 out of 60. iLash reached an average parallelization of 9.23 out of 60. CPU time of RaPID was the shortest among all the tools for longer segments (e.g. 5 cM). hap-IBD had the lowest CPU time for short IBD segments (2 cM). The CPU time for TPBWT was also noticeably short for smaller panels (e.g. 15 K) but the time increased fast with the increasing sample size. iLash and TPBWT did not complete the task with the largest panels within the provided memory.

As shown in Tables 1 and 2, RaPID requires the least amount of memory. The memory consumption of RaPID was limited to less than 8 GB, while some other tools required hundreds of gigabytes of memory. hap-IBD also has an efficient memory usage with the maximum memory usage of about 112 GB memory. The memory consumption results for 3 cM and 5 cM length cut-offs can be found in Supplementary Tables S12 and S13. We observed the memory consumption of hap-IBD and iLash for the simulated data set doubled compared to the original UK Biobank data, so we predict that TPBWT could finish the original 250 thousand individual subset of UK Biobank chromosome 1. The run time and memory consumption results for this simulated set can be found in Supplementary Tables S8-S10.

Conclusion and Discussion

We conducted the first open-source fully-transparent comprehensive benchmarking of modern efficient IBD segment detection methods. Based on our benchmarking, most tools yield decent results in most settings, even in the presence of phasing errors. However, genotyping error rates and marker densities would affect the performance of IBD detection methods in different degrees.

For short IBD segments (e.g. 2 cM), FastSMC and iLash have high accuracies but low power in both sequencing and array density. Both hap-IBD and RaPID have high power and relatively lower accuracy in array data, and TPBWT achieved highest detection power in sequencing data followed by RaPID, especially in the presence of high genotyping errors. For longer IBD segments ($\geq 5cM$), all five tools have comparable accuracy, while hap-IBD, RaPID and TPBWT have high power in array data. iLASH and FastSMC may report IBD segments as multiple short segments especially for longer segments (e.g. 5 cM). The accuracy of most tools for long segments ($\geq$ 10 cM) is high but the detection power of some tools is affected significantly with genotyping errors. TPBWT and RaPID have the highest detection power for sequencing data in the presence of genotyping errors, with TPBWT being the most robust against high genotyping error rates.

In general, the minimum length threshold impacts the accuracy as reflected in our results. We acknowledge that the methods are more likely to report an IBS as an IBD segment for shorter segments. The minimum threshold length can vary for different applications for example for genealogical search long IBD segments (e.g. 5 or 7 cM) may be used, while for IBD mapping, shorter IBD segment (e.g. 2 or 3 cM) might be used. Our aim was to benchmark the performance of different tools for different IBD segment lengths that provide the researchers with the expected accuracy for their applications.

FastSMC, hap-IBD and iLASH have high accuracy in sequencing data especially for short segments (e.g. 2 cM), but their detection power is low. The accuracy of all tools are relatively high for long IBD segments (e.g. 5 cM). Genotyping errors in the sequencing data

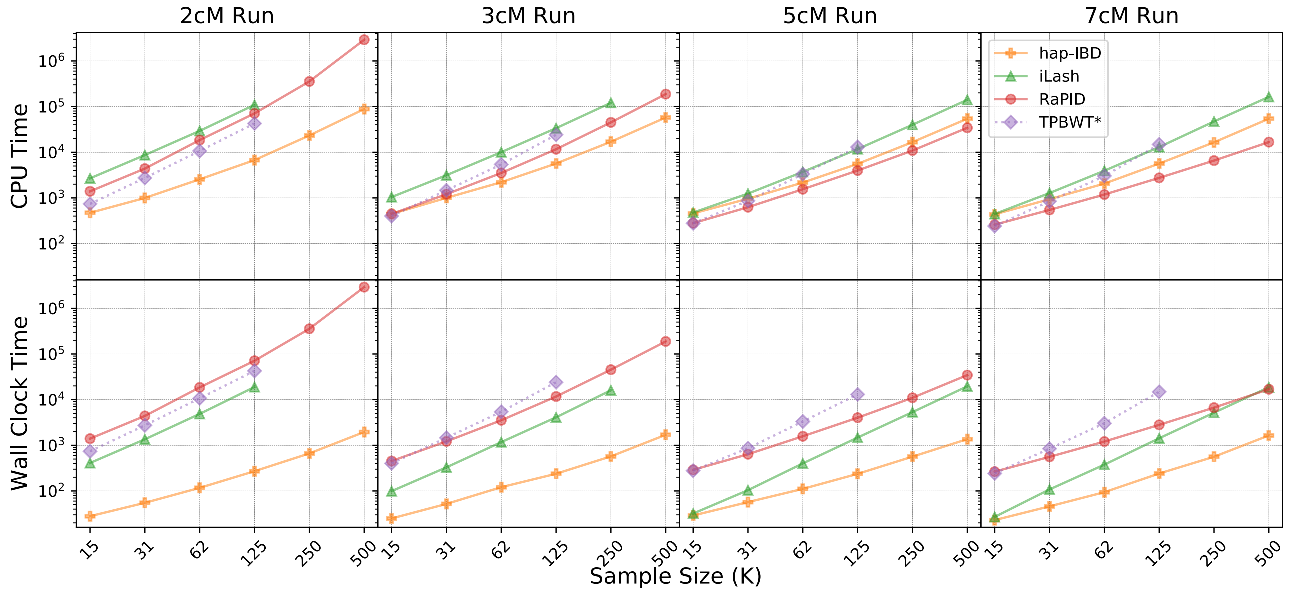

Figure 6. Run time results based on UK Biobank Chromosome 1. CPU Time and Wall Clock Time are displayed in the first and second row. Each column of the subfigures represents runs with different IBD segment cut-off (2, 3, 5, and 7 cM). The x-axises in each subfigure are the input size in thousand individuals. The TPBWT's results were based on the simulated panels.

Table 1. Memory consumptions based on UK Biobank chromosome 1 with 2 cM length cut-offs.

	Sample Size (K)					
	15	31	62	125	250	500
hap-IBD	1.76 MB	7.88 GB	8.79 GB	22.81 GB	59.03 GB	110.15 GB
iLash	7.85 GB	20.05 GB	58.73 GB	191.46 GB	na	na
RaPID	728.72 MB	1.03 GB	1.54 GB	2.47 GB	4.14 GB	7.25 GB
TPBWT*	7.70 GB	30.59 GB	122.16 GB	488.40 GB	na	na

Table 2. Memory consumptions based on UK Biobank chromosome 1 with 7 cM length cut-offs.

	Sample Size (K)					
	15	31	62	125	250	500
hap-IBD	1.20 MB	9.93 GB	23.24 GB	15.62 GB	31.78 GB	110.03 GB
iLash	1.77 MB	5.39 GB	15.25 GB	30.59	77.48 GB	178.83 GB
RaPID	347.13 MB	718.97 MB	1.22 GB	2.21 GB	3.90 GB	6.33 GB
TPBWT*	7.70 GB	30.60 GB	122.16 GB	488.40 GB	na	na

may affect the results of hap-IBD, FastSMC and iLASH significantly. A possible solution to run these tools could be to down sampling the data using MAF.

Most IBD segment detection tools can handle VCF files. hap-IBD does not require any data preparation while RaPID and TPBWT require minor data preparation. FastSMC and iLash require some data conversion if the available data are in VCF format. FastSMC also requires additional files which are population specific.

Regarding the resource requirements, only RaPID and hap-IBD were able to be used with limited resources, while others require significantly more memory for large panels. RaPID was the most memory efficient tool which took 12 times less memory than hap-IBD. On the other hand, hap-IBD had the highest resource utilization followed by iLash. hap-IBD was also the fastest method for short IBD cut-off lengths in our experiments, while RaPID had less CPU Time for longer IBD cut-off (e.g. 5 cM).

Our study has several limitations. First, as mentioned, the parameter combination for each tool may not be optimal for each case. Finding out the best combinations for each tool is beyond the scope of this work. However, we open-sourced our entire benchmarking protocol and thus we hope others can contribute and release their optimized parameters on the same data sets. Second, although simulated data sets are somehow reflected in the real world, there is still a possibility that the real data could have some unique characteristics. In general, it is not straightforward to extract ground true IBD segments from real data. The availability of several pedigree data may provide the opportunity to extract ground truth from real data. The same evaluation metrics, however, then can be used to evaluate the performance of IBD segment detection tools. Moreover, we only simulated the data with one simulation model. Benchmarking of diverse populations with different models is warranted for future research. The singletons in the simulated data were filtered before introducing genotyping error, thus, we did not consider the fact that genotyping errors are more likely to happen to singletons.

Availability of Source Code and Requirements

- Project name: IBD Detection Tool Benchmark Project
- Project home page: https://github.com/ZhiGroup/IBD_benchmark
- Operating systems: Linux and Windows

- Programming language: C#
- License: MIT

Availability of Supporting Data and Materials

The source code and a set of demonstration data is available at: https://github.com/ZhiGroup/IBD_benchmark. The datasets supporting the results of this article are available in the *Giga*DB database.

The FastSMC [19] software package and source code are available at: https://github.com/PalamaraLab/FastSMC. The hap-IBD [20] software package and source code are available at: https://github.com/browning-lab/hap-ibd. The iLash [21] software package and source code are available at: https://github.com/roohy/iLASH. The RaPID [18] software package and source code are available at: https://github.com/ZhiGroup/RaPID. The TPBWT [22] software package and source code are available at: https://github.com/23andMe/phasedibd

Additional Files

Supplementary Figures S1 and S2 Benchmarking results of different tools in EUR data on longer IBD segments.
Supplementary Figure S3. Visualization of IBD segment coverage in array and sequencing data without genotyping on three populations.
Supplementary Figures S4-S21. Benchmarking results of EUR results for 0%, 0.2% and 0.3% genotyping error rates, and results for EAS, AFR, and mixed populations.
Supplementary Figures S22-S25. Benchmarking results of sequencing data with low genotyping error rates.
Supplementary Figures S26-S28. Benchmarking results of IBD segments recall.
Supplementary Figure S29. Relationship detection results in array and sequencing data without genotyping error on EAS, EUR, AFR, and mixed populations.
Supplementary Figures S30 and S31. Benchmarking results of different IBD detection tools on AFR and EAS array data using a set of markers designed for a different population.
Supplementary Table S1. Parameters and command lines for benchmarking different IBD detection tools.
Supplementary Tables S2-S7. Effect of phasing error on the performance of IBD detection tools.
Supplementary Tables S8-S10. Run time and memory result on simulated big panels.
Supplementary Table S11. FastSMC run time and memory usage results on a 6 core 3.5 GHz CPU and 32 GB memory PC.
Supplementary Tables S12 and S13. Memory usage based on UK Biobank Chromosome 1 with IBD 3 cM and 5 cM length cut-offs by increasing sample size.

Declarations

Abbreviations

ASMC: ascertained sequentially Markovian coalescent; cM: centi-Morgan; DTC: direct to consumer; HMM: hidden Markov model; IBD: Identical-By-Descent; MAF: minor allele frequency; MRCA: most recent common ancestor; PBWT: positional Burrows–Wheeler transform; TMRCA: time to most recent common ancestor; VCF: Variant Call Format.

Consent for publication

Not applicable.

Competing interests

The authors declare that they have no competing interests.

Funding

This work was supported by the National Institutes of Health grants R01 HG010086 and R56 HG011509.

Authors' contributions

SZ and DZ conceived and designed the study. KT, AN, YW, SZ, and DZ developed the method. All authors conducted the analyses and interpretation of the results. All authors contributed to the writing of the manuscript. All authors read and approved the final manuscript.

Acknowledgement

The authors acknowledge Olivia Tyndall at the University Writing Center of University of Central Florida for her professional English editing advice. This research has been conducted using the UK Biobank Resource under Application Number 24247.

References

1. Browning BL, Browning SR. A fast, powerful method for detecting identity by descent. The American Journal of Human Genetics 2011;88(2):173–182.
2. Thompson EA. Identity by descent: variation in meiosis, across genomes, and in populations. Genetics 2013;194(2):301–326.
3. Ramstetter MD, Dyer TD, Lehman DM, Curran JE, Duggirala R, Blangero J, et al. Benchmarking relatedness inference methods with genome-wide data from thousands of relatives. Genetics 2017;207(1):75–82.
4. Hill WG, Weir BS. Variation in actual relationship as a consequence of Mendelian sampling and linkage. Genetics research 2011;93(1):47–64.
5. Chen H, Naseri A, Zhi D. FiMAP: A Fast Identity-by-Descent Mapping Test for Biobank-scale Cohorts. medRxiv 2021;.
6. Browning SR, Thompson EA. Detecting rare variant associations by identity-by-descent mapping in case-control studies. Genetics 2012;190(4):1521–1531.
7. Houwen RH, Baharloo S, Blankenship K, Raeymaekers P, Juyn J, Sandkuijl LA, et al. Genome screening by searching for shared segments: mapping a gene for benign recurrent intrahepatic cholestasis. Nature genetics 1994;8(4):380–386.
8. Gusev A, Kenny EE, Lowe JK, Salit J, Saxena R, Kathiresan S, et al. DASH: a method for identical-by-descent haplotype mapping uncovers association with recent variation. The American Journal of Human Genetics 2011;88(6):706–717.
9. Vacic V, Ozelius LJ, Clark LN, Bar-Shira A, Gana-Weisz M, Gurevich T, et al. Genome-wide mapping of IBD segments in an Ashkenazi PD cohort identifies associated haplotypes. Human molecular genetics 2014;23(17):4693–4702.
10. Abney M, ElSherbiny A. Kinpute: using identity by descent to improve genotype imputation. Bioinformatics 2019;35(21):4321–4326.
11. Loh PR, Palamara PF, Price AL. Fast and accurate long-range phasing in a UK Biobank cohort. Nature genetics 2016;48(7):811–816.
12. Delaneau O, Zagury JF, Robinson MR, Marchini JL, Dermitzakis ET. Accurate, scalable and integrative haplotype estimation. Nature communications 2019;10(1):1–10.
13. Henn BM, Hon L, Macpherson JM, Eriksson N, Saxonov S, Pe'er

I, et al. Cryptic distant relatives are common in both isolated and cosmopolitan genetic samples. PloS one 2012;7(4):e34267.
14. Guerrini CJ, Robinson JO, Petersen D, McGuire AL. Should police have access to genetic genealogy databases? Capturing the Golden State Killer and other criminals using a controversial new forensic technique. PLoS biology 2018;16(10):e2006906.
15. Purcell S, Neale B, Todd-Brown K, Thomas L, Ferreira MA, Bender D, et al. PLINK: a tool set for whole-genome association and population-based linkage analyses. The American journal of human genetics 2007;81(3):559–575.
16. Gusev A, Lowe JK, Stoffel M, Daly MJ, Altshuler D, Breslow JL, et al. Whole population, genome-wide mapping of hidden relatedness. Genome research 2009;19(2):318–326.
17. Browning BL, Browning SR. Improving the accuracy and efficiency of identity-by-descent detection in population data. Genetics 2013;194(2):459–471.
18. Naseri A, Liu X, Tang K, Zhang S, Zhi D. RaPID: ultra-fast, powerful, and accurate detection of segments identical by descent (IBD) in biobank-scale cohorts. Genome biology 2019;20(1):1–15.
19. Nait Saada J, Kalantzis G, Shyr D, Cooper F, Robinson M, Gusev A, et al. Identity-by-descent detection across 487,409 British samples reveals fine scale population structure and ultra-rare variant associations. Nature communications 2020;11(1):1–15.
20. Zhou Y, Browning SR, Browning BL. A fast and simple method for detecting identity-by-descent segments in large-scale data. The American Journal of Human Genetics 2020;106(4):426–437.
21. Shemirani R, Belbin GM, Avery CL, Kenny EE, Gignoux CR, Ambite JL. Rapid detection of identity-by-descent tracts for mega-scale datasets. Nature communications 2021;12(1):1–13.
22. Freyman WA, McManus KF, Shringarpure SS, Jewett EM, Bryc K, 23, et al. Fast and robust identity-by-descent inference with the templated positional Burrows–Wheeler transform. Molecular Biology and Evolution 2021;38(5):2131–2151.
23. Kelleher J, Etheridge AM, McVean G. Efficient coalescent simulation and genealogical analysis for large sample sizes. PLoS computational biology 2016;12(5):e1004842.
24. Gutenkunst RN, Hernandez RD, Williamson SH, Bustamante CD. Inferring the joint demographic history of multiple populations from multidimensional SNP frequency data. PLoS genetics 2009;5(10):e1000695.
25. Consortium IH, et al. A second generation human haplotype map of over 3.1 million SNPs. Nature 2007;449(7164):851.
26. Delaneau O, Zagury JF, Robinson MR, Marchini JL, Dermitzakis ET. Accurate, scalable and integrative haplotype estimation. Nature communications 2019;10(1):1–10.
27. Danecek P, Auton A, Abecasis G, Albers CA, Banks E, DePristo MA, et al. The variant call format and VCFtools. Bioinformatics 2011;27(15):2156–2158.
28. Buckland M, Gey F. The relationship between recall and precision. Journal of the American society for information science 1994;45(1):12–19.
29. Zuva K, Zuva T. Evaluation of information retrieval systems. AIRCC's International Journal of Computer Science and Information Technology 2012;4(3):35–43.
30. Manichaikul A, Mychaleckyj JC, Rich SS, Daly K, Sale M, Chen WM. Robust relationship inference in genome-wide association studies. Bioinformatics 2010;26(22):2867–2873.
31. Durbin R. Efficient haplotype matching and storage using the positional Burrows–Wheeler transform (PBWT). Bioinformatics 2014;30(9):1266–1272.
32. Saunders IW, Brohede J, Hannan GN. Estimating genotyping error rates from Mendelian errors in SNP array genotypes and their impact on inference. Genomics 2007;90(3):291–296.
33. Yuan M, Fang H, Zhang H. Correcting for differential genotyping error in genetic association analysis. Journal of human genetics 2013;58(10):657–666.
34. Wall JD, Tang LF, Zerbe B, Kvale MN, Kwok PY, Schaefer C, et al. Estimating genotype error rates from high-coverage next-generation sequence data. Genome research 2014;24(11):1734–1739.
35. Naseri A, Shi J, Lin X, Zhang S, Zhi D. RAFFI: Accurate and fast familial relationship inference in large scale biobank studies using RaPID. PLoS genetics 2021;17(1):e1009315.

Click here to access/download
Supplementary Material
R1_Supplementary_IBD_BM_20220804.docx

Highlight of changes

Click here to access/download
Supplementary Material
Highlighted_IBD_Benckmark_GigSci_R1.pdf

 Click here to access/download;Personal Cover;R_coverletter_IBD_BM (1).pdf

4th August 2022

Dear Editor,

We modified the draft following the reviewers' suggestions. All details that the reviewers' concern about were explained in the response letter and mentioned in the main text.

Here are the additional experiments based the suggestions:

1. IBD coverage plots and relatedness graphs were included.
2. We re-designed the phasing error experience, results are updated.
3. Results of additional populations are included.

We have also made following additional changes beyond the reviews' suggestions:

1. Downsampling Sequencing Data

   We found our previous downsampling method that converts sequencing data to array density data did not consider marker density, which could result in very high marker density in a very small cM range. We referenced with UK Biobank array data marker density, further improved downsampling method to generate more even marker density similar to UK Biobank data. All results are updated, however we did not recognize a significant difference in results. The new method is introduced in the main text and has been uploaded to GitHub.

2. Running TPBWT on UK Biobank Data

   Due to potential license conflicts, we can not run or publish TPBWT results on the UK Biobank data. In this case, simulated data that are similar to the UK Biobank chromosome 1 were generated to test memory and run time particularly for TPBWT. This data set has a similar number of individuals, similar number of sites, and similar number of IBD segments. The TPBWT results in the run time and memory section are replaced with these results and specially marked and explained. Time and memory results of other tools with this new simulated data set are included in the supplementary.

Thank you for your time and consideration.

Sincerely,

Click here to access/download;Personal Cover;Respose_IBD Benchmark GigaSci R1.pdf

Point-by-point responses to the reviewers' comments

Reviewer #1:

Tang et. al presented an excellent work of benchmarking IBD segment detection from biobank-scale cohort, by comparing statistic power and computation efficiency, the strength and weakness of several state-of-the-art software have been well established. This work timely meets the need of applying IBD detection in large data set, and the importance is significant in both method development and downstream applications. This manuscript is well organized, and the writing is easy to follow up. I have only a few addressable comments:

1) The way to simulate genotyping errors need to be more detailed. In real data, genotyping errors are more like to be singletons rather than common variants. It is not clear here that whether adding genotyping errors to the simulated genotypes would result in singletons or not. Correct me if I missed this part.

Response 1:
We added detailed descriptions on how to generate genotyping errors. The singletons were filtered out before introducing genotyping error. The errors are random based, we did not weight the selection of location. We added this limitation in the discussion.

2) IBD coverage rates along the genome and hist plot of IBD length distribution, would be a good compensation to the metrics when comparing different software. This can be done between the true and the detected IBD segments in the simulation data and among different software in real data.

Response 2:
Thank you for the suggestion. We added the histogram plots of IBD coverage rates distribution and also degrees of relatedness figures. These figures are now Figure 3 and Figure 5 in the main text, and supplementary Figure S3 and Figure S23.

The discussion in main text is in the new section "Regional coverage of IBD segments":
"Figure 3 shows IBD segments coverage results with 0.1\% genotyping error rate. The results of IBD segments coverage without genotyping error are also included in Supplementary Figure S3. Overall, while the ground truth IBD coverage is roughly even across the chromosome, all methods produced IBD calls with variability in coverage. For array data, TPBWT and RaPID have over-calling while iLash and FastSMC have under-calling. hap-IBD has the best calibration in terms of overall coverage. For sequencing data, all methods have much greater variability in IBD coverage. IBD coverage of RaPID was the closest to the ground truth in sequencing data.

Based on our results, we inferred that most of the tools were well-configured to handle array data. hap-IBD and iLASH have very low detection power for sequencing data with errors. We conjecture that the default parameters for these tools do not offer competitive results for sequencing data. We noticed that TPBWT has a much higher false positive rates in EAS and

EUR than in AFR, this further tells that either new sets of sequencing-specific parameters are needed or a pre-processing to thin the panel is required."

And in "Relatedness inference" section:
"As shown in figure 5, the simulated data has a realistic distribution of close relatives, where the number of relative pairs increases exponentially with the degree of relatedness.
For relatedness inference, we found that, on array data, all methods achieved a decent calls. This is understandable as calling close relatives mainly relies on the accumulated power for long segments, and all methods are very capable of doing that. hap-IBD, TPBWT, and RaPID have closest reported pairs to ground truth. hap-IBD had slightly less reported pairs, while TPBWT and RaPID tended to report a few more pairs. Both FastSMC and iLash tended to report fewer number of pairs in most of the cases while FastSMC tended report more pairs in first degree. On sequencing data, not all methods are well-calibrated. The power of hap-IBD was most-severely reduced, followed by iLash. FastSMC had less impact by genotyping error, had closer number of reported pairs. Both TPBWT and RaPID had decent power, though TPBWT has a tendency of over-calling in 4-th and 3-rd degree relatives in EAS and EUR. All tools had similar good performance on data without genotyping error (See Supplementary Figure S23)."

Reviewer #2:

In this well written article Tang et al. benchmark identity-by-descent (IBD) inference methods. The authors are to be commended for their transparent and reproducible benchmarking approach. While the analyses and results presented are carefully done and a promising start, there are a few major limitations that reduce the utility of the performed benchmarking when applied to biobank-scale cohorts. With some improvements to the benchmarking I think this will be a tremendously useful addition to the IBD inference literature. Thanks to all the authors for their good work!

Evaluation metrics do not capture false positive IBD

Diverse biobank cohorts include populations with varying allele and haplotype frequencies, so genotype data can be prone to SNP acquisition biases that cause false positive IBD among individuals from some populations. It is crucial to determine the false positive rate for IBD segments as a function of IBD segment length. As the segments get shorter, methods are more likely to mistake IBD for IBS (identical-by-state). What minimum length threshold should researchers use?
Why do the evaluations presented here stop at 2 cM when the simulations include true IBD segments down to 1 cM? None of the metrics considered capture the amount of IBD that is erroneously detected in regions of the genome that were not truly IBD.

Response 3:

Thank you for your comments that have led us to add more details about the evaluation metrics.

The defined accuracy and length accuracy capture false positives. Accuracy (we referred to as hit accuracy sometimes) checks whether a reported IBD segment is covered by any ground truth by at least 50%. This metric is able to identify false positives while ignoring the possible overextensions of reported segments. We also defined a fine grained Length Accuracy which measures each reported IBD segment by length of coverage coinciding only one true IBD (with the largest overlap). The latter will capture the proportion of false positives for each reported IBD. The “false positive” rate could be represented by (1 - Accuracy).

We mention this in “Evaluation of reported segments” section:
“These accuracy measures can reflect the concept False Positive Rate simply by 1 - Accuracy”

In this work, we used 1 cM segments from the ground truth to include all possible 50% overlaps since our minimum length for the benchmark was 2 cM. We benchmarked different IBD detection methods using different minimum thresholds. We added the following paragraph in the Discussion section:
“In general, the minimum length threshold impacts the accuracy as reflected in our results. We acknowledge that the methods are more likely to report an IBS as an IBD segment for shorter segments. The minimum threshold length can vary for different applications, for example for genealogical search long IBD segments (e.g. 5 or 7 cM) may be used, while for IBD mapping, shorter IBD segments (e.g. 2 or 3 cM) might be used. Our aim was to benchmark the performance of different tools for different IBD segment lengths that provide the researchers with the expected accuracy for their applications.”

We evaluated >=2cM reported IBDs with >=1cM ground truth, because we consider a common edge case such as a reported IBD is 2cM but the ground truth is 1.9 cM, without considering IBDs that >=1cM, this almost perfect reported IBD will not be counted.
We clarified this in “Evaluation metrics” section, second paragraph:
“this method overcomes a major case that a reported IBD segment’s length is very close to ground truth IBD segment’s length but was binned into different bin, e.g. a 2.9 cM reported IBD segment is binned into the [2,3) cM bin. But the ground truth is 3.0 cM and was divided into the [3,4) cM bin, if we only consider [2,3) cM ground truth set as the reference this almost perfect reported IBD segment will not be counted.”

Evaluation metrics: “length accuracy” vs. “length discrepancy”

The authors describe two separate metrics “length accuracy” and “length discrepancy”. The “length discrepancy” metric is the more intuitive and useful of these metrics since it simply “captures the length difference between the reported IBD segment and its best-matching true IBD segment.” However this useful metric is not illustrated in the otherwise nice and helpful figure 1, and the results are for some reason relegated to the supplementary material. It is striking that all the methods except RaPID and TPBWT have such high length discrepancies as the IBD segments get longer. Why are they so high? This important finding should be included in the main manuscript.

Response 4:
If a method breaks a long segment into smaller pieces then the length discrepancy will be high. We added discussions on length discrepancy and moved it to maintext.

Evaluating performance along the full range of relatedness

Massive biobank cohorts include individuals along the full range of relatedness: extremely distant relatives who only share 1-7 cM total, parent-child relationships that share ~3700 cM, and everything in between. However, this manuscript focuses exclusively on very distant relatedness; all the metrics shown are evaluated between 1 and 7 cM in segment length. Power, recall, and length discrepancy all show interesting patterns as segments get longer; how do the methods perform for those who share segments longer than 7 cM?

It would be informative to describe the range of relatedness within the simulated dataset. What is the distribution of total pairwise IBD sharing across the dataset? It would be useful to ensure that the simulated dataset includes individuals along the full range of relatedness, and expand the evaluation metrics to include longer IBD segments.

Response 5:
First, we clarify that this work was focused on evaluating individual IBDs, the binned results show 2,3,4,5,6 and IBDs that are **greater or equal to 7cM**. This is clarified in the revision at Evaluation metrics section second paragraph. We created bins (10cM, 15cM, 20cM etc.) for longer segments, but we found most tool performance well in longer segments, it is now mentioned in both “Overall evaluation of IBD segment detection tools” section and “Conclusion and Discussion” section.

At the end of “Overall evaluation of IBD segment detection tools” second paragraph:
“For long segments (>= 10 cM), all selected tools have high power and accuracy on inputs without genotyping error. When dealing with genotyping errors, reported segments tend to break into pieces resulting in low power. Overall, TPBWT has the highest detection power on sequencing data with genotyping error followed by RaPID (see Supplementary Figures S1 and S2)”

In “Conclusion and Discussion” section second paragraph:
“The accuracy of most tools for long segments (>= 10 cM) is high but the detection power of some tools is affected significantly with genotyping errors. TPBWT and RaPID have the highest

detection power for sequencing data in the presence of genotyping errors, with TPBWT being the most robust against high genotyping error rates."

We add pairwise total IBD length distribution measures to evaluate relatedness detections. We defined relationships: Parent/offspring, second degree, third degree, fourth degree.

Robustness against phasing errors

Please report the phase switch error rate of the simulated dataset, and ensure that the switch error rates are realistic and similar to those described elsewhere. For example, in the TPBWT paper we reported that our simulations had a "mean switch error rate of 0.25%, comparable with switch error rates measured elsewhere (Choi et al. 2018)."

The manuscript authors found that phasing had no impact on IBD detection performance. They write that this "is due to the high accuracy of the current phasing algorithms with the availability of large biobank scale cohorts." In my experience, even with state-of-the-art phasing methods like SHAPEIT4 and Eagle2 and very large reference panels, phase switch errors cause significant problems when analyzing large biobank cohorts. Switch errors break up IBD segments into multiple short fragments scattered among the haplotypes of the two individuals, causing high levels of false negative (missing) IBD (see figure 3 in our TPBWT paper for an example).

Short IBD segments are less impacted by phase switch errors – a switch error uniformly occurring along the chromosome is less likely to occur within a short segment compared to a long segment. Since the performance evaluations in this paper focused exclusively on very short segments (1-7 cM), it is not surprising that the authors found phasing had no detectable impact.

As described above, biobank-scale cohorts include individuals along the full range of relatedness. IBD methods should be evaluated along that full range of relatedness too. Please evaluate the impact of phase switch errors when detecting longer IBD segments, particularly the amount of false negative IBD detected.

Response 6:
We recognized the average error rate in the initial data was too low compared with 0.25%. We recreated the phasing error data set and re-run all related tests , with shapeit 4 without giving any reference. Now the average switching error rate reported by vcftools was 0.17%. Different from the genotyping data set, the measures for phasing error data were calculated by individual matching instead of haplotype,since the haplotype orders are not preserved and IBD segments between two individuals are of more interest rather than their specific haplotypes. We also added the bin ranges to [5,7.5), [7.5,10),[10,12.5) and [12.5,15). New results are included in the "Robustness against phasing errors" section and the supplementary table S2 - S7:

“The benchmarking results for all tools in the presence of phasing error are included in Supplementary Tables S2-S7. The reduction of power is from 4-7\% for different tools for short segments (2-3 cM). The reduction in power for very long segments (>= 15 cM) ranges from 5-20% without any special treatment of phasing errors, with a strict threshold cut-off length. However, including shorter segments results in 4-10\% differences in detection power for very long segments. TPBWT with phasing error tolerance had only 5\% reduction in detection power for very long segments (>=15 cM) with minor reduction in length accuracy. TPBWT with phasing error tolerance mode was also able to increase its detection power for shorter segments by almost 2% for 2 cM segments, while the reduction in accuracy/length accuracy was more noticeable.
In general, the reduction of detection power after the data were re-phased is not very significant for short IBD segments. This is due to the high accuracy of the current phasing algorithms with the availability of large biobank scale cohorts. The phased data may contain some long-range switch errors or blips, but they will not contribute to a noticeable reduction in the detection power except for strict and very long IBD cut-off thresholds. The possible reduction of power can also be alleviated for some downstream analysis, especially if the total shared IBDs between two individuals is of interest. However, the impact of phasing errors could be more consequential if the number of segments is being considered.”

Simulating diverse datasets

The simulated datasets only included individuals from the European population. While some biobank cohorts are relatively homogeneous, many are already highly diverse and growing increasingly diverse over time. As mentioned above, diverse cohorts have different allele/haplotype frequencies and thus SNP acquisition biases that may cause false positive IBD in some populations. SNPs that are variable in one population and therefore informative for IBD detection in that population may be fixed in another population. Please consider simulating more diverse datasets that better reflect the biobank cohorts of the present and the future.

Response 7:
Addationtion to EUR, we simulated additional three data sets: EAS, AFR, and a mixed set of EUR, EAS and AFR. The results of relation detection and IBD distributions are added to the main text, and for power and accuracy results are added in supplemental. We did not observe any significant difference between each separated population and the mixed population.

We also carried on a new set of experiments on data with different marker sets based on populations to elevate SNP acquisition biases. We found that using the markers designed for a different population may hurt the accuracies. However, powers, on the other hand, were slightly increased. This is because that the set of markers designed for a different population has lower MAF than the set of markers designed for the population of interest and thus increases the chance of random allele matches. However, the overall effects of suboptimal marker set selection are minor in our experiments even for detecting 2cM segments. These are mentioned in the method section and result section.

The use of default parameter values for each method

Each method, presumably, has a range of parameter values that allow it to perform better under different scenarios (low/high genotyping errors, sequence data vs genotype data, low/high phasing errors). When we released the TPBWT software, the default values were optimized for genotyping data with realistic genotyping/phasing error rates. We would not use the default parameter values to analyze sequence data as is done in this manuscript. Changing the parameter values would result in better accuracy with sequence data.

Additionally, the power and accumulative power of hap-IBD and iLash is surprisingly low when using sequence data. This seems alarming, and suggests that maybe there are parameter settings in these algorithms that were intended to be modified for sequence data to improve performance.

While it is perhaps out of the scope of this manuscript to test each method with a wide range of parameter values, at the very minimum this should be discussed in the manuscript.

Response 8:
We added a clarification regarding the low detection power of iLASH and hap-IBD for sequencing data in "Regional coverage of IBD segments" second paragraph:
"Based on our results, we inferred that most of the tools were well-configured to handle array data. hap-IBD and iLASH have very low detection power for sequencing data with errors. We conjecture that the default parameters for these tools do not offer competitive results for sequencing data. We noticed that TPBWT has a much higher false positive rates in EAS and EUR than in AFR, this further tells that either new sets of sequencing-specific parameters are needed or a pre-processing to thin the panel is required."

We added clarification in both "selected tools":
"We chose the latest generation of IBD segment detection tools that have been designed for biobank data scale in the past few years, FastSMC, hap-IBD, iLash, RaPID, and TPBWT. Since each tool has a wide range of parameter combinations, it is practically difficult to acquire the optimal parameter combination for each tool in each case. We spent the same amount of effort on each tool and tried to find proper parameters for different cases. We used default parameters if we could not find better parameters."

Also in "discussion":
"First, as mentioned, the parameter combination for each tool may not be optimal for each case. Finding out the best combinations for each tool is beyond the scope of this work. However, we open-sourced our entire benchmarking protocol and thus we hope others can contribute and release their optimized parameters on the same data sets."

Run time and memory usage for TPBWT

This may seem a minor quibble, but the way TPBWT was run by the authors negatively impacts the run time and memory usage reported for TPBWT. The authors used these commands:
ibd_results =
tpbwt.compute_ibd(haplotypes,L_f=float(sys.argv[1]),use_phase_correction=False)
ibd_results.to_csv(path+"T_"+sys.argv[2]+"_"+sys.argv[1]+"cM.csv")
This results in holding all the IBD segments in memory during the entire analysis which uses a lot of memory. Additionally, after the compute_ibd command is done running, pandas must loop through the resulting DataFrame to print out the results, which is slow.

Instead the authors should use the segments_out_path argument:
ibd_results =
tpbwt.compute_ibd(haplotypes,L_f=float(sys.argv[1]),use_phase_correction=False,
segments_out_path=”ibd_segments.csv”)
This way TPBWT will write segments directly to disk as they are detected, like other methods do. This is both quicker and more memory efficient. This is documented in the README at https://github.com/23andMe/phasedibd

Response 9:
Thank you for pointing this out. We re-ran TPBWT with these recommended parameters, included these parameters into supplementary Table 1, and results are updated.
