# Supplementary material for: Open-source benchmarking of IBD segment detection methods for biobank-scale cohorts
Source: Gigascience. 2022 Dec 6;11:giac111. doi: 10.1093/gigascience/giac111 (PMC9724555; doi:10.1093/gigascience/giac111)
Supplement: giac111_GIGA-D-22-00078_Revision_3 [file giac111_giga-d-22-00078_revision_3.pdf]

GigaScience

Open-source benchmarking of IBD segment detection methods for biobank-scale cohorts

--Manuscript Draft--

**Manuscript Number:**	GIGA-D-22-00078R3
**Full Title:**	Open-source benchmarking of IBD segment detection methods for biobank-scale cohorts
**Article Type:**	Research
**Funding Information:**	National Human Genome Research Institute (R01 HG010086) — Dr. Degui Zhi<br>National Human Genome Research Institute (R56 HG011509) — Dr. Degui Zhi
**Abstract:**	In the recent biobank era of genetics, the problem of Identical-By-Descent (IBD) segment detection received renewed interest, as IBD segments in large cohorts offer unprecedented opportunities in the study of population and genealogical history, as well as genetic association of long haplotypes. While a new generation of efficient methods for IBD segment detection become available, direct comparison of these methods is difficult: existing benchmarks were often evaluated in different datasets, some are not openly accessible; methods benchmarked were run under sub-optimal parameters; benchmark performance metrics were not defined consistently. Here, we developed a comprehensive and completely open-source evaluation of the power, accuracy, and resource consumption of these IBD segment detection methods using realistic population genetic simulations with various settings. Our results pave the road for fair evaluation of IBD segment detection methods and provide an practical guide for users.
**Corresponding Author:**	Degui Zhi<br>University of Texas Health Science Center at Houston<br>Houston, TX UNITED STATES
**Corresponding Author Secondary Information:**	
**Corresponding Author's Institution:**	University of Texas Health Science Center at Houston
**Corresponding Author's Secondary Institution:**	
**First Author:**	Kecong Tang, MS, Computer Science
**First Author Secondary Information:**	
**Order of Authors:**	Kecong Tang, MS, Computer Science
	Ardalan Naseri, Ph.D, Computer Science
	Yuan Wei
	Shaojie Zhang, Ph.D, Computer Science
	Degui Zhi, Ph.D, Bioinformatics
**Order of Authors Secondary Information:**	
**Response to Reviewers:**	See our cover letter
**Additional Information:**	
**Question**	**Response**
Are you submitting this manuscript to a special series or article collection?	No

Powered by Editorial Manager® and ProduXion Manager® from Aries Systems Corporation

**Experimental design and statistics**<br><br>Full details of the experimental design and statistical methods used should be given in the Methods section, as detailed in our Minimum Standards Reporting Checklist. Information essential to interpreting the data presented should be made available in the figure legends.<br><br>Have you included all the information requested in your manuscript?	Yes
**Resources**<br><br>A description of all resources used, including antibodies, cell lines, animals and software tools, with enough information to allow them to be uniquely identified, should be included in the Methods section. Authors are strongly encouraged to cite Research Resource Identifiers (RRIDs) for antibodies, model organisms and tools, where possible.<br><br>Have you included the information requested as detailed in our Minimum Standards Reporting Checklist?	Yes
**Availability of data and materials**<br><br>All datasets and code on which the conclusions of the paper rely must be either included in your submission or deposited in publicly available repositories (where available and ethically appropriate), referencing such data using a unique identifier in the references and in the "Availability of Data and Materials" section of your manuscript.<br><br>Have you have met the above requirement as detailed in our Minimum Standards Reporting Checklist?	Yes

manuscript source file
Click here to access/download;LaTeX - Main Document (TeX file);main.tex

```
This is pdfTeX, Version 3.14159265-2.6-1.40.21 (TeX Live 2020/W32TeX)
(preloaded format=pdflatex 2020.5.12)  18 OCT 2022 13:13
entering extended mode
 restricted \write18 enabled.
 %&-line parsing enabled.
**main.tex
(./main.tex
LaTeX2e <2020-02-02> patch level 5
L3 programming layer <2020-05-05> (./oup-contemporary.cls
Document Class: oup-contemporary 2017/06/28, v1.1
(c:/TeXLive/2020/texmf-dist/tex/latex/base/article.cls
Document Class: article 2019/12/20 v1.4l Standard LaTeX document class
(c:/TeXLive/2020/texmf-dist/tex/latex/base/size10.clo
File: size10.clo 2019/12/20 v1.4l Standard LaTeX file (size option)
)
\c@part=\count167
\c@section=\count168
\c@subsection=\count169
\c@subsubsection=\count170
\c@paragraph=\count171
\c@subparagraph=\count172
\c@figure=\count173
\c@table=\count174
\abovecaptionskip=\skip47
\belowcaptionskip=\skip48
\bibindent=\dimen134
) (c:/TeXLive/2020/texmf-dist/tex/latex/base/inputenc.sty
Package: inputenc 2018/08/11 v1.3c Input encoding file
\inpenc@prehook=\toks15
\inpenc@posthook=\toks16
) (c:/TeXLive/2020/texmf-dist/tex/latex/base/fontenc.sty
Package: fontenc 2020/02/11 v2.0o Standard LaTeX package
) (c:/TeXLive/2020/texmf-dist/tex/generic/iftex/ifpdf.sty
Package: ifpdf 2019/10/25 v3.4 ifpdf legacy package. Use iftex instead.
(c:/TeXLive/2020/texmf-dist/tex/generic/iftex/iftex.sty
Package: iftex 2020/03/06 v1.0d TeX engine tests
)) (c:/TeXLive/2020/texmf-dist/tex/latex/microtype/microtype.sty
Package: microtype 2019/11/18 v2.7d Micro-typographical refinements (RS)
(c:/TeXLive/2020/texmf-dist/tex/latex/graphics/keyval.sty
Package: keyval 2014/10/28 v1.15 key=value parser (DPC)
\KV@toks@=\toks17
)
\MT@toks=\toks18
\MT@count=\count175
LaTeX Info: Redefining \textls on input line 790.
\MT@outer@kern=\dimen135
LaTeX Info: Redefining \textmicrotypecontext on input line 1354.
\MT@listname@count=\count176
(c:/TeXLive/2020/texmf-dist/tex/latex/microtype/microtype-pdftex.def
File: microtype-pdftex.def 2019/11/18 v2.7d Definitions specific to
pdftex (RS)

LaTeX Info: Redefining \lsstyle on input line 914.
LaTeX Info: Redefining \lslig on input line 914.
```

```
\MT@outer@space=\skip49
)
Package microtype Info: Loading configuration file microtype.cfg.
(c:/TeXLive/2020/texmf-dist/tex/latex/microtype/microtype.cfg
File: microtype.cfg 2019/11/18 v2.7d microtype main configuration file
(RS)
)) (c:/TeXLive/2020/texmf-dist/tex/latex/euler/euler.sty
Package: euler 1995/03/05 v2.5
Package: `euler' v2.5 <1995/03/05> (FJ and FMi)
LaTeX Font Info:    Redeclaring symbol font `letters' on input line 35.
LaTeX Font Info:    Encoding `OML' has changed to `U' for symbol font
(Font)              `letters' in the math version `normal' on input line
35.
LaTeX Font Info:    Overwriting symbol font `letters' in version `normal'
(Font)                  OML/cmm/m/it --> U/eur/m/n on input line 35.
LaTeX Font Info:    Encoding `OML' has changed to `U' for symbol font
(Font)              `letters' in the math version `bold' on input line
35.
LaTeX Font Info:    Overwriting symbol font `letters' in version `bold'
(Font)                  OML/cmm/b/it --> U/eur/m/n on input line 35.
LaTeX Font Info:    Overwriting symbol font `letters' in version `bold'
(Font)                  U/eur/m/n --> U/eur/b/n on input line 36.
LaTeX Font Info:    Redeclaring math symbol \Gamma on input line 47.
LaTeX Font Info:    Redeclaring math symbol \Delta on input line 48.
LaTeX Font Info:    Redeclaring math symbol \Theta on input line 49.
LaTeX Font Info:    Redeclaring math symbol \Lambda on input line 50.
LaTeX Font Info:    Redeclaring math symbol \Xi on input line 51.
LaTeX Font Info:    Redeclaring math symbol \Pi on input line 52.
LaTeX Font Info:    Redeclaring math symbol \Sigma on input line 53.
LaTeX Font Info:    Redeclaring math symbol \Upsilon on input line 54.
LaTeX Font Info:    Redeclaring math symbol \Phi on input line 55.
LaTeX Font Info:    Redeclaring math symbol \Psi on input line 56.
LaTeX Font Info:    Redeclaring math symbol \Omega on input line 57.
\symEulerFraktur=\mathgroup4
LaTeX Font Info:    Overwriting symbol font `EulerFraktur' in version
`bold'
(Font)                  U/euf/m/n --> U/euf/b/n on input line 63.
LaTeX Info: Redefining \oldstylenums on input line 85.
\symEulerScript=\mathgroup5
LaTeX Font Info:    Overwriting symbol font `EulerScript' in version
`bold'
(Font)                  U/eus/m/n --> U/eus/b/n on input line 93.
LaTeX Font Info:    Redeclaring math symbol \aleph on input line 97.
LaTeX Font Info:    Redeclaring math symbol \Re on input line 98.
LaTeX Font Info:    Redeclaring math symbol \Im on input line 99.
LaTeX Font Info:    Redeclaring math delimiter \vert on input line 101.
LaTeX Font Info:    Redeclaring math delimiter \backslash on input line
103.
LaTeX Font Info:    Redeclaring math symbol \neg on input line 106.
LaTeX Font Info:    Redeclaring math symbol \wedge on input line 108.
LaTeX Font Info:    Redeclaring math symbol \vee on input line 110.
LaTeX Font Info:    Redeclaring math symbol \setminus on input line 112.
LaTeX Font Info:    Redeclaring math symbol \sim on input line 113.
LaTeX Font Info:    Redeclaring math symbol \mid on input line 114.
```

```
LaTeX Font Info:    Redeclaring math delimiter \arrowvert on input line
116.
LaTeX Font Info:    Redeclaring math symbol \mathsection on input line
117.
\symEulerExtension=\mathgroup6
LaTeX Font Info:    Redeclaring math symbol \coprod on input line 125.
LaTeX Font Info:    Redeclaring math symbol \prod on input line 125.
LaTeX Font Info:    Redeclaring math symbol \sum on input line 125.
LaTeX Font Info:    Redeclaring math symbol \intop on input line 130.
LaTeX Font Info:    Redeclaring math symbol \ointop on input line 131.
LaTeX Font Info:    Redeclaring math symbol \braceld on input line 132.
LaTeX Font Info:    Redeclaring math symbol \bracerd on input line 133.
LaTeX Font Info:    Redeclaring math symbol \bracelu on input line 134.
LaTeX Font Info:    Redeclaring math symbol \braceru on input line 135.
LaTeX Font Info:    Redeclaring math symbol \infty on input line 136.
LaTeX Font Info:    Redeclaring math symbol \nearrow on input line 153.
LaTeX Font Info:    Redeclaring math symbol \searrow on input line 154.
LaTeX Font Info:    Redeclaring math symbol \nwarrow on input line 155.
LaTeX Font Info:    Redeclaring math symbol \swarrow on input line 156.
LaTeX Font Info:    Redeclaring math symbol \Leftrightarrow on input line
157.
LaTeX Font Info:    Redeclaring math symbol \Leftarrow on input line 158.
LaTeX Font Info:    Redeclaring math symbol \Rightarrow on input line
159.
LaTeX Font Info:    Redeclaring math symbol \leftrightarrow on input line
160.
LaTeX Font Info:    Redeclaring math symbol \leftarrow on input line 161.
LaTeX Font Info:    Redeclaring math symbol \rightarrow on input line
163.
LaTeX Font Info:    Redeclaring math delimiter \uparrow on input line
166.
LaTeX Font Info:    Redeclaring math delimiter \downarrow on input line
168.
LaTeX Font Info:    Redeclaring math delimiter \updownarrow on input line
170.
LaTeX Font Info:    Redeclaring math delimiter \Uparrow on input line
172.
LaTeX Font Info:    Redeclaring math delimiter \Downarrow on input line
174.
LaTeX Font Info:    Redeclaring math delimiter \Updownarrow on input line
176.
LaTeX Font Info:    Redeclaring math symbol \leftharpoonup on input line
177.
LaTeX Font Info:    Redeclaring math symbol \leftharpoondown on input
line 178.

LaTeX Font Info:    Redeclaring math symbol \rightharpoonup on input line
179.
LaTeX Font Info:    Redeclaring math symbol \rightharpoondown on input
line 180
.
LaTeX Font Info:    Redeclaring math delimiter \lbrace on input line 182.
LaTeX Font Info:    Redeclaring math delimiter \rbrace on input line 184.
\symcmmigroup=\mathgroup7
```

```
LaTeX Font Info:    Overwriting symbol font `cmmigroup' in version `bold'
(Font)                  OML/cmm/m/it --> OML/cmm/b/it on input line 200.
LaTeX Font Info:    Redeclaring math accent \vec on input line 201.
LaTeX Font Info:    Redeclaring math symbol \triangleleft on input line
202.
LaTeX Font Info:    Redeclaring math symbol \triangleright on input line
203.
LaTeX Font Info:    Redeclaring math symbol \star on input line 204.
LaTeX Font Info:    Redeclaring math symbol \lhook on input line 205.
LaTeX Font Info:    Redeclaring math symbol \rhook on input line 206.
LaTeX Font Info:    Redeclaring math symbol \flat on input line 207.
LaTeX Font Info:    Redeclaring math symbol \natural on input line 208.
LaTeX Font Info:    Redeclaring math symbol \sharp on input line 209.
LaTeX Font Info:    Redeclaring math symbol \smile on input line 210.
LaTeX Font Info:    Redeclaring math symbol \frown on input line 211.
LaTeX Font Info:    Redeclaring math accent \grave on input line 245.
LaTeX Font Info:    Redeclaring math accent \acute on input line 246.
LaTeX Font Info:    Redeclaring math accent \tilde on input line 247.
LaTeX Font Info:    Redeclaring math accent \ddot on input line 248.
LaTeX Font Info:    Redeclaring math accent \check on input line 249.
LaTeX Font Info:    Redeclaring math accent \breve on input line 250.
LaTeX Font Info:    Redeclaring math accent \bar on input line 251.
LaTeX Font Info:    Redeclaring math accent \dot on input line 252.
LaTeX Font Info:    Redeclaring math accent \hat on input line 254.
) (c:/TeXLive/2020/texmf-dist/tex/latex/merriweather/merriweather.sty
Package: merriweather 2019/10/13 (Bob Tennent) Supports
Merriweather(Sans) font
s for all LaTeX engines.
(c:/TeXLive/2020/texmf-dist/tex/generic/iftex/ifxetex.sty
Package: ifxetex 2019/10/25 v0.7 ifxetex legacy package. Use iftex
instead.
) (c:/TeXLive/2020/texmf-dist/tex/generic/iftex/ifluatex.sty
Package: ifluatex 2019/10/25 v1.5 ifluatex legacy package. Use iftex
instead.
) (c:/TeXLive/2020/texmf-dist/tex/latex/base/textcomp.sty
Package: textcomp 2020/02/02 v2.0n Standard LaTeX package
) (c:/TeXLive/2020/texmf-dist/tex/latex/xkeyval/xkeyval.sty
Package: xkeyval 2014/12/03 v2.7a package option processing (HA)
(c:/TeXLive/2020/texmf-dist/tex/generic/xkeyval/xkeyval.tex
(c:/TeXLive/2020/te
xmf-dist/tex/generic/xkeyval/xkvutils.tex
\XKV@toks=\toks19
\XKV@tempa@toks=\toks20
)
\XKV@depth=\count177
File: xkeyval.tex 2014/12/03 v2.7a key=value parser (HA)
)) (c:/TeXLive/2020/texmf-dist/tex/latex/base/fontenc.sty
Package: fontenc 2020/02/11 v2.0o Standard LaTeX package
) (c:/TeXLive/2020/texmf-dist/tex/latex/fontaxes/fontaxes.sty
Package: fontaxes 2014/03/23 v1.0d Font selection axes
LaTeX Info: Redefining \upshape on input line 29.
LaTeX Info: Redefining \itshape on input line 31.
LaTeX Info: Redefining \slshape on input line 33.
LaTeX Info: Redefining \swshape on input line 35.
```

```
LaTeX Info: Redefining \scshape on input line 37.
LaTeX Info: Redefining \sscshape on input line 39.
LaTeX Info: Redefining \ulcshape on input line 41.
LaTeX Info: Redefining \textsw on input line 47.
LaTeX Info: Redefining \textssc on input line 48.
LaTeX Info: Redefining \textulc on input line 49.
)) (c:/TeXLive/2020/texmf-dist/tex/latex/mathastext/mathastext.sty
Package: mathastext 2019/11/16 v1.3w Use the text font in math mode (JFB)
\mst@exists@muskip=\muskip16
\mst@forall@muskip=\muskip17
\mst@prime@muskip=\muskip18
\mst@do@nonletters=\toks21
\mst@do@easynonletters=\toks22
\mst@do@az=\toks23
\mst@do@AZ=\toks24
\symmtoperatorfont=\mathgroup8
\symmtletterfont=\mathgroup9
** ! and ?
** punctuation: , . : ; and \colon
LaTeX Info: Redefining \relbar on input line 787.
LaTeX Info: Redefining \rightarrowfill on input line 790.
LaTeX Info: Redefining \leftarrowfill on input line 795.
** + and =
LaTeX Info: Redefining \Relbar on input line 886.
** adding = ; and + to \nfss@catcodes
** parentheses ( ) [ ] and slash /
** alldelims: < > \backslash \setminus | \vert \mid \{ and \}
LaTeX Font Info:    Redeclaring math delimiter \backslash on input line
932.
LaTeX Font Info:    Redeclaring math symbol \setminus on input line 944.
LaTeX Info: Redefining \models on input line 953.
** \# \mathdollar \% \&
** \imath and \jmath
LaTeX Font Info:    Overwriting math alphabet `\mathnormalbold' in
version `nor
mal'
(Font)                  T1/Merriweather-OsF/b/it --> T1/Merriweather-
OsF/b/it o
n input line 2140.
LaTeX Font Info:    Overwriting math alphabet `\mathnormalbold' in
version `bol
d'
(Font)                  T1/Merriweather-OsF/b/it --> T1/Merriweather-
OsF/b/it o
n input line 2140.
LaTeX Font Info:    Overwriting symbol font `mtletterfont' in version
`normal'
(Font)                  T1/Merriweather-OsF/m/it --> T1/Merriweather-
OsF/m/it o
n input line 2140.
LaTeX Font Info:    Overwriting symbol font `mtletterfont' in version
`bold'
(Font)                  T1/Merriweather-OsF/m/it --> T1/Merriweather-
OsF/b/it o
```

```
n input line 2140.
LaTeX Font Info:    Overwriting symbol font `mtoperatorfont' in version
`normal
'
(Font)                  T1/Merriweather-OsF/m/n --> T1/Merriweather-
OsF/m/n on
input line 2140.
LaTeX Font Info:    Overwriting symbol font `mtoperatorfont' in version
`bold'
(Font)                  T1/Merriweather-OsF/m/n --> T1/Merriweather-
OsF/b/n on
input line 2140.
LaTeX Font Info:    Overwriting math alphabet `\Mathbf' in version
`normal'
(Font)                  T1/Merriweather-OsF/b/n --> T1/Merriweather-
OsF/b/n on
input line 2140.
LaTeX Font Info:    Overwriting math alphabet `\Mathbf' in version `bold'
(Font)                  T1/Merriweather-OsF/b/n --> T1/Merriweather-
OsF/b/n on
input line 2140.
LaTeX Font Info:    Overwriting math alphabet `\Mathit' in version
`normal'
(Font)                  T1/Merriweather-OsF/m/it --> T1/Merriweather-
OsF/m/it o
n input line 2140.
LaTeX Font Info:    Overwriting math alphabet `\Mathit' in version `bold'
(Font)                  T1/Merriweather-OsF/m/it --> T1/Merriweather-
OsF/b/it o
n input line 2140.
LaTeX Font Info:    Overwriting math alphabet `\Mathsf' in version
`normal'
(Font)                  T1/MerriweatherSans-OsF/m/n -->
T1/MerriweatherSans-OsF
/m/n on input line 2140.
LaTeX Font Info:    Overwriting math alphabet `\Mathsf' in version `bold'
(Font)                  T1/MerriweatherSans-OsF/m/n -->
T1/MerriweatherSans-OsF
/b/n on input line 2140.
LaTeX Font Info:    Overwriting math alphabet `\Mathtt' in version
`normal'
(Font)                  T1/lmtt/m/n --> T1/lmtt/m/n on input line 2140.
LaTeX Font Info:    Overwriting math alphabet `\Mathtt' in version `bold'
(Font)                  T1/lmtt/m/n --> T1/lmtt/b/n on input line 2140.
** Latin letters in the normal (resp. bold) math versions are now
** set up to use the fonts T1/Merriweather-OsF/m(b)/it
** Other characters (digits, ...) and \log-like names will be
** typeset with the n shape.
** \hbar
** minus as endash
** \HUGE has been (re)-defined.
** mathastext has declared larger sizes for subscripts.
** To keep LaTeX defaults, use option `defaultmathsizes'.
) (c:/TeXLive/2020/texmf-dist/tex/latex/relsize/relsize.sty
```

```
Package: relsize 2013/03/29 ver 4.1
) (c:/TeXLive/2020/texmf-dist/tex/latex/ragged2e/ragged2e.sty
Package: ragged2e 2019/07/28 v2.2 ragged2e Package (MS)
(c:/TeXLive/2020/texmf-dist/tex/latex/ms/everysel.sty
Package: everysel 2011/10/28 v1.2 EverySelectfont Package (MS)
)
\CenteringLeftskip=\skip50
\RaggedLeftLeftskip=\skip51
\RaggedRightLeftskip=\skip52
\CenteringRightskip=\skip53
\RaggedLeftRightskip=\skip54
\RaggedRightRightskip=\skip55
\CenteringParfillskip=\skip56
\RaggedLeftParfillskip=\skip57
\RaggedRightParfillskip=\skip58
\JustifyingParfillskip=\skip59
\CenteringParindent=\skip60
\RaggedLeftParindent=\skip61
\RaggedRightParindent=\skip62
\JustifyingParindent=\skip63
) (c:/TeXLive/2020/texmf-dist/tex/latex/xcolor/xcolor.sty
Package: xcolor 2016/05/11 v2.12 LaTeX color extensions (UK)
(c:/TeXLive/2020/texmf-dist/tex/latex/graphics-cfg/color.cfg
File: color.cfg 2016/01/02 v1.6 sample color configuration
)
Package xcolor Info: Driver file: pdftex.def on input line 225.
(c:/TeXLive/2020/texmf-dist/tex/latex/graphics-def/pdftex.def
File: pdftex.def 2018/01/08 v1.0l Graphics/color driver for pdftex
)
Package xcolor Info: Model `cmy' substituted by `cmy0' on input line
1348.
Package xcolor Info: Model `hsb' substituted by `rgb' on input line 1352.
Package xcolor Info: Model `RGB' extended on input line 1364.
Package xcolor Info: Model `HTML' substituted by `rgb' on input line
1366.
Package xcolor Info: Model `Hsb' substituted by `hsb' on input line 1367.
Package xcolor Info: Model `tHsb' substituted by `hsb' on input line
1368.
Package xcolor Info: Model `HSB' substituted by `hsb' on input line 1369.
Package xcolor Info: Model `Gray' substituted by `gray' on input line
1370.
Package xcolor Info: Model `wave' substituted by `hsb' on input line
1371.
) (c:/TeXLive/2020/texmf-dist/tex/latex/colortbl/colortbl.sty
Package: colortbl 2020/01/04 v1.0e Color table columns (DPC)
(c:/TeXLive/2020/texmf-dist/tex/latex/tools/array.sty
Package: array 2019/08/31 v2.4l Tabular extension package (FMi)
\col@sep=\dimen136
\ar@mcellbox=\box45
\extrarowheight=\dimen137
\NC@list=\toks25
\extratabsurround=\skip64
\backup@length=\skip65
\ar@cellbox=\box46
```

```
)
\everycr=\toks26
\minrowclearance=\skip66
) (c:/TeXLive/2020/texmf-dist/tex/latex/graphics/graphicx.sty
Package: graphicx 2019/11/30 v1.2a Enhanced LaTeX Graphics (DPC,SPQR)
(c:/TeXLive/2020/texmf-dist/tex/latex/graphics/graphics.sty
Package: graphics 2019/11/30 v1.4a Standard LaTeX Graphics (DPC,SPQR)
(c:/TeXLive/2020/texmf-dist/tex/latex/graphics/trig.sty
Package: trig 2016/01/03 v1.10 sin cos tan (DPC)
) (c:/TeXLive/2020/texmf-dist/tex/latex/graphics-cfg/graphics.cfg
File: graphics.cfg 2016/06/04 v1.11 sample graphics configuration
)
Package graphics Info: Driver file: pdftex.def on input line 105.
)
\Gin@req@height=\dimen138
\Gin@req@width=\dimen139
) (c:/TeXLive/2020/texmf-dist/tex/latex/etoolbox/etoolbox.sty
Package: etoolbox 2019/09/21 v2.5h e-TeX tools for LaTeX (JAW)
\etb@tempcnta=\count178
) (c:/TeXLive/2020/texmf-dist/tex/latex/xpatch/xpatch.sty
(c:/TeXLive/2020/texm
f-dist/tex/latex/l3kernel/expl3.sty
Package: expl3 2020-05-05 L3 programming layer (loader)
(c:/TeXLive/2020/texmf-dist/tex/latex/l3backend/l3backend-pdfmode.def
File: l3backend-pdfmode.def 2020-05-05 L3 backend support: PDF mode
\l__kernel_color_stack_int=\count179
\l__pdf_internal_box=\box47
))
Package: xpatch 2020/03/25 v0.3a Extending etoolbox patching commands
(c:/TeXLive/2020/texmf-dist/tex/latex/l3packages/xparse/xparse.sty
Package: xparse 2020-03-06 L3 Experimental document command parser
\l__xparse_current_arg_int=\count180
\g__xparse_grabber_int=\count181
\l__xparse_m_args_int=\count182
\l__xparse_v_nesting_int=\count183
)) (c:/TeXLive/2020/texmf-dist/tex/latex/environ/environ.sty
Package: environ 2014/05/04 v0.3 A new way to define environments
(c:/TeXLive/2020/texmf-dist/tex/latex/trimspaces/trimspaces.sty
Package: trimspaces 2009/09/17 v1.1 Trim spaces around a token list
)
\@envbody=\toks27
) (c:/TeXLive/2020/texmf-dist/tex/latex/lastpage/lastpage.sty
Package: lastpage 2015/03/29 v1.2m Refers to last page's name (HMM; JPG)
) (c:/TeXLive/2020/texmf-dist/tex/latex/graphics/rotating.sty
Package: rotating 2016/08/11 v2.16d rotated objects in LaTeX
(c:/TeXLive/2020/texmf-dist/tex/latex/base/ifthen.sty
Package: ifthen 2014/09/29 v1.1c Standard LaTeX ifthen package (DPC)
)
\c@r@tfl@t=\count184
\rotFPtop=\skip67
\rotFPbot=\skip68
\rot@float@box=\box48
\rot@mess@toks=\toks28
) (c:/TeXLive/2020/texmf-dist/tex/latex/graphics/lscape.sty
```

```
Package: lscape 2000/10/22 v3.01 Landscape Pages (DPC)
) (c:/TeXLive/2020/texmf-dist/tex/latex/tools/afterpage.sty
Package: afterpage 2014/10/28 v1.08 After-Page Package (DPC)
\AP@output=\toks29
\AP@partial=\box49
\AP@footins=\box50
) (c:/TeXLive/2020/texmf-dist/tex/latex/textpos/textpos.sty
Package: textpos 2019/04/15 v1.9.1
Package: textpos 2019/04/15 1.9.1, absolute positioning of text on the
page
(c:/TeXLive/2020/texmf-dist/tex/latex/ms/everyshi.sty
Package: everyshi 2001/05/15 v3.00 EveryShipout Package (MS)
)
\TP@textbox=\box51
\TP@holdbox=\box52
\TPHorizModule=\dimen140
\TPVertModule=\dimen141
\TP@margin=\dimen142
\TP@absmargin=\dimen143
Grid set 16 x 16 = 37.34424pt x 52.81541pt
\TPboxrulesize=\dimen144
\TP@ox=\dimen145
\TP@oy=\dimen146
\TP@tbargs=\toks30
\TP@prevdepth=\dimen147
TextBlockOrigin set to 0pt x 0pt
) (c:/TeXLive/2020/texmf-dist/tex/latex/url/url.sty
\Urlmuskip=\muskip19
Package: url 2013/09/16  ver 3.4  Verb mode for urls, etc.
) (c:/TeXLive/2020/texmf-dist/tex/latex/newfloat/newfloat.sty
Package: newfloat 2019/09/02 v1.1l Defining new floating environments
(AR)
Package newfloat Info: `rotating' package detected.
) (c:/TeXLive/2020/texmf-dist/tex/latex/mdframed/mdframed.sty
Package: mdframed 2013/07/01 1.9b: mdframed
(c:/TeXLive/2020/texmf-dist/tex/latex/kvoptions/kvoptions.sty
Package: kvoptions 2019/11/29 v3.13 Key value format for package options
(HO)
(c:/TeXLive/2020/texmf-dist/tex/generic/ltxcmds/ltxcmds.sty
Package: ltxcmds 2019/12/15 v1.24 LaTeX kernel commands for general use
(HO)
) (c:/TeXLive/2020/texmf-dist/tex/generic/kvsetkeys/kvsetkeys.sty
Package: kvsetkeys 2019/12/15 v1.18 Key value parser (HO)
)) (c:/TeXLive/2020/texmf-dist/tex/latex/zref/zref-abspage.sty
Package: zref-abspage 2020-03-03 v2.29 Module abspage for zref (HO)
(c:/TeXLive/2020/texmf-dist/tex/latex/zref/zref-base.sty
Package: zref-base 2020-03-03 v2.29 Module base for zref (HO)
(c:/TeXLive/2020/texmf-dist/tex/generic/infwarerr/infwarerr.sty
Package: infwarerr 2019/12/03 v1.5 Providing info/warning/error messages
(HO)
) (c:/TeXLive/2020/texmf-dist/tex/generic/kvdefinekeys/kvdefinekeys.sty
Package: kvdefinekeys 2019-12-19 v1.6 Define keys (HO)
) (c:/TeXLive/2020/texmf-dist/tex/latex/pdftexcmds/pdftexcmds.sty
```

```
Package: pdftexcmds 2019/11/24 v0.31 Utility functions of pdfTeX for
LuaTeX (HO
)
Package pdftexcmds Info: \pdf@primitive is available.
Package pdftexcmds Info: \pdf@ifprimitive is available.
Package pdftexcmds Info: \pdfdraftmode found.
) (c:/TeXLive/2020/texmf-dist/tex/generic/etexcmds/etexcmds.sty
Package: etexcmds 2019/12/15 v1.7 Avoid name clashes with e-TeX commands
(HO)
) (c:/TeXLive/2020/texmf-dist/tex/latex/auxhook/auxhook.sty
Package: auxhook 2019-12-17 v1.6 Hooks for auxiliary files (HO)
)
Package zref Info: New property list: main on input line 763.
Package zref Info: New property: default on input line 764.
Package zref Info: New property: page on input line 765.
) (c:/TeXLive/2020/texmf-dist/tex/generic/atbegshi/atbegshi.sty
Package: atbegshi 2019/12/05 v1.19 At begin shipout hook (HO)
)
\c@abspage=\count185
Package zref Info: New property: abspage on input line 66.
) (c:/TeXLive/2020/texmf-dist/tex/latex/needspace/needspace.sty
Package: needspace 2010/09/12 v1.3d reserve vertical space
)
\mdf@templength=\skip69
\c@mdf@globalstyle@cnt=\count186
\mdf@skipabove@length=\skip70
\mdf@skipbelow@length=\skip71
\mdf@leftmargin@length=\skip72
\mdf@rightmargin@length=\skip73
\mdf@innerleftmargin@length=\skip74
\mdf@innerrightmargin@length=\skip75
\mdf@innertopmargin@length=\skip76
\mdf@innerbottommargin@length=\skip77
\mdf@splittopskip@length=\skip78
\mdf@splitbottomskip@length=\skip79
\mdf@outermargin@length=\skip80
\mdf@innermargin@length=\skip81
\mdf@linewidth@length=\skip82
\mdf@innerlinewidth@length=\skip83
\mdf@middlelinewidth@length=\skip84
\mdf@outerlinewidth@length=\skip85
\mdf@roundcorner@length=\skip86
\mdf@footenotedistance@length=\skip87
\mdf@userdefinedwidth@length=\skip88
\mdf@needspace@length=\skip89
\mdf@frametitleaboveskip@length=\skip90
\mdf@frametitlebelowskip@length=\skip91
\mdf@frametitlerulewidth@length=\skip92
\mdf@frametitleleftmargin@length=\skip93
\mdf@frametitlerightmargin@length=\skip94
\mdf@shadowsize@length=\skip95
\mdf@extratopheight@length=\skip96
\mdf@subtitleabovelinewidth@length=\skip97
\mdf@subtitlebelowlinewidth@length=\skip98
```

```
\mdf@subtitleaboveskip@length=\skip99
\mdf@subtitlebelowskip@length=\skip100
\mdf@subtitleinneraboveskip@length=\skip101
\mdf@subtitleinnerbelowskip@length=\skip102
\mdf@subsubtitleabovelinewidth@length=\skip103
\mdf@subsubtitlebelowlinewidth@length=\skip104
\mdf@subsubtitleaboveskip@length=\skip105
\mdf@subsubtitlebelowskip@length=\skip106
\mdf@subsubtitleinneraboveskip@length=\skip107
\mdf@subsubtitleinnerbelowskip@length=\skip108
(c:/TeXLive/2020/texmf-dist/tex/latex/mdframed/md-frame-0.mdf
File: md-frame-0.mdf 2013/07/01\ 1.9b: md-frame-0
)
\mdf@frametitlebox=\box53
\mdf@footnotebox=\box54
\mdf@splitbox@one=\box55
\mdf@splitbox@two=\box56
\mdf@splitbox@save=\box57
\mdfsplitboxwidth=\skip109
\mdfsplitboxtotalwidth=\skip110
\mdfsplitboxheight=\skip111
\mdfsplitboxdepth=\skip112
\mdfsplitboxtotalheight=\skip113
\mdfframetitleboxwidth=\skip114
\mdfframetitleboxtotalwidth=\skip115
\mdfframetitleboxheight=\skip116
\mdfframetitleboxdepth=\skip117
\mdfframetitleboxtotalheight=\skip118
\mdffootnoteboxwidth=\skip119
\mdffootnoteboxtotalwidth=\skip120
\mdffootnoteboxheight=\skip121
\mdffootnoteboxdepth=\skip122
\mdffootnoteboxtotalheight=\skip123
\mdftotallinewidth=\skip124
\mdfboundingboxwidth=\skip125
\mdfboundingboxtotalwidth=\skip126
\mdfboundingboxheight=\skip127
\mdfboundingboxdepth=\skip128
\mdfboundingboxtotalheight=\skip129
\mdf@freevspace@length=\skip130
\mdf@horizontalwidthofbox@length=\skip131
\mdf@verticalmarginwhole@length=\skip132
\mdf@horizontalspaceofbox=\skip133
\mdfsubtitleheight=\skip134
\mdfsubsubtitleheight=\skip135
\c@mdfcountframes=\count187

****** mdframed patching \endmdf@trivlist

****** -- success******

\mdf@envdepth=\count188
\c@mdf@env@i=\count189
\c@mdf@env@ii=\count190
```

```
\c@mdf@zref@counter=\count191
Package zref Info: New property: mdf@pagevalue on input line 895.
) (c:/TeXLive/2020/texmf-dist/tex/latex/titlesec/titlesec.sty
Package: titlesec 2019/10/16 v2.13 Sectioning titles
\ttl@box=\box58
\beforetitleunit=\skip136
\aftertitleunit=\skip137
\ttl@plus=\dimen148
\ttl@minus=\dimen149
\ttl@toksa=\toks31
\titlewidth=\dimen150
\titlewidthlast=\dimen151
\titlewidthfirst=\dimen152
) (c:/TeXLive/2020/texmf-dist/tex/latex/koma-script/scrextend.sty
Package: scrextend 2020/04/19 v3.30 KOMA-Script package (extend other
classes w
ith features of KOMA-Script classes)
(c:/TeXLive/2020/texmf-dist/tex/latex/koma-script/scrkbase.sty
Package: scrkbase 2020/04/19 v3.30 KOMA-Script package (KOMA-Script-
dependent b
asics and keyval usage)
(c:/TeXLive/2020/texmf-dist/tex/latex/koma-script/scrbase.sty
Package: scrbase 2020/04/19 v3.30 KOMA-Script package (KOMA-Script-
independent
basics and keyval usage)
(c:/TeXLive/2020/texmf-dist/tex/latex/koma-script/scrlfile.sty
Package: scrlfile 2020/04/19 v3.30 KOMA-Script package (loading files)
)))
Package scrextend Info: unexpected definition of `\@makefnmark'.
(scrextend)             Trying to patch it on input line 1589.
Package scrextend Info: patch seems to be successfull on input line 1589.
)

LaTeX Font Warning: Font shape `T1/cmr/m/n' in size <7.5> not available
(Font)              size <7> substituted on input line 65.

(c:/TeXLive/2020/texmf-dist/tex/latex/tools/calc.sty
Package: calc 2017/05/25 v4.3 Infix arithmetic (KKT,FJ)
\calc@Acount=\count192
\calc@Bcount=\count193
\calc@Adimen=\dimen153
\calc@Bdimen=\dimen154
\calc@Askip=\skip138
\calc@Bskip=\skip139
LaTeX Info: Redefining \setlength on input line 80.
LaTeX Info: Redefining \addtolength on input line 81.
\calc@Ccount=\count194
\calc@Cskip=\skip140
) (c:/TeXLive/2020/texmf-dist/tex/latex/geometry/geometry.sty
Package: geometry 2020/01/02 v5.9 Page Geometry
(c:/TeXLive/2020/texmf-dist/tex/generic/iftex/ifvtex.sty
Package: ifvtex 2019/10/25 v1.7 ifvtex legacy package. Use iftex instead.
)
\Gm@cnth=\count195
```

```
\Gm@cntv=\count196
\c@Gm@tempcnt=\count197
\Gm@bindingoffset=\dimen155
\Gm@wd@mp=\dimen156
\Gm@odd@mp=\dimen157
\Gm@even@mp=\dimen158
\Gm@layoutwidth=\dimen159
\Gm@layoutheight=\dimen160
\Gm@layouthoffset=\dimen161
\Gm@layoutvoffset=\dimen162
\Gm@dimlist=\toks32
) (c:/TeXLive/2020/texmf-dist/tex/latex/hyperref/hyperref.sty
Package: hyperref 2020/01/14 v7.00d Hypertext links for LaTeX
(c:/TeXLive/2020/texmf-dist/tex/generic/pdfescape/pdfescape.sty
Package: pdfescape 2019/12/09 v1.15 Implements pdfTeX's escape features
(HO)
) (c:/TeXLive/2020/texmf-dist/tex/latex/hycolor/hycolor.sty
Package: hycolor 2020-01-27 v1.10 Color options for hyperref/bookmark
(HO)
) (c:/TeXLive/2020/texmf-dist/tex/latex/letltxmacro/letltxmacro.sty
Package: letltxmacro 2019/12/03 v1.6 Let assignment for LaTeX macros (HO)
)
\@linkdim=\dimen163
\Hy@linkcounter=\count198
\Hy@pagecounter=\count199
(c:/TeXLive/2020/texmf-dist/tex/latex/hyperref/pd1enc.def
File: pd1enc.def 2020/01/14 v7.00d Hyperref: PDFDocEncoding definition
(HO)
Now handling font encoding PD1 ...
... no UTF-8 mapping file for font encoding PD1
) (c:/TeXLive/2020/texmf-dist/tex/generic/intcalc/intcalc.sty
Package: intcalc 2019/12/15 v1.3 Expandable calculations with integers
(HO)
)
\Hy@SavedSpaceFactor=\count266
Package hyperref Info: Option `colorlinks' set `true' on input line 4421.
Package hyperref Info: Hyper figures OFF on input line 4547.
Package hyperref Info: Link nesting OFF on input line 4552.
Package hyperref Info: Hyper index ON on input line 4555.
Package hyperref Info: Plain pages OFF on input line 4562.
Package hyperref Info: Backreferencing OFF on input line 4567.
Package hyperref Info: Implicit mode ON; LaTeX internals redefined.
Package hyperref Info: Bookmarks ON on input line 4800.
\c@Hy@tempcnt=\count267
LaTeX Info: Redefining \url on input line 5159.
\XeTeXLinkMargin=\dimen164
(c:/TeXLive/2020/texmf-dist/tex/generic/bitset/bitset.sty
Package: bitset 2019/12/09 v1.3 Handle bit-vector datatype (HO)
(c:/TeXLive/2020/texmf-dist/tex/generic/bigintcalc/bigintcalc.sty
Package: bigintcalc 2019/12/15 v1.5 Expandable calculations on big
integers (HO
)
))
\Fld@menulength=\count268
```

```
\Field@Width=\dimen165
\Fld@charsize=\dimen166
Package hyperref Info: Hyper figures OFF on input line 6430.
Package hyperref Info: Link nesting OFF on input line 6435.
Package hyperref Info: Hyper index ON on input line 6438.
Package hyperref Info: backreferencing OFF on input line 6445.
Package hyperref Info: Link coloring ON on input line 6448.
Package hyperref Info: Link coloring with OCG OFF on input line 6455.
Package hyperref Info: PDF/A mode OFF on input line 6460.
LaTeX Info: Redefining \ref on input line 6500.
LaTeX Info: Redefining \pageref on input line 6504.
\Hy@abspage=\count269
\c@Item=\count270
\c@Hfootnote=\count271
)
Package hyperref Info: Driver (autodetected): hpdftex.
(c:/TeXLive/2020/texmf-dist/tex/latex/hyperref/hpdftex.def
File: hpdftex.def 2020/01/14 v7.00d Hyperref driver for pdfTeX
(c:/TeXLive/2020/texmf-dist/tex/latex/atveryend/atveryend.sty
Package: atveryend 2019-12-11 v1.11 Hooks at the very end of document
(HO)
)
\HyAnn@Count=\count272
\Fld@listcount=\count273
\c@bookmark@seq@number=\count274
(c:/TeXLive/2020/texmf-dist/tex/latex/rerunfilecheck/rerunfilecheck.sty
Package: rerunfilecheck 2019/12/05 v1.9 Rerun checks for auxiliary files
(HO)
(c:/TeXLive/2020/texmf-dist/tex/generic/uniquecounter/uniquecounter.sty
Package: uniquecounter 2019/12/15 v1.4 Provide unlimited unique counter
(HO)
)
Package uniquecounter Info: New unique counter `rerunfilecheck' on input
line 2
86.
)
\Hy@SectionHShift=\skip141
) (c:/TeXLive/2020/texmf-dist/tex/latex/preprint/authblk.sty
Package: authblk 2001/02/27 1.3 (PWD)
\affilsep=\skip142
\@affilsep=\skip143
\c@Maxaffil=\count275
\c@authors=\count276
\c@affil=\count277
) (c:/TeXLive/2020/texmf-dist/tex/latex/footmisc/footmisc.sty
Package: footmisc 2011/06/06 v5.5b a miscellany of footnote facilities
\FN@temptoken=\toks33
\footnotemargin=\dimen167
\c@pp@next@reset=\count278
Package footmisc Info: Declaring symbol style bringhurst on input line
855.
Package footmisc Info: Declaring symbol style chicago on input line 863.
Package footmisc Info: Declaring symbol style wiley on input line 872.
```

```
Package footmisc Info: Declaring symbol style lamport-robust on input
line 883.

Package footmisc Info: Declaring symbol style lamport* on input line 903.
Package footmisc Info: Declaring symbol style lamport*-robust on input
line 924
.
) (c:/TeXLive/2020/texmf-dist/tex/latex/fancyhdr/fancyhdr.sty
Package: fancyhdr 2019/01/31 v3.10 Extensive control of page headers and
footer
s
\f@nch@headwidth=\skip144
\f@nch@O@elh=\skip145
\f@nch@O@erh=\skip146
\f@nch@O@olh=\skip147
\f@nch@O@orh=\skip148
\f@nch@O@elf=\skip149
\f@nch@O@erf=\skip150
\f@nch@O@olf=\skip151
\f@nch@O@orf=\skip152
) (c:/TeXLive/2020/texmf-dist/tex/generic/alphalph/alphalph.sty
Package: alphalph 2019/12/09 v2.6 Convert numbers to letters (HO)
)
\c@authorfn=\count279
(c:/TeXLive/2020/texmf-dist/tex/latex/abstract/abstract.sty
Package: abstract 2009/06/08 v1.2a configurable abstracts
\abstitleskip=\skip153
\absleftindent=\skip154
\absrightindent=\skip155
\absparindent=\skip156
\absparsep=\skip157
)
Package newfloat Info: New float `keypoints' with options
`placement=t!,name=kp
t' on input line 286.
\c@keypoints=\count280
\newfloat@ftype=\count281
Package newfloat Info: float type `keypoints'=8 on input line 286.
(c:/TeXLive/2020/texmf-dist/tex/latex/enumitem/enumitem.sty
Package: enumitem 2019/06/20 v3.9 Customized lists
\labelindent=\skip158
\enit@outerparindent=\dimen168
\enit@toks=\toks34
\enit@inbox=\box59
\enit@count@id=\count282
\enitdp@description=\count283
) (c:/TeXLive/2020/texmf-dist/tex/latex/quoting/quoting.sty
Package: quoting 2014/01/28 v0.1c Consolidated environment for displayed
text
\quo@toppartop=\skip159
) (c:/TeXLive/2020/texmf-dist/tex/latex/sttools/stfloats.sty
Package: stfloats 2017/03/27 v3.3 Improve float mechanism and
baselineskip sett
ings
```

```
\@dblbotnum=\count284
\c@dblbotnumber=\count285
) (c:/TeXLive/2020/texmf-dist/tex/latex/booktabs/booktabs.sty
Package: booktabs 2020/01/12 v1.61803398 Publication quality tables
\heavyrulewidth=\dimen169
\lightrulewidth=\dimen170
\cmidrulewidth=\dimen171
\belowrulesep=\dimen172
\belowbottomsep=\dimen173
\aboverulesep=\dimen174
\abovetopsep=\dimen175
\cmidrulesep=\dimen176
\cmidrulekern=\dimen177
\defaultaddspace=\dimen178
\@cmidla=\count286
\@cmidlb=\count287
\@aboverulesep=\dimen179
\@belowrulesep=\dimen180
\@thisruleclass=\count288
\@lastruleclass=\count289
\@thisrulewidth=\dimen181
) (c:/TeXLive/2020/texmf-dist/tex/latex/tools/tabularx.sty
Package: tabularx 2020/01/15 v2.11c `tabularx' package (DPC)
\TX@col@width=\dimen182
\TX@old@table=\dimen183
\TX@old@col=\dimen184
\TX@target=\dimen185
\TX@delta=\dimen186
\TX@cols=\count290
\TX@ftn=\toks35
)
\enitdp@tablenotes=\count291
(c:/TeXLive/2020/texmf-dist/tex/latex/caption/caption.sty
Package: caption 2020/01/03 v3.4h Customizing captions (AR)
(c:/TeXLive/2020/texmf-dist/tex/latex/caption/caption3.sty
Package: caption3 2020/01/03 v1.8h caption3 kernel (AR)
Package caption3 Info: TeX engine: e-TeX on input line 61.
\captionmargin=\dimen187
\captionmargin@=\dimen188
\captionwidth=\dimen189
\caption@tempdima=\dimen190
\caption@indent=\dimen191
\caption@parindent=\dimen192
\caption@hangindent=\dimen193
Package caption Info: Standard document class detected.
)
\c@caption@flags=\count292
\c@continuedfloat=\count293
Package caption Info: hyperref package is loaded.
Package caption Info: rotating package is loaded.
) (c:/TeXLive/2020/texmf-dist/tex/latex/natbib/natbib.sty
Package: natbib 2010/09/13 8.31b (PWD, AO)
\bibhang=\skip160
\bibsep=\skip161
```

```
LaTeX Info: Redefining \cite on input line 694.
\c@NAT@ctr=\count294
)) (c:/TeXLive/2020/texmf-dist/tex/latex/siunitx/siunitx.sty
Package: siunitx 2020/02/25 v2.8b A comprehensive (SI) units package
(c:/TeXLive/2020/texmf-dist/tex/latex/amsmath/amstext.sty
Package: amstext 2000/06/29 v2.01 AMS text
(c:/TeXLive/2020/texmf-dist/tex/latex/amsmath/amsgen.sty
File: amsgen.sty 1999/11/30 v2.0 generic functions
\@emptytoks=\toks36
\ex@=\dimen194
)) (c:/TeXLive/2020/texmf-dist/tex/latex/l3packages/l3keys2e/l3keys2e.sty
Package: l3keys2e 2020-03-06 LaTeX2e option processing using LaTeX3 keys
)
\l__siunitx_tmp_box=\box60
\l__siunitx_tmp_dim=\dimen195
\l__siunitx_tmp_int=\count295
\l__siunitx_number_mantissa_length_int=\count296
\l__siunitx_number_uncert_length_int=\count297
\l__siunitx_round_int=\count298
\l__siunitx_process_decimal_int=\count299
\l__siunitx_process_uncertainty_int=\count300
\l__siunitx_process_fixed_int=\count301
\l__siunitx_process_integer_min_int=\count302
\l__siunitx_process_precision_int=\count303
\l__siunitx_group_min_int=\count304
\l__siunitx_angle_marker_box=\box61
\l__siunitx_angle_unit_box=\box62
\l__siunitx_angle_marker_dim=\dimen196
\l__siunitx_angle_unit_dim=\dimen197
\l__siunitx_unit_int=\count305
\l__siunitx_unit_denominator_int=\count306
\l__siunitx_unit_numerator_int=\count307
\l__siunitx_unit_prefix_int=\count308
\l__siunitx_unit_prefix_base_int=\count309
\l__siunitx_unit_prefix_gram_int=\count310
\l__siunitx_number_product_int=\count311
\c__siunitx_one_fill_skip=\skip162
\l__siunitx_table_unit_align_skip=\skip163
\l__siunitx_table_exponent_dim=\dimen198
\l__siunitx_table_integer_dim=\dimen199
\l__siunitx_table_mantissa_dim=\dimen256
\l__siunitx_table_marker_dim=\dimen257
\l__siunitx_table_result_dim=\dimen258
\l__siunitx_table_uncert_dim=\dimen259
\l__siunitx_table_fill_pre_dim=\dimen260
\l__siunitx_table_fill_post_dim=\dimen261
\l__siunitx_table_fill_mid_dim=\dimen262
\l__siunitx_table_pre_box=\box63
\l__siunitx_table_post_box=\box64
\l__siunitx_table_mantissa_box=\box65
\l__siunitx_table_result_box=\box66
\l__siunitx_table_number_align_skip=\skip164
\l__siunitx_table_text_align_skip=\skip165
(c:/TeXLive/2020/texmf-dist/tex/latex/translator/translator.sty
```

```
Package: translator 2019-05-31 v1.12a Easy translation of strings in
LaTeX
)) (c:/TeXLive/2020/texmf-dist/tex/latex/svg/svg.sty
Package: svg 2020/05/07 v2.02f (include SVG pictures)
(c:/TeXLive/2020/texmf-dist/tex/latex/tools/shellesc.sty
Package: shellesc 2019/11/08 v1.0c unified shell escape interface for
LaTeX
Package shellesc Info: Restricted shell escape enabled on input line 77.
)
\svg@box=\box67
\c@svg@param@lastpage=\count312
\c@svg@param@currpage=\count313
) (c:/TeXLive/2020/texmf-dist/tex/latex/transparent/transparent.sty
Package: transparent 2019/11/29 v1.4 Transparency via pdfTeX's color
stack (HO)

) (c:/TeXLive/2020/texmf-dist/tex/latex/amsmath/amsmath.sty
Package: amsmath 2020/01/20 v2.17e AMS math features
\@mathmargin=\skip166
For additional information on amsmath, use the `?' option.
(c:/TeXLive/2020/texmf-dist/tex/latex/amsmath/amsbsy.sty
Package: amsbsy 1999/11/29 v1.2d Bold Symbols
\pmbraise@=\dimen263
) (c:/TeXLive/2020/texmf-dist/tex/latex/amsmath/amsopn.sty
Package: amsopn 2016/03/08 v2.02 operator names
)
\inf@bad=\count314
LaTeX Info: Redefining \frac on input line 227.
\uproot@=\count315
\leftroot@=\count316
LaTeX Info: Redefining \overline on input line 389.
\classnum@=\count317
\DOTSCASE@=\count318
LaTeX Info: Redefining \ldots on input line 486.
LaTeX Info: Redefining \dots on input line 489.
LaTeX Info: Redefining \cdots on input line 610.
\Mathstrutbox@=\box68
\strutbox@=\box69
\big@size=\dimen264
LaTeX Font Info:    Redeclaring font encoding OML on input line 733.
LaTeX Font Info:    Redeclaring font encoding OMS on input line 734.
\macc@depth=\count319
\c@MaxMatrixCols=\count320
\dotsspace@=\muskip20
\c@parentequation=\count321
\dspbrk@lvl=\count322
\tag@help=\toks37
\row@=\count323
\column@=\count324
\maxfields@=\count325
\andhelp@=\toks38
\eqnshift@=\dimen265
\alignsep@=\dimen266
\tagshift@=\dimen267
```

```
\tagwidth@=\dimen268
\totwidth@=\dimen269
\lineht@=\dimen270
\@envbody=\toks39
\multlinegap=\skip167
\multlinetaggap=\skip168
\mathdisplay@stack=\toks40
LaTeX Info: Redefining \[ on input line 2859.
LaTeX Info: Redefining \] on input line 2860.
) (c:/TeXLive/2020/texmf-dist/tex/latex/academicons/academicons.sty
Package: academicons 2018/06/27 v1.8.6-2 Academicons Icons
)

! LaTeX Error: File `orcidlink.sty' not found.

Type X to quit or <RETURN> to proceed,
or enter new name. (Default extension: sty)

Enter file name:
! Emergency stop.
<read *>

l.24 ^^M

*** (cannot \read from terminal in nonstop modes)


Here is how much of TeX's memory you used:
 17062 strings out of 480681
 304451 string characters out of 5908536
 666796 words of memory out of 5000000
 32595 multiletter control sequences out of 15000+600000
 533869 words of font info for 26 fonts, out of 8000000 for 9000
 1141 hyphenation exceptions out of 8191
 65i,0n,110p,794b,142s stack positions out of
5000i,500n,10000p,200000b,80000s
!  ==> Fatal error occurred, no output PDF file produced!
```

Click here to access/download;LaTeX - Other (clo, cls, sty etc.);example-grid-100x100pt.png

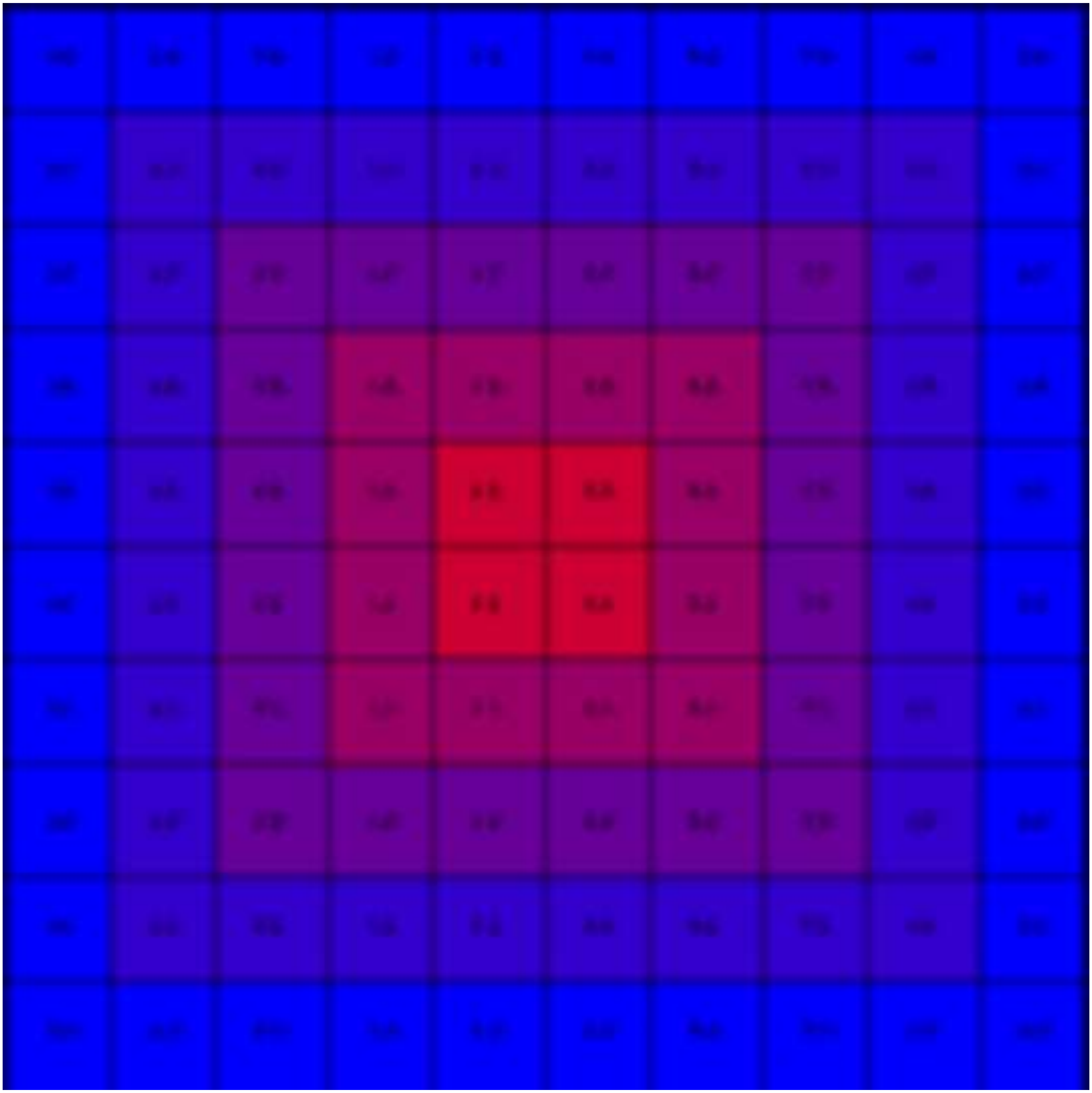

Click here to access/download;LaTeX - Other (clo, cls, sty etc.);example-image.png

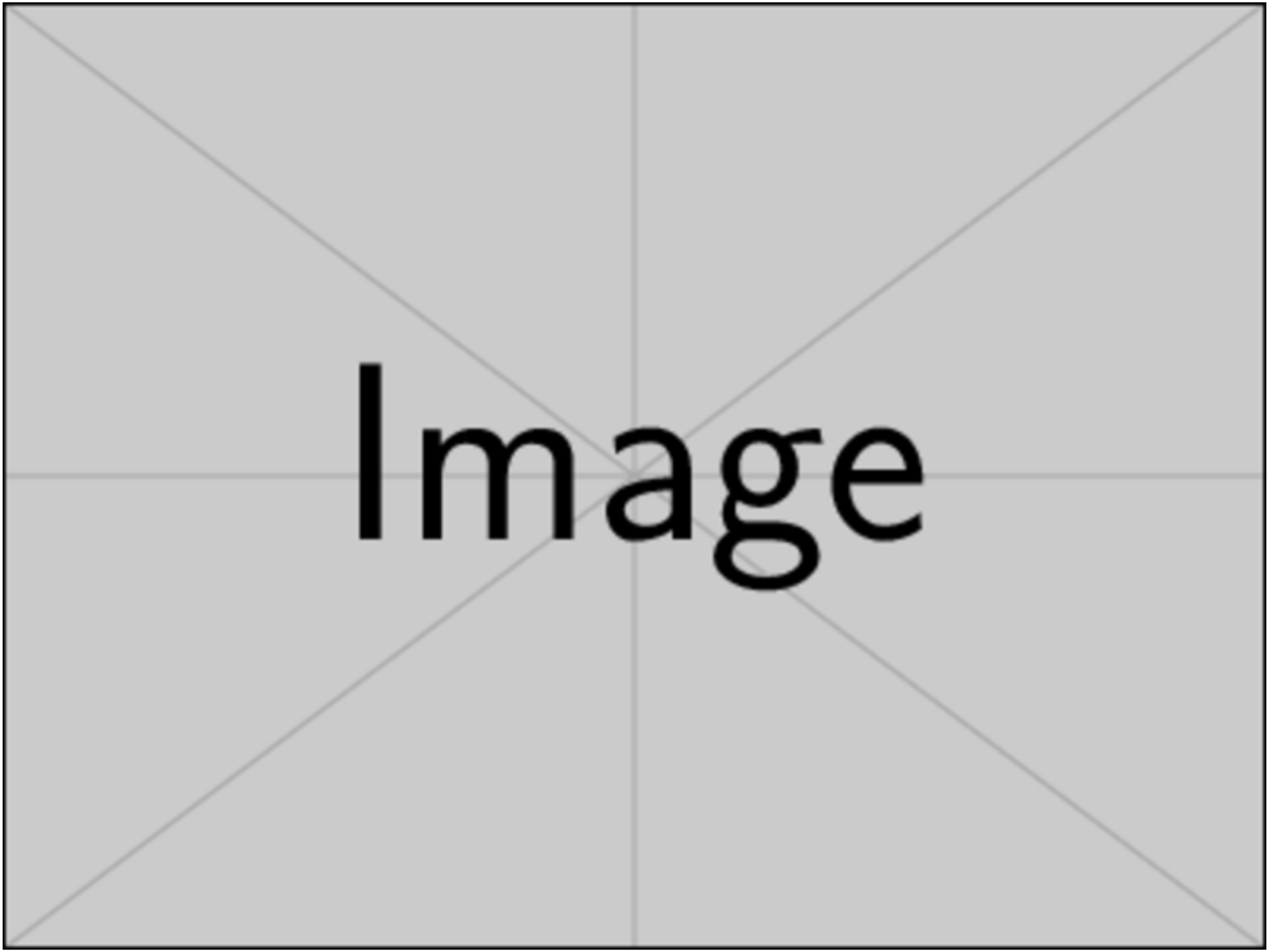

Click here

C
li

OXFORD

latex main figure
Click here to access/download;LaTeX - Figure (eps, ps, etc.);ap11.png

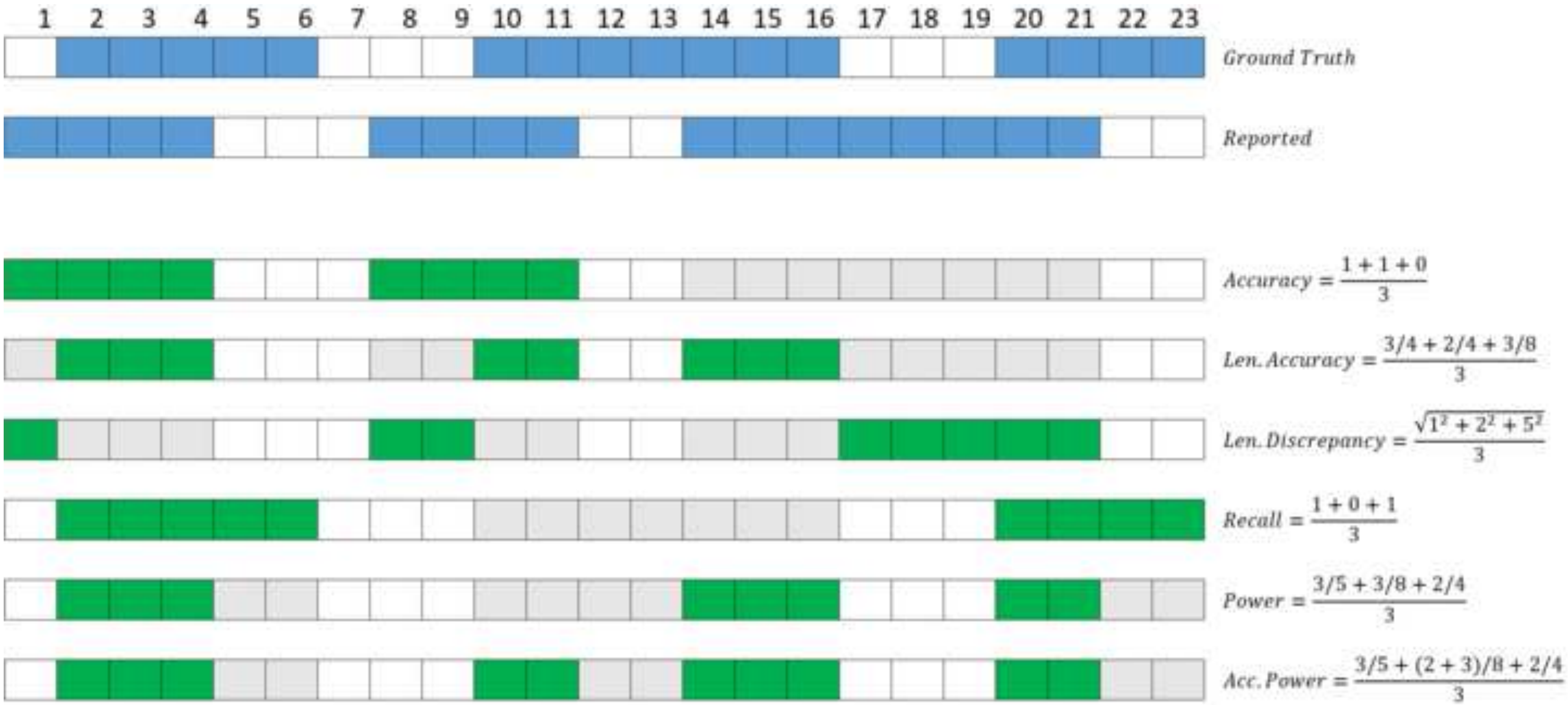

Manuscript

Click here to access/download;Manuscript;IBD_Benckmark_GigSci_R2-

OXFORD

(GIGA)^n SCIENCE

GigaScience, 2017, 1–10

doi: xx.xxxx/xxxx
Manuscript in Preparation
Paper

PAPER

Open-source benchmarking of IBD segment detection methods for biobank-scale cohorts

Kecong Tang [1], Ardalan Naseri [2], Yuan Wei [1], Shaojie Zhang [1,*] and Degui Zhi [2,*]

[1]Department of Computer Science, University of Central Florida, Orlando, FL,32816, USA and [2]School of Biomedical Informatics, The University of Texas Health Science Center at Houston, Houston, TX 77030, USA

*Shaojie.Zhang@ucf.edu; Degui.Zhi@uth.tmc.edu

Abstract

In the recent biobank era of genetics, the problem of Identical-By-Descent (IBD) segment detection received renewed interest, as IBD segments in large cohorts offer unprecedented opportunities in the study of population and genealogical history, as well as genetic association of long haplotypes. While a new generation of efficient methods for IBD segment detection become available, direct comparison of these methods is difficult: existing benchmarks were often evaluated in different datasets, some are not openly accessible; methods benchmarked were run under sub-optimal parameters; benchmark performance metrics were not defined consistently. Here, we developed a comprehensive and completely open-source evaluation of the power, accuracy, and resource consumption of these IBD segment detection methods using realistic population genetic simulations with various settings. Our results pave the road for fair evaluation of IBD segment detection methods and provide an practical guide for users.

Key words: identical-by-descent; biobank-scale data; IBD segment detection tools; benchmarking

Introduction

Identical-By-Descent (IBD) segments, i.e. DNA segments inherited from a common ancestor [1, 2] provide direct evidence of genetic relatedness. It plays a key role in population genetic research. Given whole-genome genetic data of a cohort of individuals, IBD segments could be used to detect relations among them [3, 4]. IBD segments are used in association analysis and can also be directly used in IBD mapping to detect signals of disease-causing markers in population samples [5, 6, 7, 8, 9]. IBD segments can also be used to estimate missing genotypes from the haplotype or genotype reference panels [10]. IBD segments have also been used to phase genotype data [11, 12]. Direct to consumer (DTC) genetic testing companies use IBD segments to offer services of inferred family history [13]. IBD segments may help to identify individuals without direct access to their genetic data in forensic settings [14].

Several IBD segment detection tools have been developed in the last decade. The early generation of tools were designed to detect IBD segments among hundreds to thousands of individuals from a genotype or a haplotype panel [15, 16, 1, 17]. In the biobank era, with the need for processing hundreds of thousands or even millions of haplotypes, a new generation of efficient IBD segment detection tools have been designed in the past few years [18, 19, 20, 21, 22].

While all these methods claim to be efficient and accurate, a direct and reproducible comparison of these methods is still missing. Each of the methods uses a unique solution to solve the IBD detection problem. As a result, some of the proposed methods may be more suitable for certain settings while it may fail in some other cases. For example, one method may not work well in the presence of high genotyping errors. Moreover, the efficiency of some approaches could be more significant in very large data.

Therefore, in this work, we aim to systematically benchmark the new generation of IBD segment detection methods. To facilitate transparency, reproducibility, and convenience, we choose to leverage the latest advanced population genetics simulation tools. For the quality assessment of the IBD call, we define several metrics. The metrics aim to evaluate the accuracy of IBD calls and the detection power of the tools. We define three accuracy and three power metrics considering coverage and length in both single segment and multiple segments upon three sets of data. Moreover, we evalu-

Compiled on: October 18, 2022.
Draft manuscript prepared by the author.

ate the tools regarding the run time and memory consumption. We measure run times and memory usages with the same abundant resources for all tools with increasing sizes of data input.

Methods

When ground truth IBD segments in real data are available, real data is always preferred. However, benchmarking using real data is limited to only close relatives, and mostly for long IBD segments. With advanced population genetics simulation tools, we can have precise ground truth IBD segments among the whole simulated population. Coalescent simulators have been used to evaluate the IBD calls previously [19, 20, 18]. Here, we also use the coalescent simulation tool msprime [23] to simulate the datasets. To benchmark the power and accuracy of the IBD detection tools, we used simulated data. On the other hand, for the run time and memory usage, we used UK Biobank data [24]. We used the phased haplotype panels to input the IBD segment detection tools for all of our benchmarks.

Simulated datasets

We used msprime v1.0.1 to generate three population data sets: East Asian (EAS), European (EUR), and African (AFR). Additionally we generated a mixed set of EAS, EUR, and AFR. These data sets contain the chromosome 20 sequences of 4,000 individuals (8,000 haplotypes), based on the out-of-Africa population model [25]. We used HapMap phase II GRCh37 [26] as the recombination map and $1.38\text{x}10^{-8}$ as the mutation rate. The true IBD segments are determined as the contiguous segment among the tree sequence generated by msprime, where the haplotype pairs share the same most recent common ancestor (MRCA). We sampled the trees for every 5,000 base pair physical distance, and the true IBD segments were extracted if their genetic lengths are at least 1 centiMorgan (cM). Once the data sets were simulated, we filter out the sites having multiple allele values and singletons.

Then we generated array density data sets by down sampling the original sequencing data sets. To achieve an even marker density, a common practice in marker design, each sequencing panel was first given a target number of markers, we used the number 17,197 same as the number of markers in UK Biobank chromosome 20. Then a centiMorgan interval (I) was calculated from dividing the total genetic length of the chromosome by the desired number of sites, this (I) indicated the ideal distance between two markers. As mentioned, in many cases there may not be any marker in many continuous (I), a window size (w) was considered by attempting to take w markers from a *w by I* range. After that, our program tried to select each marker from its original (I) first, then tried to take a marker from the leftover markers in the *w by I* range if there was not any marker in the original (I). During the marker selection, a site with the highest minor allele frequency (MAF) would be selected. Acquiring perfect even marker density and reaching desired number of markers are two goals with conflict. After multiple runs with different window sizes, we chose 5 as the window size, at the end, we had 15,958 sites in the EAS array data, 15,546 sites in the EUR array data, 15,313 sites in the AFR array data, and 16,013 sites in the mixed array data.

To simulate genotyping errors, we randomly implanted genotyping errors with the rates of 0.1%, 0.2%, 0.3%, and 0.4% per genotype over the variant sites. Although this is a simplification of the realistic error profiles in sequencing data (e.g., false positively called non-variant sites were not included, nucleotide-specific and region-specific error profiles were not modeled), we did include a singleton-filter which we assume would remove most of false positively called non-variants sites. As we observed that several methods are not tolerating errors well in sequencing data, we also generated additional data sets with 0.0125%, 0.025%, and 0.05% error rates for sequencing data. Errors are added incrementally from lower to higher error rated data to aid interpretability.

Furthermore, we also simulated phasing errors using a standard haplotype phasing method to investigate the effect of phasing errors. For the phasing error simulation, we simulated haplotypes of European ancestry using stdpopsim. The HapMap genetic map (GRCh37) was used to simulate haplotypes of chromosome 20. We used the population model OutOfAfrica_2T12 defined in stdpopsim and generated these 2,000 Europeans. Every two consecutive haplotypes were merged into one genotype. Then 0.1% genotyping errors were added to the panel. At the end, SHAPEIT4 [27] was used to re-phase the genotype data without using any reference. At end we had average 0.17% switching error that calculated by VCFtools [28].

UK Biobank dataset

While real data sets are often not suited for evaluating power and accuracy, they can be used to estimate the efficiency of the tools regarding the run time and memory usage. For the run time and memory usage benchmark, we used chromosome 1 of the UK Biobank. This data set contains 487,409 individuals and 53,260 markers with a total file size of 100 GB. We created subsets of the input by reducing the number of individuals from the full size to 250, 125, 62.5, 31.3, and 15.6 thousand to evaluate the scaling up of the methods. Due to potential licence conflicts, we did not run TPBWT on UK Biobank data. A set of simulated panels were created for the run time and memory tests. This data set had similar number of individuals (500,000), similar number of sites (51,190), and tested to have similar number of detected IBD segments from other tools compared to the original UK Biobank chromosome 1.

Evaluation metrics

One of the limitations of existing benchmarks is that they use non-unified definitions of metrics. Here we aim to provide a set of well-defined and standardized metrics. For IBD segment detection performance evaluation, similar to other information retrieval problems [29, 30], we consider two aspects that should be examined together. First is the precision of the reported IBD segments. Second is the ability of the method to recover the true IBD segments. The reported IBD segments cannot simply be evaluated using precision and recall since the reported segments might be partially true or a ground true IBD segment can be partially reported. Moreover, a ground truth IBD segment can be reported as multiple segments. Figure 1 shows the evaluation metrics to assess the quality of IBD segments. In the following subsections, all the metrics are described in detail.

Each evaluation metric compares the sets of ground truth IBD segments and reported IBD segments. For the comparison, one can compare the variant sites, physical locations, or genetic locations. Comparing the genetic locations (in cM) assesses the quality of IBD segment call in a wide range of applications such as genealogical search and association analysis. Considering variant sites may be more suitable for applications such as IBD mapping. However, genetic location would still largely be consistent with the variant site comparison. In this work, we calculated the evaluation measures using the genetic locations of ground truth and reported IBD segments. To observe the performance of different IBD segment lengths, ground truth IBD segments and reported IBD segments were collected as full sets, and binned sets as [2,3), [3,4), [4,5), [5,6), and $[7,\infty)$ cM bins. Then the accuracy calculations were carried on by taking each bin set as an evaluation target with the full set of ground truth as the reference set, this method overcomes a major case that a reported IBD segment's length is very close to ground truth IBD segment's length but was binned into different bin, e.g. a

2.9 cM reported IBD segment is binned into the [2,3) cM bin. But the ground truth is 3.0 cM and was divided into the [3,4) cM bin, if we only consider [2,3) cM ground truth set as the reference this almost perfect reported IBD segment will not be counted. The same approach was applied to power calculations, and we considered a full set of reported IBD results as the reference set to compute with each ground truth bin.

The reason we use these multiple of metrics is that there is no single metric that can describe all aspects of IBD segment detection performance. Indeed, depending on the downstream analysis tasks, different aspects of IBD segment detection are weighted differently. For example, genealogy inference algorithms use the total IBD segment length. Some use the total number of IBD segments, and some use the lengths of individual IBD segments. For IBD mapping, the actual set of markers that are called is more important.

Evaluation of reported segments

In order to evaluate the quality of reported segments, we define three measures: *accuracy*, *length accuracy*, and *length discrepancy*. The *accuracy* is calculated as the number of covered IBD segments divided by the total number of reported IBD segments. A reported IBD segment is considered covered, if at least 50% of its length is covered by a ground truth IBD segment. *Length accuracy* is a more fine-grained measure for reported IBD segments, and the measure accounts for the portions of falsely reported segments along a true IBD segment. To calculate the *length accuracy*, we first find the best-matching ground truth IBD segment for each reported IBD segment, i.e. the one that covers the reported IBD segment with the longest overlap. Next, the percentage of the reported IBD segment covered is calculated. Finally an average of these percentages across all reported IBD segments denotes the *length accuracy*. These accuracy measures can reflect the concept *False Positive Rate* simply by *1 - Accuracy*. *Length discrepancy* captures the length difference between the reported IBD segment and its best-matching true IBD segment. Over all segments, the root-mean-square deviation is calculated as the *length discrepancy*. For this measure, the smaller the *length discrepancy* is, the better the quality of the reported IBD segment. This measure focuses on the length of matching between the reported and the ground true IBD segments.

Detection power

We define three measures to evaluate the ability of the tools in detecting ground true IBD segments. The first measure, *recall*, denotes the proportion of the number of true IBD segments that have been reported. Here, we assume a ground truth IBD segment has been detected if a reported segment covers at least 50% of the ground truth segment. The second measure, *power*, denotes the average proportion of true IBD segments that are covered by its best-matching reported segment. For each ground truth IBD segment, its best-matching reported IBD segment is the one that has the longest overlap. The third measure, *accumulative power*, is similar to the second measure but here we consider multiple reported IBD segments. All reported IBD segments that overlap with a ground true IBD segment are being considered.

IBD coverage distribution

The whole chromosome IBD segment coverage was calculated by counting how many times each site was covered by different tools, we focused on how close each tool matched to ground truth. This visualization method could also be used to observe how each tool handled different regions. On the other hand, it also indicates special regions upon the chromosome. For the ground truth the threshold cut-off was set to 2 cM. As a result, some tools might have slightly higher values especially if they overestimate the IBD boundaries.

Relatedness inference

To evaluate the application of relatedness inference, we simply used the calculated total length of reported IBD segments between each individual pair. The threshold values [31] were calculated by the total IBD sharing to assign the degrees of relatedness up to 4th degree. We did not adjust the threshold values to account for the reduction of power that resulted from genotyping errors. The genotyping error rate was set to 0.1%. Datasets both with and without phasing error were used for this experiment.

Population and influence of marker selection

We first simulated three separated population panels and a mixed panel that contains all the three populations to observe the performance of each tool upon different population panels. In addition, considering each population has their own set of preferred markers, using a marker set designed for a different population may yield poor results. Therefore we conducted another set of experiment to observe the influence of marker selection. First, three sub panels were extracted from the mixed panel by population. Then, the ideal set of markers for each sub panel was determined using the down sampling method in simulated datasets section. After that, array data sets with all combinations between three populations and three marker sets were created with 0.1% genotyping error rate. At the end we input each tool with all these array data sets to evaluate the performance.

Run time and memory usage

The idea of run time and memory benchmarking was to give practically sufficient resources, and use increasing sizes of sample to input each tool, then observe how each tool's performance. Therefor, for each experiment, we allocated a maximum of 500 GB memory and 60 hyper threading CPU cores with 3.00 GHz clock speed in our computation node. We collected three reported pieces of information from the "sacct" command of Slurm Workload Manager version 20.02.2. The wall clock time corresponds to the "Elapsed", the CPU Time is reflected by "TotalCPU", and peak memory consumption is measured by "MaxRSS".

The wall clock time tells the user how long it takes to get the job done in real world. It gives the most direct impression of a software, regardless of time sensitive cases, however, most people still like to have the job done quickly. The CPU Time, a traditional measure of time complexity in algorithms, measures how much total workload the task really is. With advanced multi-core computer hardware, this number could be much different to the wall clock time. With the same sufficient resources a small job and a massive job could be done in the same amount of real world time, because the big job may take dozens of CPU cores but the small job may just use a fraction of a single core. The Memory consumption is used to reflect the space complexity, since hard drive storage is no longer a major limit, and memory size is the major scale-limiting factor. If a task requires too much memory, it could be practically impossible. On the other hand if an algorithm is efficient enough to solve a big problem in a small size of memory, it means tasks could be done in a practical and low-budget approach. Most importantly this means with the same amount of memory this method can solve the problem on a larger scale. The degree of parallelism describes how well a tool could fully utilize modern computer resources. The degree of parallelism is defined as the ratio of CPU Time and Wall Clock Time. If the number is very close to 1, it means the tool does not take advantage of multi-cores. On the other hand the tool is well parallel programmed if the number is much higher than 1 and even closer to the number of cores allocated. This number could also be divided by the number of cores allocated to represent CPU utilization. This measure indicates the decency of modern software engineering, and it potentially tells

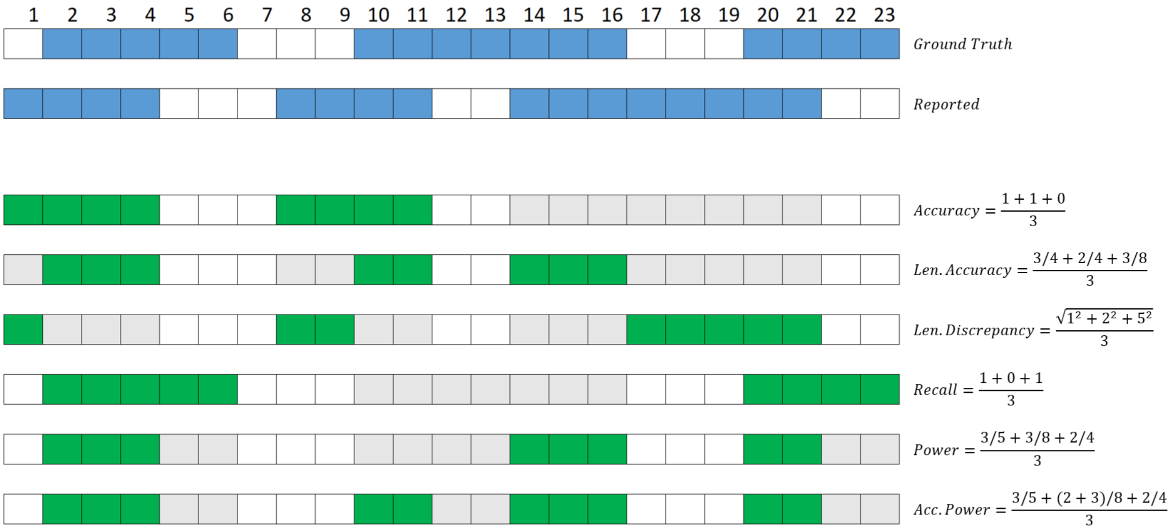

Figure 1. Demonstration of accuracy and power measures. The blue segments represent existing ground truth and reported IBD segments, the green segments donate the segments that are to be considered in calculations and the gray segments are not to be considered during calculations. Accuracy is counted if a reported IBD segment could be covered by any one ground truth IBD segment with 50%, as shown the last reported IBD segment was not considered. Length accuracy is calculated with a best ground truth IBD segment that could cover the reported IBD with maximum length. Length Discrepancy is calculated by considering the length difference with a best matching ground truth IBD segment. Recall is courted if a ground truth IBD segment could be covered with 50%, as demonstrated, the second ground truth IBD segment is not counted since none of those reported IBD segments could cover 50% of this ground truth IBD segment. Power is a similar measure to length accuracy but measures the segments of ground truth IBD segment that are covered by a best reported IBD segment. Accumulative power considers multiple reported IBD segments to cover a ground truth IBD segment.

how fast the tool could solve the problem in the real world.

Selected tools

We chose the latest generation of IBD segment detection tools that have been designed for biobank data scale in the past few years, FastSMC, hap-IBD, iLash, RaPID, and TPBWT. Since each tool has a wide range of parameter combinations, it is practically difficult to acquire the optimal parameter combination for each tool in each case. We spent the same amount of effort on each tool and tried to find proper parameters for different cases. We used default parameters if we could not find better parameters. All the parameters for different tools have been included in Supplementary Table S1.

FastSMC applies hashing methods to identify IBD segments, then uses a coalescent-based HMM (hidden Markovian model) to verify the segments. FastSMC uses an ascertained sequentially Markovian coalescent (ASMC) to estimate the posterior of the time to most recent common ancestor (TMRCA) for pairs of individuals. It has been implemented in C++ and with optional Python bindings. The input Variant Call Format (VCF) file needs to be converted to Oxford phased haplotype file, and the genetic map needs to be processed to have exact sites in the original VCF file. The software requires so-called Decoding quantities files. The files for some populations have been provided in the package.

hap-IBD uses a seed-and-extend technique with positional Burrows–Wheeler transform (PBWT)[32]. Due to the efficient extraction of haplotype matches using the (PBWT) , it is very time and memory efficient. hap-IBD has been implemented in Java, which provides conveniently cross platform execution. It does not require any data conversion of the VCF file. The required genetic map file format is PLINK, while the sites in the map file do not have to be the exact sites in the VCF file. hap-IBD is well engineered to execute in parallel.

iLash applies sliding, minhashing, locality sensitive hashing and pairwise extension to report IBD segments. iLash was programmed in C++, it also supports parallel execution. The input VCF and genetic map files need to be converted to PLINK PED and MAP formats.

RaPID leverages multiple random projections to perform approximate haplotype matching, then applies PBWT and merging methods for IBD detection. RaPID has been implemented in C++. The input file should be in compressed VCF format, and the genetic map needs to be preprocessed to match exact sites in the VCF file. The command line parameters can be calculated with provided python script considering genotyping error rate, marker density, and the minimum length of IBD segments. The author also has recommended a set of parameters for general cases.

TPBWT extends PBWT by adding a new dimension to the PBWT that allows mismatches in detected IBD segments. The added dimension simply masks out potential errors in the haplotypes and extends IBD segments even if there is a mismatch between the haplotypes. The argument template, a two dimensional python list, defines the configurations that can tolerate genotyping and/or phasing errors. Similar to RaPID, TPBWT also requires a genetic map to be formatted to match exact sites in the VCF input file. TPBWT has been implemented in Python.

Results

Overall evaluation of IBD segment detection tools

Figure 2 shows the values for five different metrics in both sequencing and array data: accuracy, length accuracy, length discrepancy, power, and accumulative power. These metrics can be applied to assess the quality of IBD calls for a variety of applications such as IBD mapping, genealogical inference. The other two metrics, recall and length discrepancy metrics are specially useful for applications that summarize the IBD counts and length. e.g. investigation of population history, pedigree inference.

The genotyping error was set to 0.1% which is the expected [33, 34, 35] error rate in available data. As shown in Figure 2, FastSMC and iLash both had high accuracy in 2 cM segments and overall lower power. hap-IBD's accuracy is slightly lower for 2 cM segments, but it has significantly higher detection power. RaPID had high power and competable accuracy on longer segments. Both RaPID and TPBWT have relatively low length discrepancy in both sequencing and array data, and higher detection power in sequencing data. The accuracy of TPBWT in sequencing data is lower, especially for shorter segments. For long segments ($\geq$ 10 cM), all selected

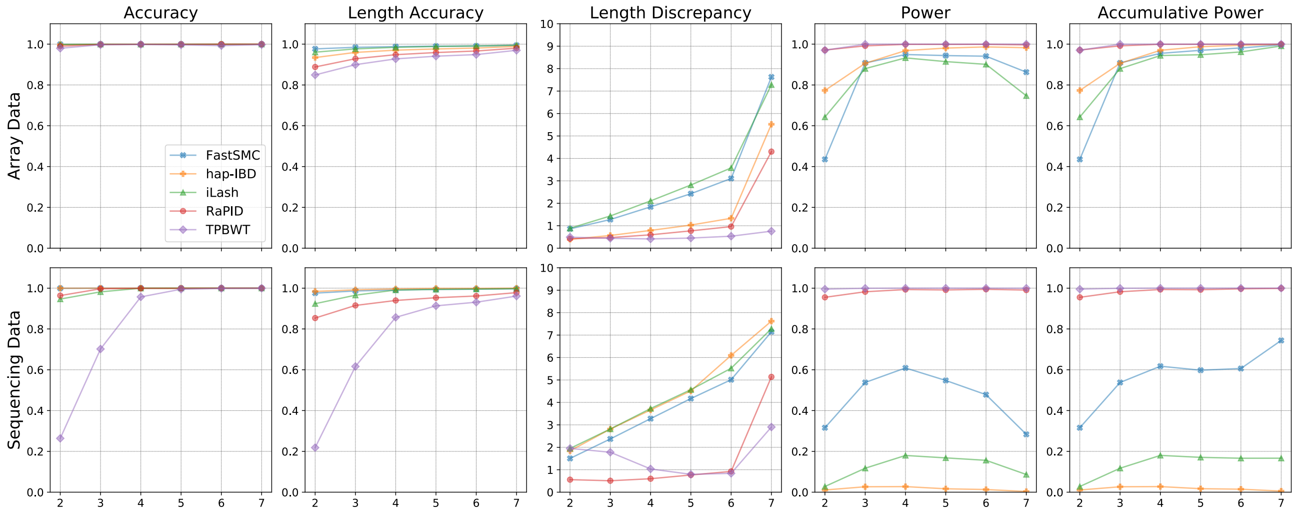

Figure 2. Benchmarking results of different IBD segment detection tools in EUR array and sequencing data with a genotyping error rate of 0.1% stratified by segment lengths. Some tools have reduced detection power as IBD segment cut-off increases. It is likely caused by fractured IBD segments since this measure only considers one reported IBD segment. Accumulative power considers all reported overlap IBD segments and all corresponding values are monotonically increasing. The length discrepancy is measured by cM, other measures are based on percentage.

tools have high power and accuracy on inputs without genotyping error. When dealing with genotyping errors, reported segments tend to break into pieces resulting in low power. Overall, TPBWT has the highest detection power on sequencing data with genotyping error followed by RaPID (see Supplementary Figures S1 and S2).

Regional coverage of IBD segments

Figure 3 shows IBD segments coverage results with 0.1% genotyping error rate. The results of IBD segments coverage without genotyping error are also included in Supplementary Figure S3. Overall, while the ground truth IBD coverage is roughly even across the chromosome, all methods produced IBD calls with variability in coverage. For array data, TPBWT and RaPID have over-calling while iLash and FastSMC have under-calling. hap-IBD has the best calibration in terms of overall coverage. For sequencing data, all methods have much greater variability in IBD coverage. IBD coverage of RaPID was the closest to the ground truth in sequencing data.

Based on our results, we inferred that most of the tools were well-configured to handle array data. hap-IBD and iLASH have very low detection power for sequencing data with errors. We conjecture that the default parameters for these tools do not offer competitive results for sequencing data. We noticed that TPBWT has a much higher false positive rates in EAS and EUR than in AFR, this further tells that either new sets of sequencing-specific parameters are needed or a pre-processing to thin the panel is required.

Effect of genotyping errors

Although most current human genetic panels have very low genotyping error rate, some historic data and some non-human data may still have significantly higher genotyping error rates. Increasing genotyping errors will likely decrease the detection power. Figure 4 shows the results of different tools in both array and sequencing data using a relatively high genotyping error rate (0.4%). The EUR results for 0%, 0.2% and 0.3% genotyping error rates. The results for other two populations and the mixed population are shown in the Supplementary Figures S4-S21. Overall, TPBWT and RaPID seem to be more robust against high genotyping errors while TBPWT's detection power remains higher with slightly lower accuracy compared to RaPID in sequencing data. The detection power of hap-IBD, FastSMC and iLASH are impacted noticeably by higher genotyping errors. The results with lower genotyping errors in sequencing data have been included in the Supplementary Figures S22-S25. The recall results are included in the Supplementary Figures S26-S28. The recall values are overall consistent with the power values. Length discrepancies of RaPID and TPBWT are lower, especially for longer segments in the presence of genotyping errors. This reflects that other methods tend to break long IBD segments into pieces and thus length discrepancy increased significantly.

Relatedness inference

As shown in Figure 5, the simulated data has a realistic distribution of close relatives, where the number of relative pairs increases exponentially with the degree of relatedness. For relatedness inference, we found that, on array data, all methods achieved a decent calls. This is understandable as calling close relatives mainly relies on the accumulated power for long segments, and all methods are very capable of doing that. hap-IBD, TPBWT, and RaPID have closest reported pairs to ground truth. hap-IBD had slightly less reported pairs, while TPBWT and RaPID tended to report a few more pairs. Both FastSMC and iLash tended to report fewer number of pairs in most of the cases while FastSMC tended report more pairs in first degree. On sequencing data, not all methods are well-calibrated. The power of hap-IBD was most-severely reduced, followed by iLash. FastSMC had less impact by genotyping error, had closer number of reported pairs. Both TPBWT and RaPID had decent power, though TPBWT has a tendency of over-calling in 4-th and 3-rd degree relatives in EAS and EUR. All tools had similar good performance on data without genotyping error (See Supplementary Figure S29). Although phasing errors may break long IBD segments into smaller pieces, Supplementary Figure S30 shows that phasing errors had little impact on the relatedness detection.

Performance in different populations and marker sets

As shown in Figure 3 and Figure 5, although different populations has different marker density and average IBD coverage, the performance of all methods were relatively stable. A main outlier was that TPBWT has a much higher false positive rates in EAS and EUR than in AFR. This may be due to lower level of linkage disequilibrium in

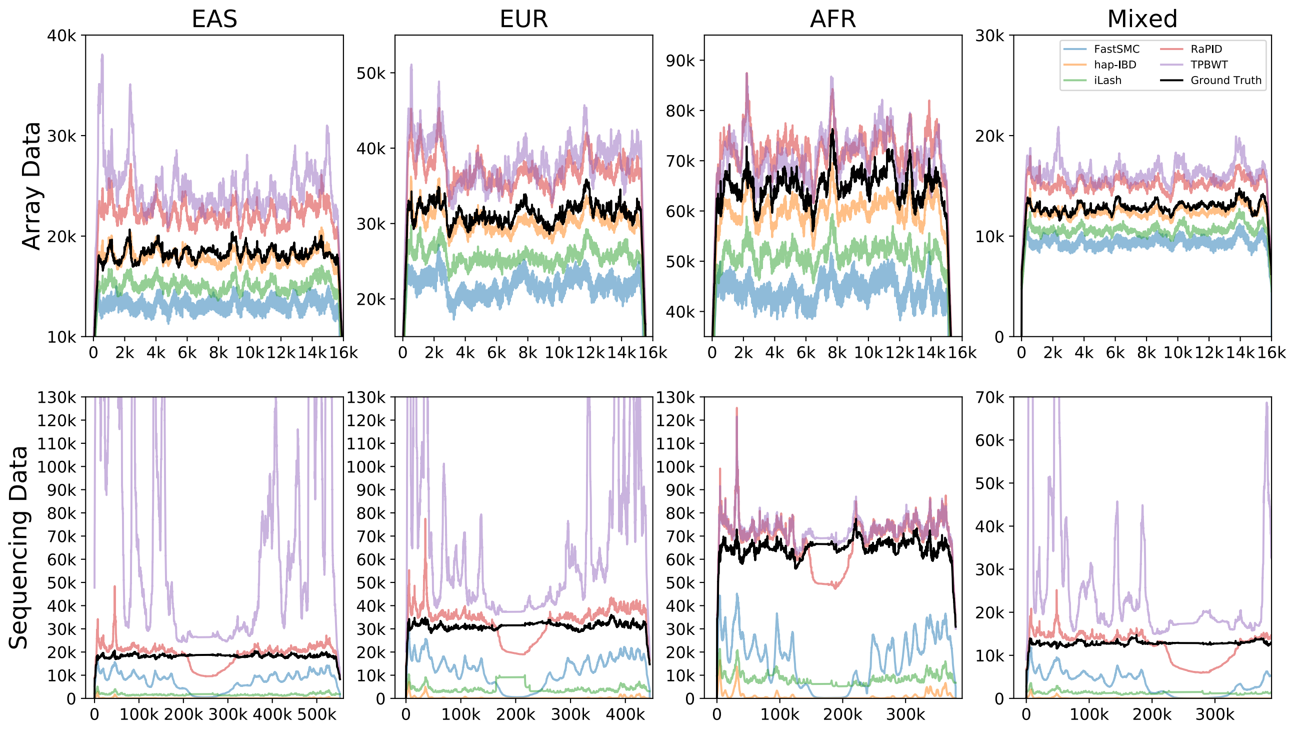

Figure 3. Visualization of IBD segment coverage (over 2 cM) in array and sequencing data with a genotyping error rate of 0.1% on three populations. The x-axises are the marker location indexes. The y-axises are number of IBD segments that covered each marker. The ground truths were displayed (black) for reference purpose.

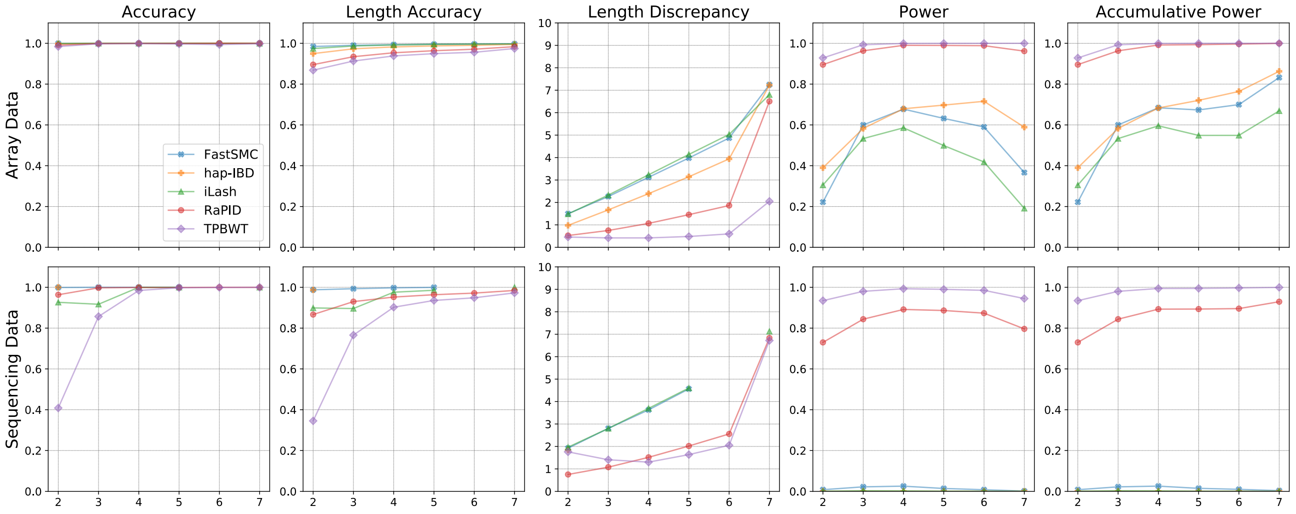

Figure 4. Benchmarking results of different IBD segment detection tools in EUR array and sequencing data with a genotyping error rate of 0.4% stratified by segment lengths. The length discrepancy is measured by cM, other measures are based on percentage. Some lines may be discontinued or dipped due to low power.

AFR. Based on our observations there was no significant difference between the mixed population panel and the separated panels.

The results on the marker set selection (Supplementary Figures S31 and S32) show that using a set of markers designed for a different population has noticeably lower accuracies, especially in terms of length accuracy and length discrepancy at shorter target lengths. Powers, on the other hand, were slightly increased. This is because that the set of markers designed for a different population has lower MAF than the set of markers designed for the population of interest and thus increases the chance of random allele matches. However, the overall effects of suboptimal marker set selection are minor in our experiments.

Robustness against phasing errors

The benchmarking results for all tools in the presence of phasing error are included in Supplementary Tables S2-S7. The reduction of power is from 4-7% for different tools for short segments (2-3 cM). The reduction in power for very long segments ($\geq$ 15 cM) ranges from 5-20% without any special treatment of phasing errors, with a strict threshold cut-off length. However, including shorter segments results in 4-10% differences in detection power for very long segments. TPBWT with phasing error tolerance had only 5% reduction in detection power for very long segments ($\geq$ 15 cM) with minor reduction in length accuracy. TPBWT with phasing error tolerance mode was also able to increase its detection power for shorter segments by almost 2% for 2 cM segments, while the reduction in accuracy/length accuracy was more noticeable. In general,

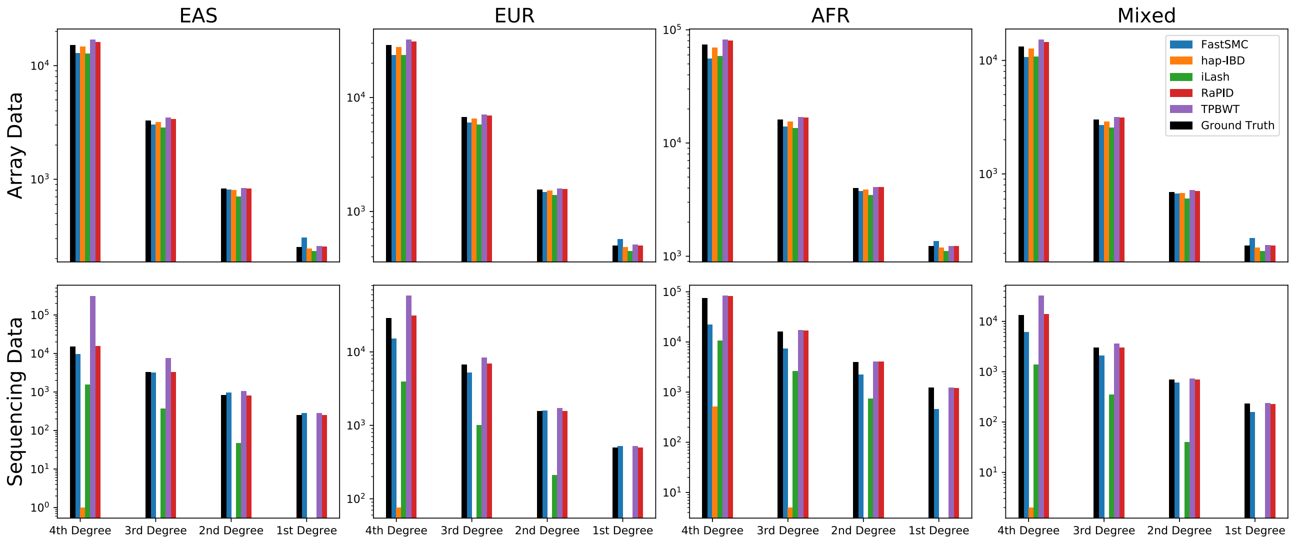

Figure 5. Relatedness detection results in array and sequencing data with a genotyping error rate of 0.1% on four population panels. The y-axises are number of individual pairs were found in each category. The ground truths were displayed (black bars) for reference purpose.

the reduction of detection power after the data were re-phased is not very significant for short IBD segments. This is due to the high accuracy of the current phasing algorithms with the availability of large biobank scale cohorts. The phased data may contain some long-range switch errors or blips, but they will not contribute to a noticeable reduction in the detection power except for strict and very long IBD cut-off thresholds. The possible reduction of power can also be alleviated for some downstream analysis, especially if the total shared IBDs between two individuals is of interest [36]. However, the impact of phasing errors could be more consequential if the number of segments is being considered.

Run time and memory useage

The run time and memory consumption experiments were carried out on our 500 GB memory server as mentioned, iLash and TPBWT could not finish all the experiments with 500 GB memory. Due to potential licence conflicts we did not run TPBWT on UK Biobank data, the time and memory consumption results for TPBWT were based on the simulated dataset as mentioned in the method section. Since FastSMC requires the most recent updated operating system and libraries, this could be a problem since most of the servers do not use the latest versions. We were able to make FastSMC executable on a 32 GB memory PC for accuracy and power assessment through some efforts. Thus, we were not able to run FastSMC for large panels. We ran FastSMC on panels with smaller sample sizes (from 1,000 to 31,000) on the 32 GB PC. By measuring run times and memory consumptions of these smaller inputs, with an extrapolation using second order polynomial regressions, we estimated the FastSMC run time would be 126 days and memory consumption would be 6.5 TB for the whole chromosome 1 of UK Biobank data. Therefore we did not include FastSMC in Figure 6. More details can be found in the Supplementary Table S8.

hap-IBD had the shortest wall clock time due to the efficient underlying method and efficient parallelization. It took around 0.5 hours for hap-IBD to complete the IBD segment calls for UK Biobank chromosome 1 with 2 cM IBD cut-off. iLash won the second place on wall-clock time, but as mentioned it could not finish some large cases. Both hap-IBD and iLash take a good advantage of parallel execution. hap-IBD was able to utilize about 75% of the CPU resource as the degree of parallelization of 45.27 out of 60. iLash reached an average parallelization of 9.23 out of 60. CPU time of RaPID was the shortest among all the tools for longer segments (e.g. 5 cM). hap-IBD had the lowest CPU time for short IBD segments (2 cM). The CPU time for TPBWT was also noticeably short for smaller panels (e.g. 15 K) but the time increased fast with the increasing sample size. iLash and TPBWT did not complete the task with the largest panels within the provided memory.

As shown in Tables 1 and 2, RaPID requires the least amount of memory. The memory consumption of RaPID was limited to less than 8 GB, while some other tools required hundreds of gigabytes of memory. hap-IBD also has an efficient memory usage with the maximum memory usage of about 112 GB memory. The memory consumption results for 3 cM and 5 cM length cut-offs can be found in Supplementary Tables S12 and S13. We observed the memory consumption of hap-IBD and iLash for the simulated data set doubled compared to the original UK Biobank data, so we predict that TPBWT could finish the original 250 thousand individual subset of UK Biobank chromosome 1. The run time and memory consumption results for this simulated set can be found in Supplementary Tables S8-S10.

Conclusion and Discussion

We conducted the first open-source fully-transparent comprehensive benchmarking of modern efficient IBD segment detection methods. Based on our benchmarking, most tools yield decent results in most settings, even in the presence of phasing errors. However, genotyping error rates and marker densities would affect the performance of IBD detection methods in different degrees.

For short IBD segments (e.g. 2 cM), FastSMC and iLash have high accuracies but low power in both sequencing and array density. Both hap-IBD and RaPID have high power and relatively lower accuracy in array data, and TPBWT achieved highest detection power in sequencing data followed by RaPID, especially in the presence of high genotyping errors. For longer IBD segments ($\geq 5cM$), all five tools have comparable accuracy, while hap-IBD, RaPID and TPBWT have high power in array data. iLASH and FastSMC may report IBD segments as multiple short segments especially for longer segments (e.g. 5 cM). The accuracy of most tools for long segments ($\geq$ 10 cM) is high but the detection power of some tools is affected significantly with genotyping errors. TPBWT and RaPID have the highest detection power for sequencing data in the presence of genotyping errors, with TPBWT being the most robust against high genotyping error rates.

In general, the minimum length threshold impacts the accuracy

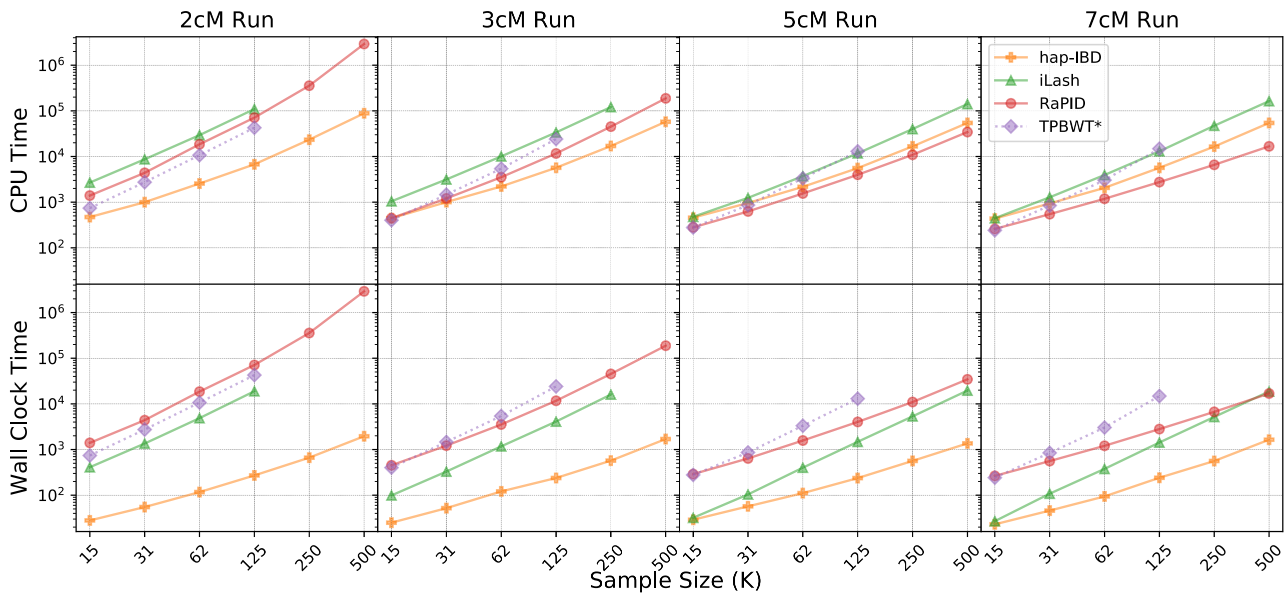

Figure 6. Run time results based on UK Biobank Chromosome 1. CPU Time and Wall Clock Time are displayed in the first and second row. Each column of the subfigures represents runs with different IBD segment cut-off (2, 3, 5, and 7 cM). The x-axises in each subfigure are the input size in thousand individuals. The TPBWT's results were based on the simulated panels.

Table 1. Memory consumptions based on UK Biobank chromosome 1 with 2 cM length cut-offs.

	Sample Size (K)					
	15	31	62	125	250	500
hap-IBD	1.76 MB	7.88 GB	8.79 GB	22.81 GB	59.03 GB	110.15 GB
iLash	7.85 GB	20.05 GB	58.73 GB	191.46 GB	na	na
RaPID	728.72 MB	1.03 GB	1.54 GB	2.47 GB	4.14 GB	7.25 GB
TPBWT*	7.70 GB	30.59 GB	122.16 GB	488.40 GB	na	na

Table 2. Memory consumptions based on UK Biobank chromosome 1 with 7 cM length cut-offs.

	Sample Size (K)					
	15	31	62	125	250	500
hap-IBD	1.20 MB	9.93 GB	23.24 GB	15.62 GB	31.78 GB	110.03 GB
iLash	1.77 MB	5.39 GB	15.25 GB	30.59	77.48 GB	178.83 GB
RaPID	347.13 MB	718.97 MB	1.22 GB	2.21 GB	3.90 GB	6.33 GB
TPBWT*	7.70 GB	30.60 GB	122.16 GB	488.40 GB	na	na

as reflected in our results. We acknowledge that the methods are more likely to report an IBS as an IBD segment for shorter segments. The minimum threshold length can vary for different applications for example for genealogical search long IBD segments (e.g. 5 or 7 cM) may be used, while for IBD mapping, shorter IBD segment (e.g. 2 or 3 cM) might be used. Our aim was to benchmark the performance of different tools for different IBD segment lengths that provide the researchers with the expected accuracy for their applications.

FastSMC, hap-IBD and iLASH have high accuracy in sequencing data especially for short segments (e.g. 2 cM), but their detection power is low. The accuracy of all tools are relatively high for long IBD segments (e.g. 5 cM). Genotyping errors in the sequencing data may affect the results of hap-IBD, FastSMC and iLASH significantly. A possible solution to run these tools could be to down sampling the data using MAF.

Most IBD segment detection tools can handle VCF files. hap-IBD does not require any data preparation while RaPID and TPBWT require minor data preparation. FastSMC and iLash require some data conversion if the available data are in VCF format. FastSMC also requires additional files which are population specific.

Regarding the resource requirements, only RaPID and hap-IBD were able to be used with limited resources, while others require significantly more memory for large panels. RaPID was the most memory efficient tool which took 12 times less memory than hap-IBD. On the other hand, hap-IBD had the highest resource utilization followed by iLash. hap-IBD was also the fastest method for short IBD cut-off lengths in our experiments, while RaPID had less CPU Time for longer IBD cut-off (e.g. 5 cM).

Our study has several limitations. First, as mentioned, the parameter combination for each tool may not be optimal for each case. Finding out the best combinations for each tool is beyond the scope of this work. However, we open-sourced our entire benchmarking protocol and thus we hope others can contribute and release their optimized parameters on the same data sets. Second, although simulated data sets are somehow reflected in the real world, there is still a possibility that the real data could have some unique characteristics. In general, it is not straightforward to extract ground true IBD segments from real data. The availability of several pedigree data may provide the opportunity to extract ground truth from real data. The same evaluation metrics, however, then can be used to evaluate the performance of IBD segment detection tools. Moreover, we only

simulated the data with one simulation model. Benchmarking of diverse populations with different models is warranted for future research. The singletons in the simulated data were filtered before introducing genotyping error, thus, we did not consider the fact that genotyping errors are more likely to happen to singletons.

Availability of Source Code and Requirements

- Project name: IBD Detection Tool Benchmark Project
- Project home page: https://github.com/ZhiGroup/IBD_benchmark
- Operating systems: Linux and Windows
- Programming language: C#
- Licence: MIT
- RRID: SCR_022850
- biotools: ibd_benchmark_tool

Availability of Supporting Data and Materials

The source code and a set of demonstration data is available at: GitHub[37]. The data sets supporting the results of this article are available in the GigaScience Database [38]. The UK Biobank Resource is retrieved under Application Number 24247.

The FastSMC [19] software package and source code are available at: FastSMC's GitHub[39]. The hap-IBD [20] software package and source code are available at: hap-IBD's GitHub[40]. The iLash [21] software package and source code are available at: iLash's GitHub[41]. The RaPID [18] software package and source code are available at: RaPID's GitHub[42]. The TPBWT [22] software package and source code are available at: TPBWT's GitHub[43].

Additional Files

Supplementary Figures S1 and S2 Benchmarking results of different tools in EUR data on longer IBD segments.
Supplementary Figure S3. Visualization of IBD segment coverage in array and sequencing data without genotyping on three populations.
Supplementary Figures S4-S21. Benchmarking results of EUR results for 0%, 0.2% and 0.3% genotyping error rates, and results for EAS, AFR, and mixed populations.
Supplementary Figures S22-S25. Benchmarking results of sequencing data with low genotyping error rates.
Supplementary Figures S26-S28. Benchmarking results of IBD segments recall.
Supplementary Figure S29. Relationship detection results in array and sequencing data without genotyping errors on EAS, EUR, AFR, and mixed populations.
Supplementary Figure S30. Relationship detection results in array with both genotyping errors and phasing errors on EAS, EUR, AFR, and mixed populations.
Supplementary Figures S31 and S32. Benchmarking results of different IBD detection tools on AFR and EAS array data using a set of markers designed for a different population.
Supplementary Table S1. Parameters and command lines for benchmarking different IBD detection tools.
Supplementary Tables S2-S7. Effect of phasing error on the performance of IBD detection tools.
Supplementary Tables S8-S10. Run time and memory result on simulated big panels.
Supplementary Table S11. FastSMC run time and memory usage results on a 6 core 3.5 GHz CPU and 32 GB memory PC.
Supplementary Tables S12 and S13. Memory usage based on UK Biobank Chromosome 1 with IBD 3 cM and 5 cM length cut-offs by increasing sample size.

Declarations

List of Abbreviations

ASMC: ascertained sequentially Markovian coalescent; cM: centi-Morgan; DTC: direct to consumer; HMM: hidden Markov model; IBD: Identical-By-Descent; MAF: minor allele frequency; MRCA: most recent common ancestor; PBWT: positional Burrows–Wheeler transform; TMRCA: time to most recent common ancestor; VCF: Variant Call Format.

Consent for publication

Not applicable.

Competing interests

The authors declare that they have no competing interests.

Funding

This work was supported by the National Institutes of Health grants R01 HG010086 and R56 HG011509.

Authors' contributions

SZ and DZ conceived and designed the study. KT, AN, YW, SZ, and DZ developed the method. All authors conducted the analyses and interpretation of the results. All authors contributed to the writing of the manuscript. All authors read and approved the final manuscript.

Acknowledgement

The authors acknowledge Olivia Tyndall at the University Writing Center of University of Central Florida for her professional English editing advice. This research has been conducted using the UK Biobank Resource under Application Number 24247.

References

1. Browning BL, Browning SR. A fast, powerful method for detecting identity by descent. The American Journal of Human Genetics 2011;88(2):173–182.
2. Thompson EA. Identity by descent: variation in meiosis, across genomes, and in populations. Genetics 2013;194(2):301–326.
3. Ramstetter MD, Dyer TD, Lehman DM, Curran JE, Duggirala R, Blangero J, et al. Benchmarking relatedness inference methods with genome-wide data from thousands of relatives. Genetics 2017;207(1):75–82.
4. Hill WG, Weir BS. Variation in actual relationship as a consequence of Mendelian sampling and linkage. Genetics research 2011;93(1):47–64.
5. Chen H, Naseri A, Zhi D. FiMAP: A Fast Identity-by-Descent Mapping Test for Biobank-scale Cohorts. medRxiv 2021;.
6. Browning SR, Thompson EA. Detecting rare variant associations by identity-by-descent mapping in case-control studies. Genetics 2012;190(4):1521–1531.
7. Houwen RH, Baharloo S, Blankenship K, Raeymaekers P, Juyn J, Sandkuijl LA, et al. Genome screening by searching for shared segments: mapping a gene for benign recurrent intrahepatic cholestasis. Nature genetics 1994;8(4):380–386.
8. Gusev A, Kenny EE, Lowe JK, Salit J, Saxena R, Kathiresan S, et al. DASH: a method for identical-by-descent haplotype map-

ping uncovers association with recent variation. The American Journal of Human Genetics 2011;88(6):706–717.
9. Vacic V, Ozelius LJ, Clark LN, Bar-Shira A, Gana-Weisz M, Gurevich T, et al. Genome-wide mapping of IBD segments in an Ashkenazi PD cohort identifies associated haplotypes. Human molecular genetics 2014;23(17):4693–4702.
10. Abney M, ElSherbiny A. Kinpute: using identity by descent to improve genotype imputation. Bioinformatics 2019;35(21):4321–4326.
11. Loh PR, Palamara PF, Price AL. Fast and accurate long-range phasing in a UK Biobank cohort. Nature genetics 2016;48(7):811–816.
12. Delaneau O, Zagury JF, Robinson MR, Marchini JL, Dermitzakis ET. Accurate, scalable and integrative haplotype estimation. Nature communications 2019;10(1):1–10.
13. Henn BM, Hon L, Macpherson JM, Eriksson N, Saxonov S, Pe'er I, et al. Cryptic distant relatives are common in both isolated and cosmopolitan genetic samples. PloS one 2012;7(4):e34267.
14. Guerrini CJ, Robinson JO, Petersen D, McGuire AL. Should police have access to genetic genealogy databases? Capturing the Golden State Killer and other criminals using a controversial new forensic technique. PLoS biology 2018;16(10):e2006906.
15. Purcell S, Neale B, Todd-Brown K, Thomas L, Ferreira MA, Bender D, et al. PLINK: a tool set for whole-genome association and population-based linkage analyses. The American journal of human genetics 2007;81(3):559–575.
16. Gusev A, Lowe JK, Stoffel M, Daly MJ, Altshuler D, Breslow JL, et al. Whole population, genome-wide mapping of hidden relatedness. Genome research 2009;19(2):318–326.
17. Browning BL, Browning SR. Improving the accuracy and efficiency of identity-by-descent detection in population data. Genetics 2013;194(2):459–471.
18. Naseri A, Liu X, Tang K, Zhang S, Zhi D. RaPID: ultra-fast, powerful, and accurate detection of segments identical by descent (IBD) in biobank-scale cohorts. Genome biology 2019;20(1):1–15.
19. Nait Saada J, Kalantzis G, Shyr D, Cooper F, Robinson M, Gusev A, et al. Identity-by-descent detection across 487,409 British samples reveals fine scale population structure and ultra-rare variant associations. Nature communications 2020;11(1):1–15.
20. Zhou Y, Browning SR, Browning BL. A fast and simple method for detecting identity-by-descent segments in large-scale data. The American Journal of Human Genetics 2020;106(4):426–437.
21. Shemirani R, Belbin GM, Avery CL, Kenny EE, Gignoux CR, Ambite JL. Rapid detection of identity-by-descent tracts for mega-scale datasets. Nature communications 2021;12(1):1–13.
22. Freyman WA, McManus KF, Shringarpure SS, Jewett EM, Bryc K, 23, et al. Fast and robust identity-by-descent inference with the templated positional Burrows–Wheeler transform. Molecular Biology and Evolution 2021;38(5):2131–2151.
23. Kelleher J, Etheridge AM, McVean G. Efficient coalescent simulation and genealogical analysis for large sample sizes. PLoS computational biology 2016;12(5):e1004842.
24. Sudlow C, Gallacher J, Allen N, Beral V, Burton P, Danesh J, et al. UK biobank: an open access resource for identifying the causes of a wide range of complex diseases of middle and old age. PLoS medicine 2015;12(3):e1001779.
25. Gutenkunst RN, Hernandez RD, Williamson SH, Bustamante CD. Inferring the joint demographic history of multiple populations from multidimensional SNP frequency data. PLoS genetics 2009;5(10):e1000695.
26. Consortium IH, et al. A second generation human haplotype map of over 3.1 million SNPs. Nature 2007;449(7164):851.
27. Delaneau O, Zagury JF, Robinson MR, Marchini JL, Dermitzakis ET. Accurate, scalable and integrative haplotype estimation. Nature communications 2019;10(1):1–10.
28. Danecek P, Auton A, Abecasis G, Albers CA, Banks E, DePristo MA, et al. The variant call format and VCFtools. Bioinformatics 2011;27(15):2156–2158.
29. Buckland M, Gey F. The relationship between recall and precision. Journal of the American society for information science 1994;45(1):12–19.
30. Zuva K, Zuva T. Evaluation of information retrieval systems. AIRCC's International Journal of Computer Science and Information Technology 2012;4(3):35–43.
31. Manichaikul A, Mychaleckyj JC, Rich SS, Daly K, Sale M, Chen WM. Robust relationship inference in genome-wide association studies. Bioinformatics 2010;26(22):2867–2873.
32. Durbin R. Efficient haplotype matching and storage using the positional Burrows–Wheeler transform (PBWT). Bioinformatics 2014;30(9):1266–1272.
33. Saunders IW, Brohede J, Hannan GN. Estimating genotyping error rates from Mendelian errors in SNP array genotypes and their impact on inference. Genomics 2007;90(3):291–296.
34. Yuan M, Fang H, Zhang H. Correcting for differential genotyping error in genetic association analysis. Journal of human genetics 2013;58(10):657–666.
35. Wall JD, Tang LF, Zerbe B, Kvale MN, Kwok PY, Schaefer C, et al. Estimating genotype error rates from high-coverage next-generation sequence data. Genome research 2014;24(11):1734–1739.
36. Naseri A, Shi J, Lin X, Zhang S, Zhi D. RAFFI: Accurate and fast familial relationship inference in large scale biobank studies using RaPID. PLoS genetics 2021;17(1):e1009315.
37. Tang K, Naseri A, Wei Y, Zhang S, Zhi D, IBD Detection Tool Benchmark Project; 2022. https://github.com/ZhiGroup/IBD_benchmark.
38. Tang K, Naseri A, Wei Y, Zhang S, Zhi D. Supporting data for "Open-source benchmarking of IBD segment detection methods for biobank-scale cohorts" 2022;http://dx.doi.org/10.5524/102319.
39. Nait Saada J, Kalantzis G, Shyr D, Cooper F, Robinson M, Gusev A, et al., FastSMC; 2021. https://github.com/PalamaraLab/FastSMC.
40. Zhou Y, Browning SR, Browning BL, hap-ibd; 2020. https://github.com/browning-lab/hap-ibd.
41. Shemirani R, Belbin GM, Avery CL, Kenny EE, Gignoux CR, Ambite JL, iLASH: Ultra-Rapid Detection of IBD Tracts; 2021. https://github.com/roohy/iLASH.
42. Naseri A, Liu X, Tang K, Zhang S, Zhi D, Random Projection-based IBD Detection (RaPID); 2019. https://github.com/ZhiGroup/RaPID.
43. Freyman WA, McManus KF, Shringarpure SS, Jewett EM, Bryc K, 23, et al., 23andMe/phasedibd; 2021. https://github.com/23andMe/phasedibd.

Click here to access/download
Supplementary Material
R2_Supplementary_IBD_BM.docx

latex main figure

Click here to access/download
Supplementary Material
CEU.e0.001.svg

latex main figure

Click here to access/download
Supplementary Material
CEU.e0.004.svg

latex main figure

Click here to access/download
Supplementary Material
f4_Times_d600.svg

latex main figure

Click here to access/download
Supplementary Material
IBD_PairCnt.e0.001.svg

latex main figure

Click here to access/download
Supplementary Material
Relation.e0.001.v2.svg

 Click here to access/download;Personal Cover;cover-v3.docx

Dear editor,

Here is our response to your editorial queries:

1. Add ORCIDs. done.
2. Register RRID and biotool ID. done.
3. Change section title "Availability of Supporting Data and Materials" to "Data Availability"

   We did not make the change.

   This section not only has data information, but mostly software information.

   We checked on their published papers, there are some paper using "Availability of Supporting Data and Materials"

   And that is a title from the template.

4. Cite this paper's GigaDB link. done
5. Cite all tools' GitHub. done
6. Change section title "Abbreviations" to "List of Abbreviations". done

Thanks,
