# Supplementary material for: Open-source benchmarking of IBD segment detection methods for biobank-scale cohorts
Source: Gigascience. 2022 Dec 6;11:giac111. doi: 10.1093/gigascience/giac111 (PMC9724555; doi:10.1093/gigascience/giac111)
Supplement: giac111_Supplemental_File [file giac111_supplemental_file.docx]

# Supplementary Information

for

# Open-source Benchmarking of IBD segment detection methods for biobank-scale cohorts

Kecong Tang^1^, Ardalan Naseri^2^, Yuan Wei^1^, Shaojie Zhang^1,^* and Degui Zhi^2,^ *

^1^Department of Computer Science, University of Central Florida, Orlando, FL,32816, USA

^2^School of Biomedical Informatics, The University of Texas Health Science Center at Houston, Houston, TX 77030, USA

*Shaojie.Zhang@ucf.edu; Degui.Zhi@uth.tmc.edu


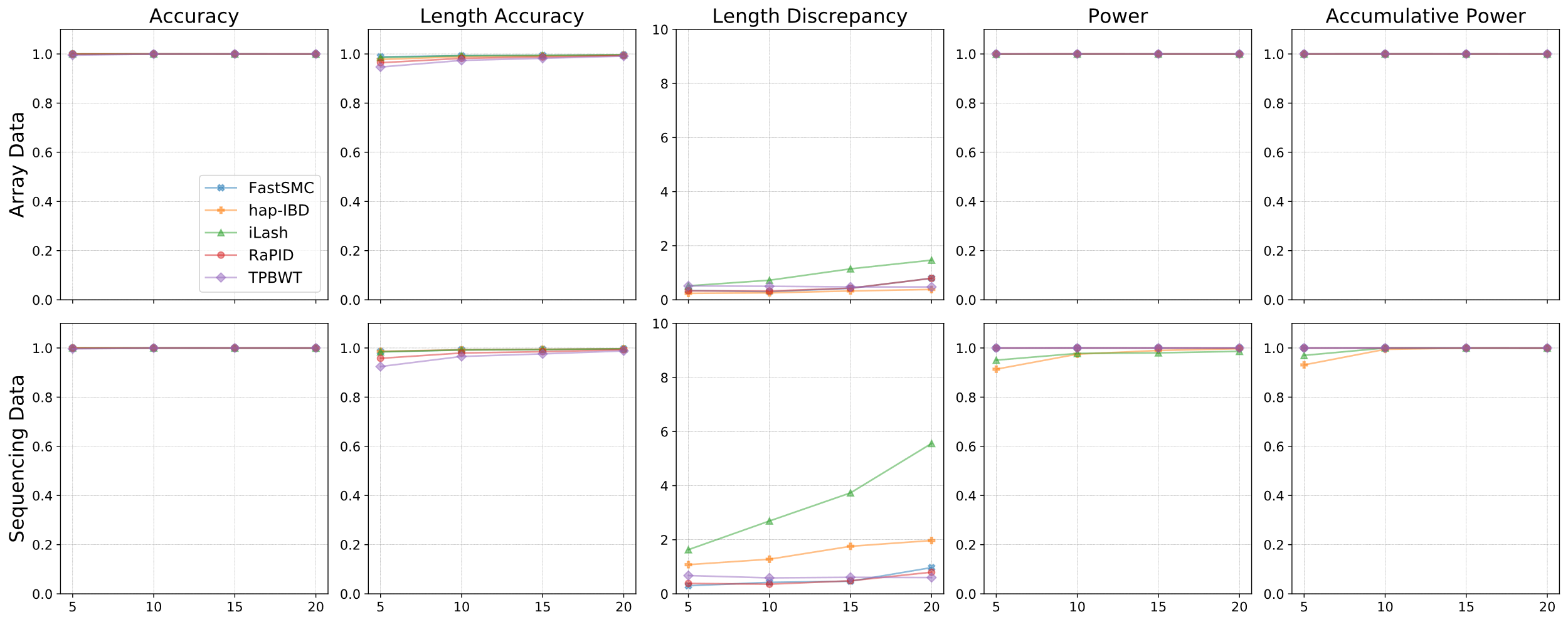
Supplementary Figure S1. Benchmarking results of different tools in EUR data on longer IBD segments without genotyping error.


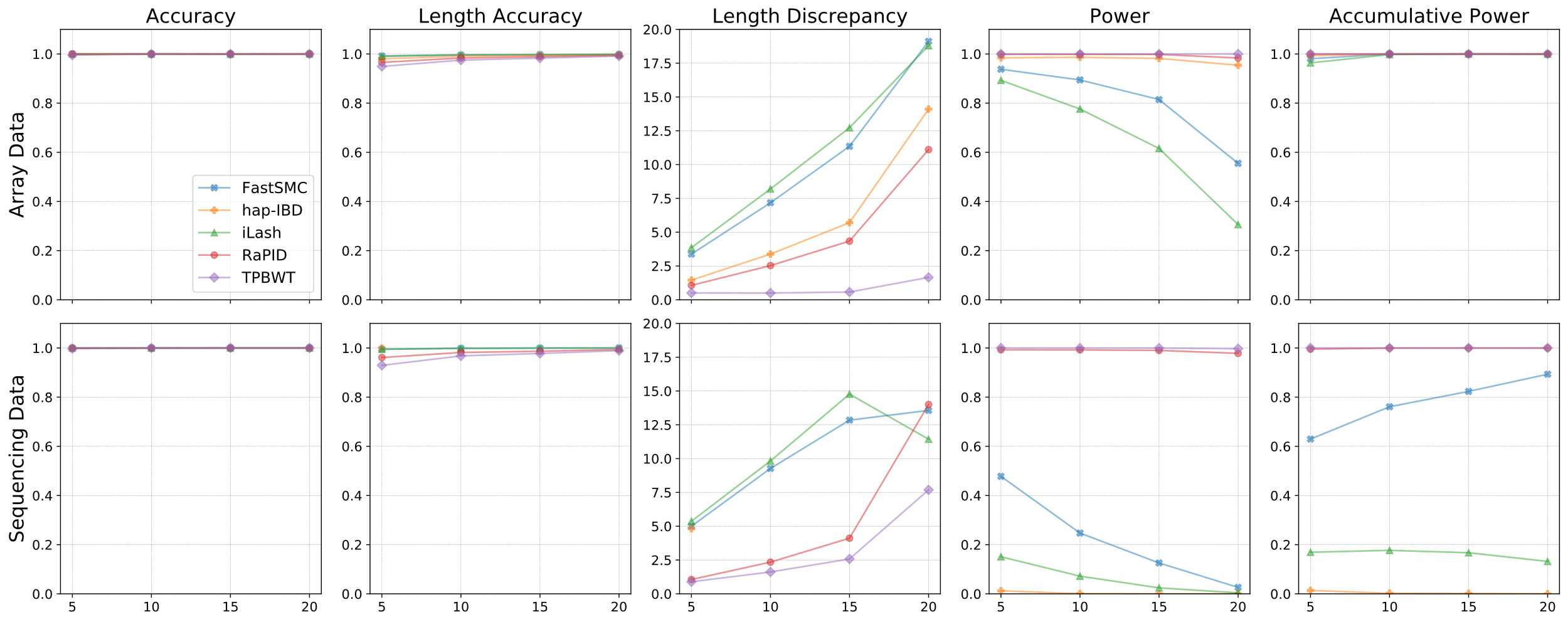


Supplementary Figure S2. Benchmarking results of different tools in EUR data on longer IBD segments with 0.1% genotyping error rate.


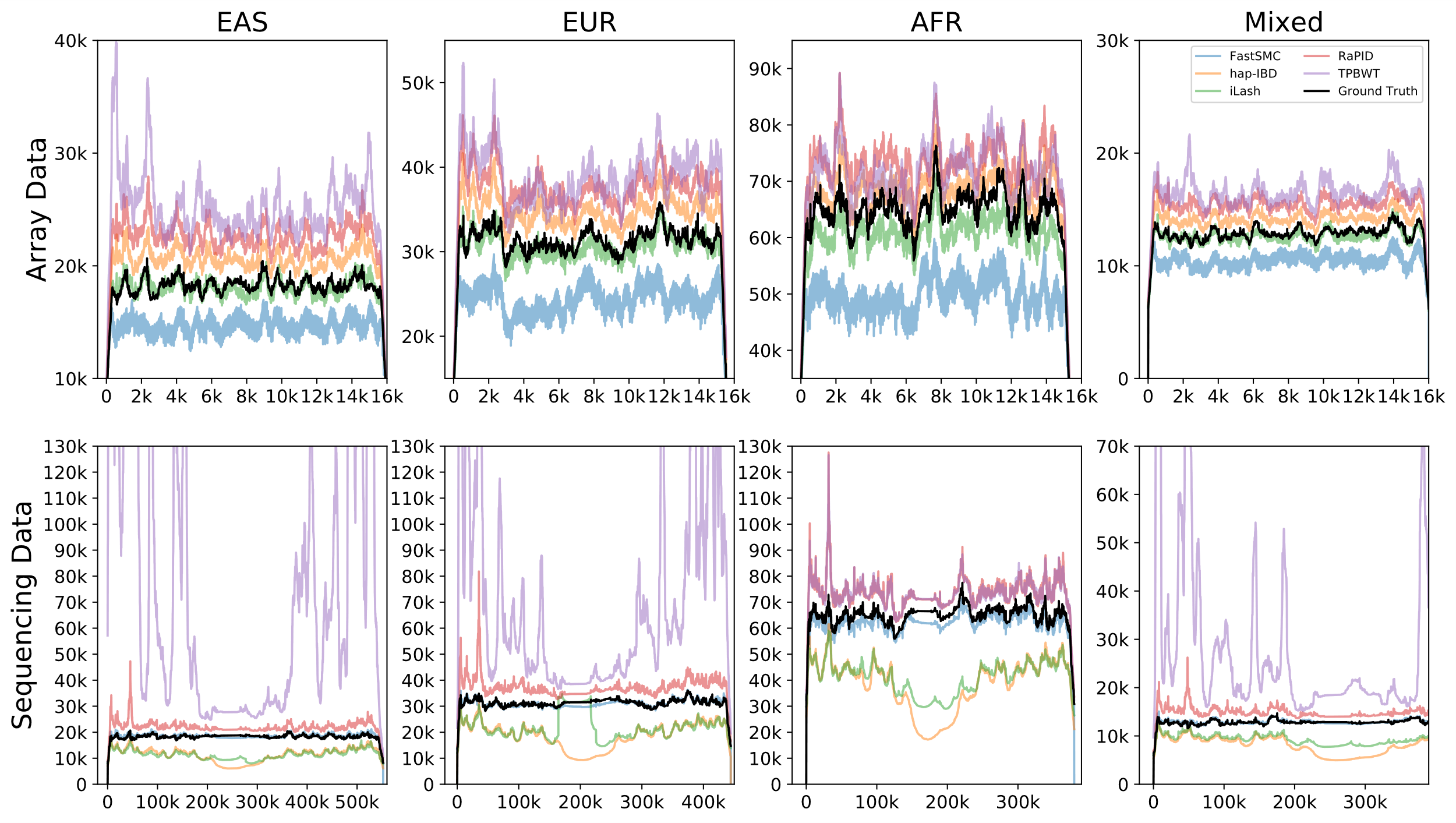


Supplementary Figure S3. IBD segment coverage in array and sequencing data with a genotyping error rate of 0.1% for three different populations. The x-axis represents the site index, and the y-axis denotes the number of IBD segments that covered each site. The ground truth is displayed in black.


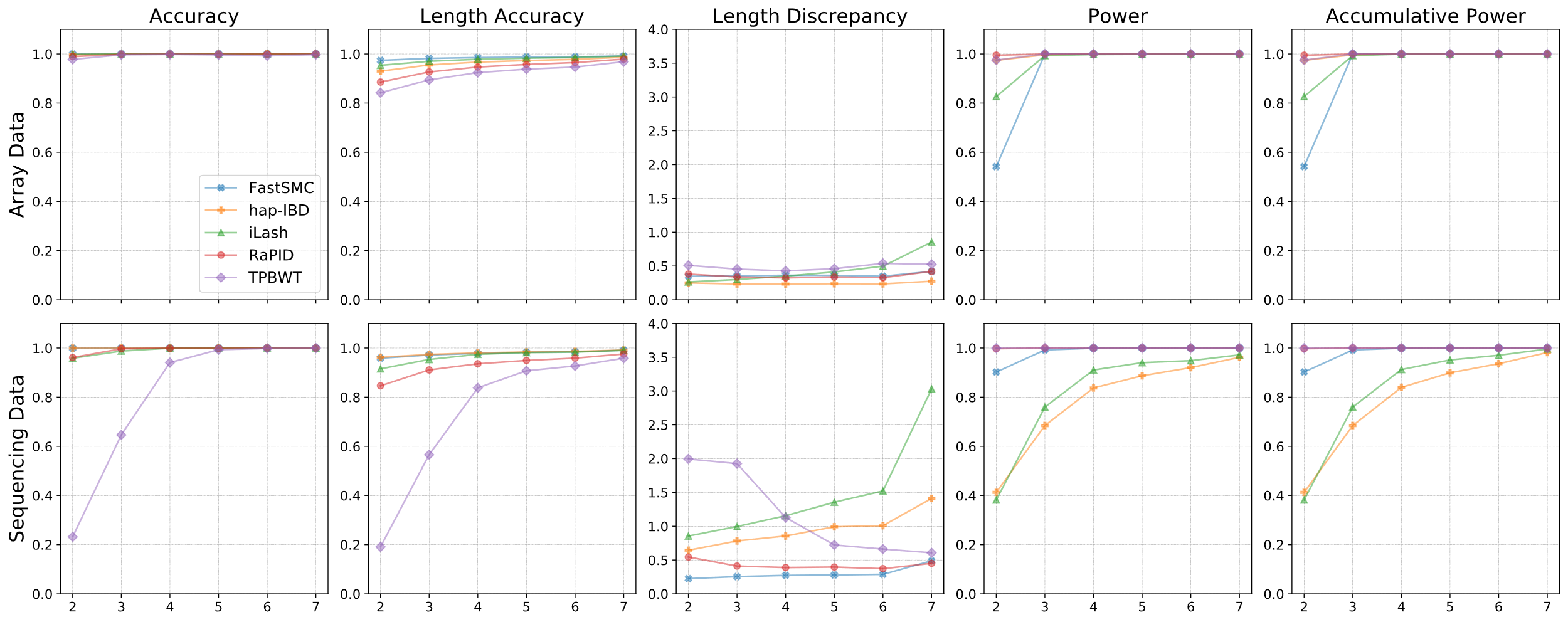


Supplementary Figure S4. Benchmarking results of different IBD detection tools in EUR array and sequencing data with a genotyping error rate of 0%. The length discrepancy is measured in cM.


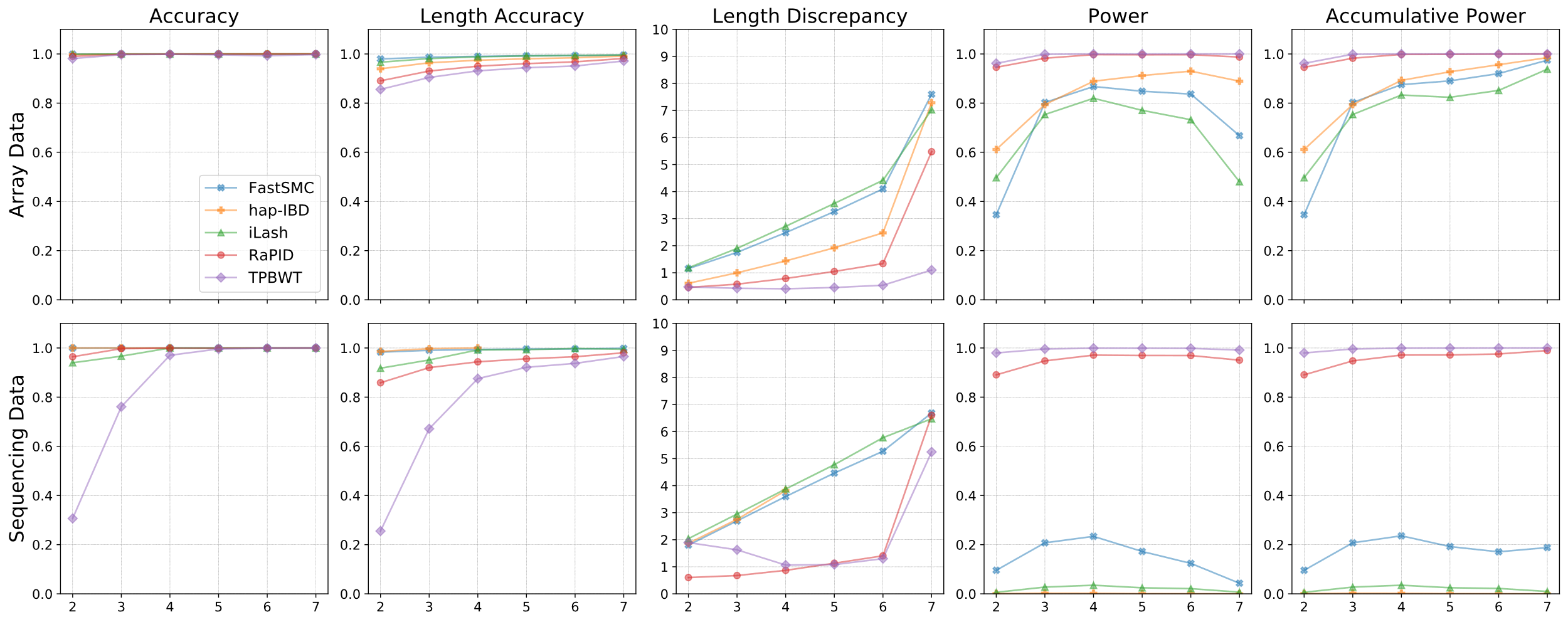


Supplementary Figure S5. Benchmarking results of different IBD detection tools in EUR array and sequencing data with a genotyping error rate of 0.2%. The length discrepancy is measured in cM.


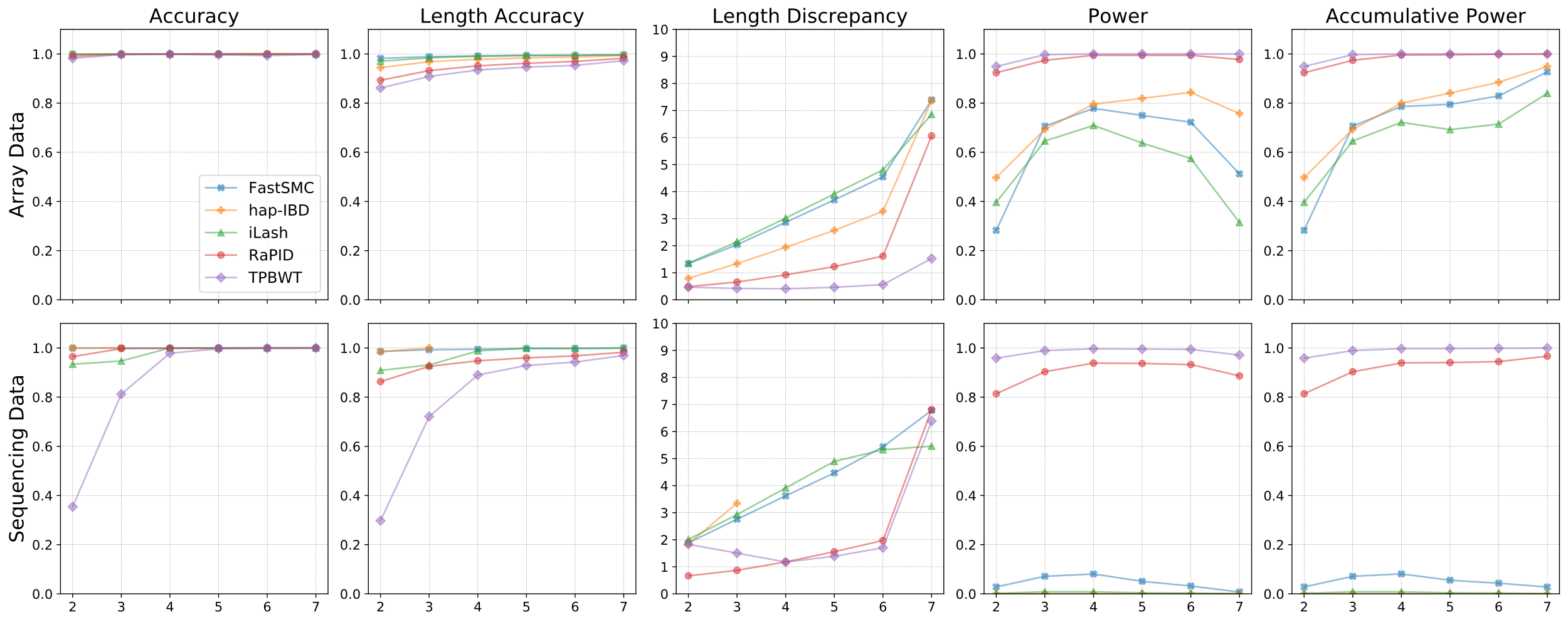
Supplementary Figure S6. Benchmarking results of different IBD detection tools in EUR array and sequencing data with a genotyping error rate of 0.3%. The length discrepancy is measured in cM.


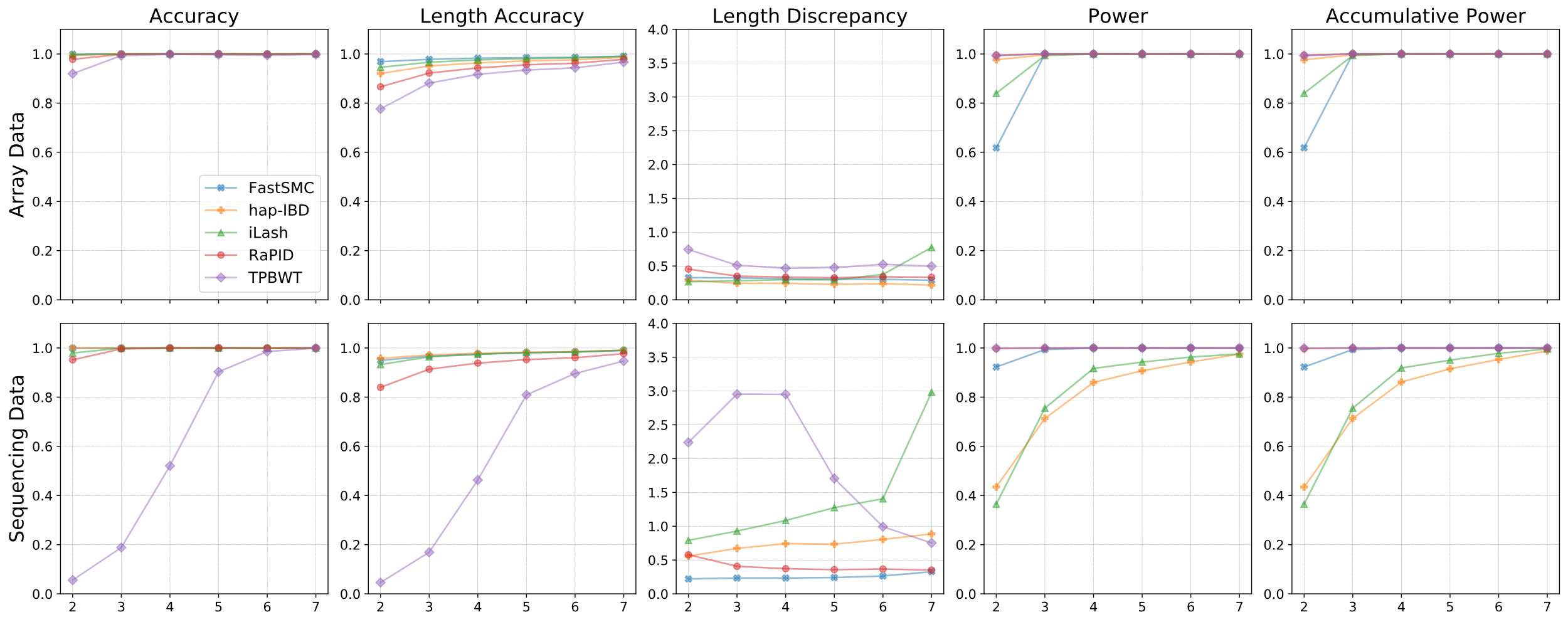


Supplementary Figure S7. Benchmarking results of different IBD detection tools in EAS array and sequencing data with a genotyping error rate of 0%. The length discrepancy is measured in cM.


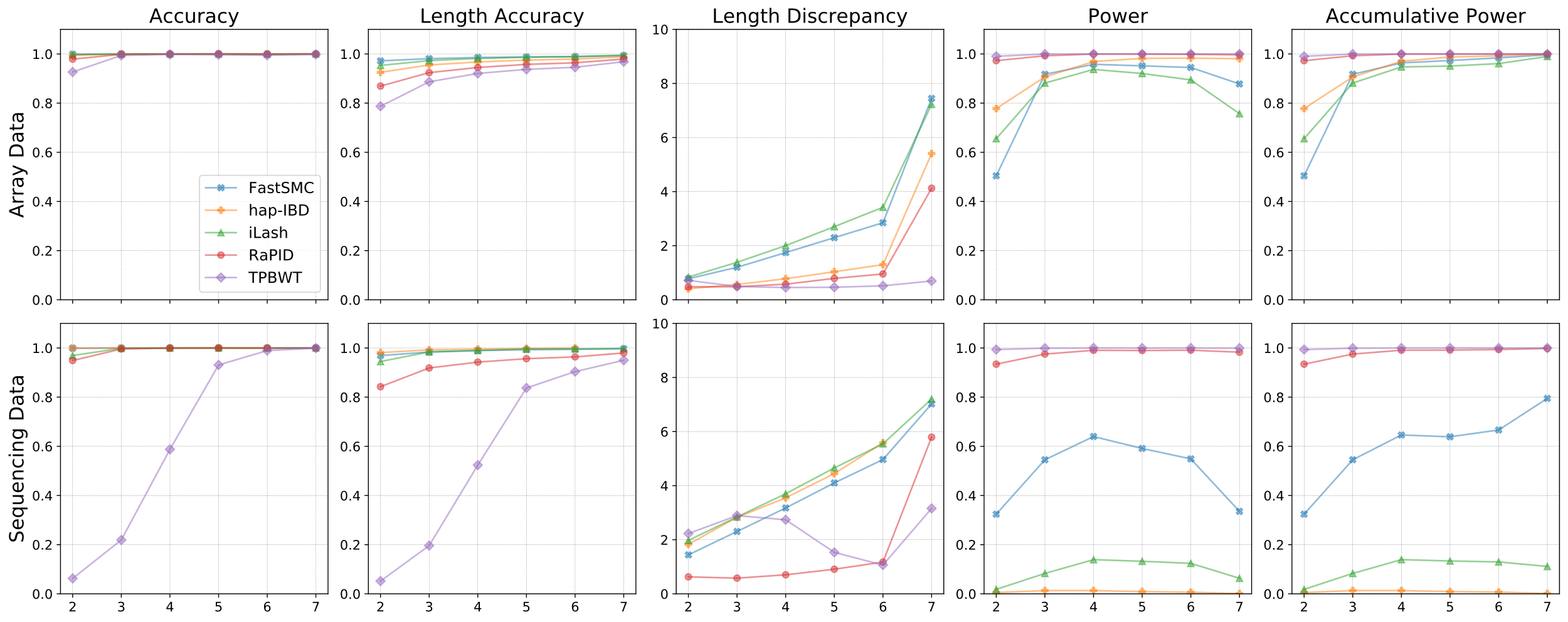


Supplementary Figure S8. Benchmarking results of different IBD detection tools in EAS array and sequencing data with a genotyping error rate of 0.1%. The length discrepancy is measured by cM.


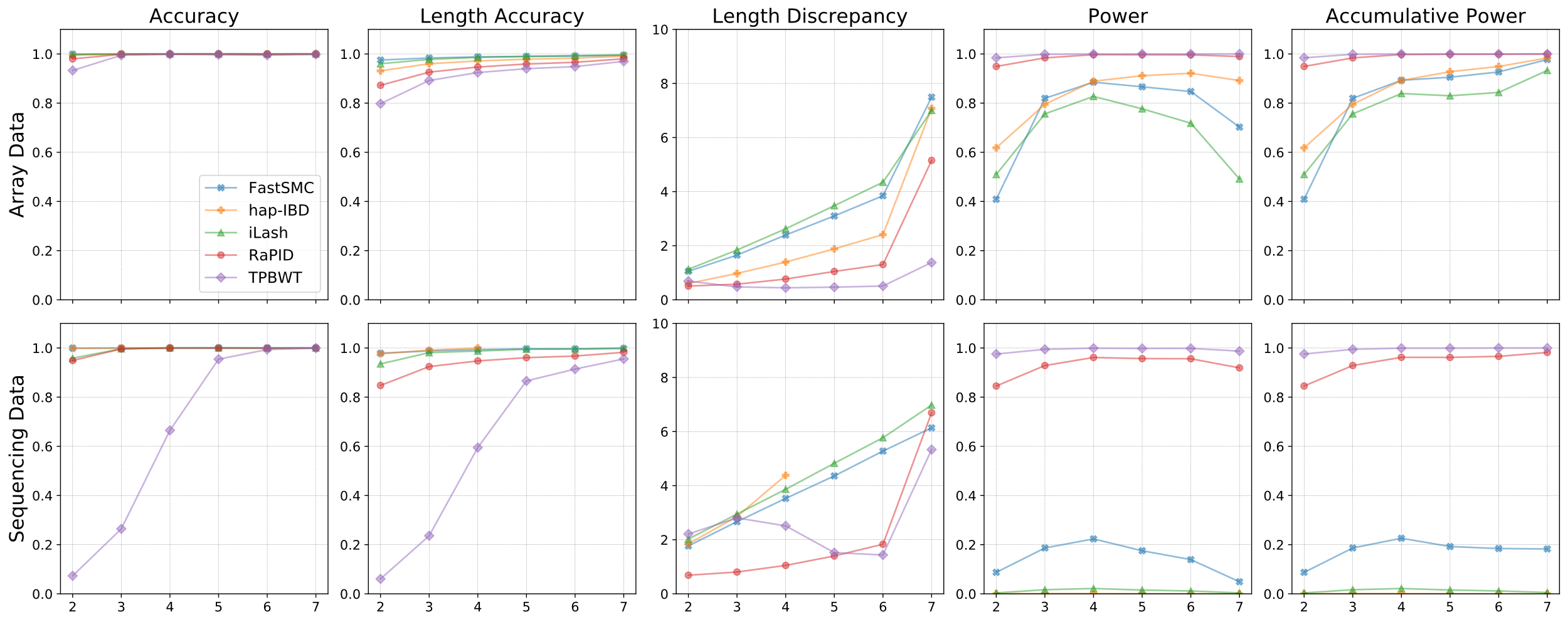


Supplementary Figure S9. Benchmarking results of different IBD detection tools in EAS array and sequencing data with a genotyping error rate of 0.2%. The length discrepancy is measured in cM.


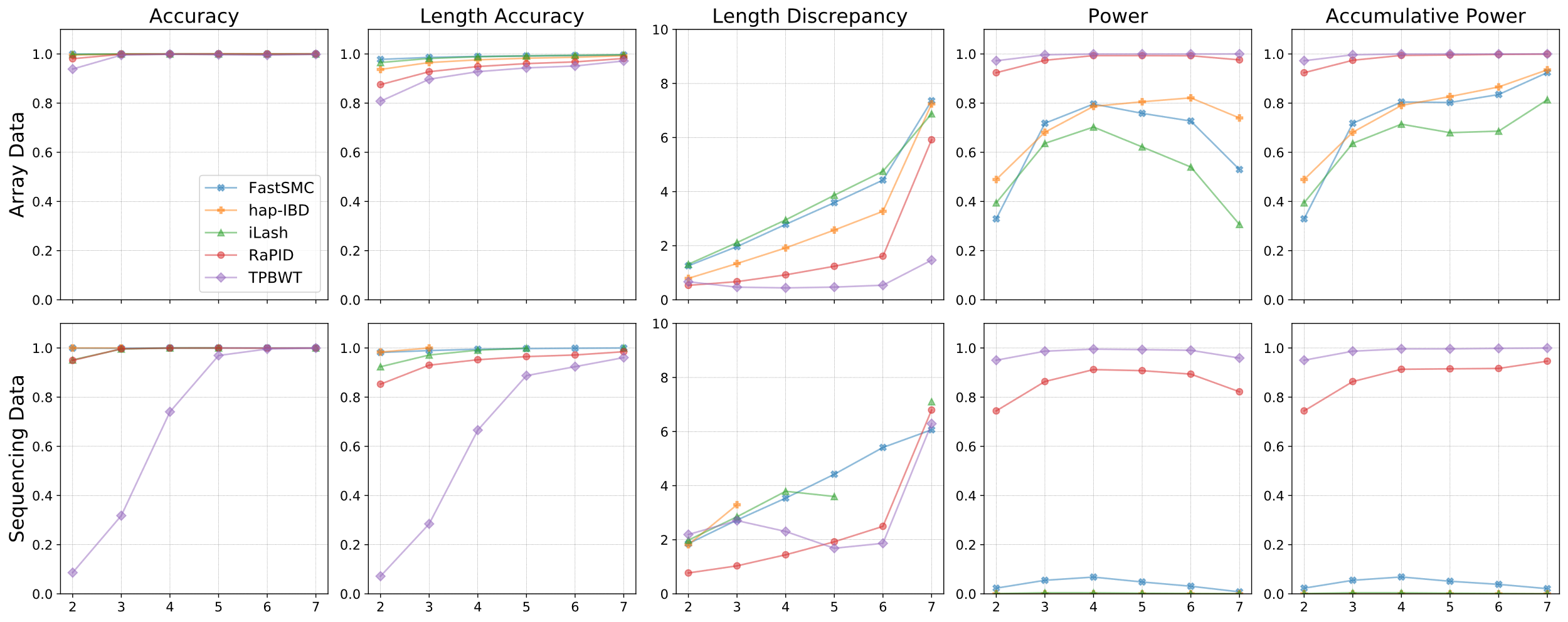


Supplementary Figure S10. Benchmarking results of different IBD detection tools in EAS array and sequencing data with a genotyping error rate of 0.3%. The length discrepancy is measured in cM.


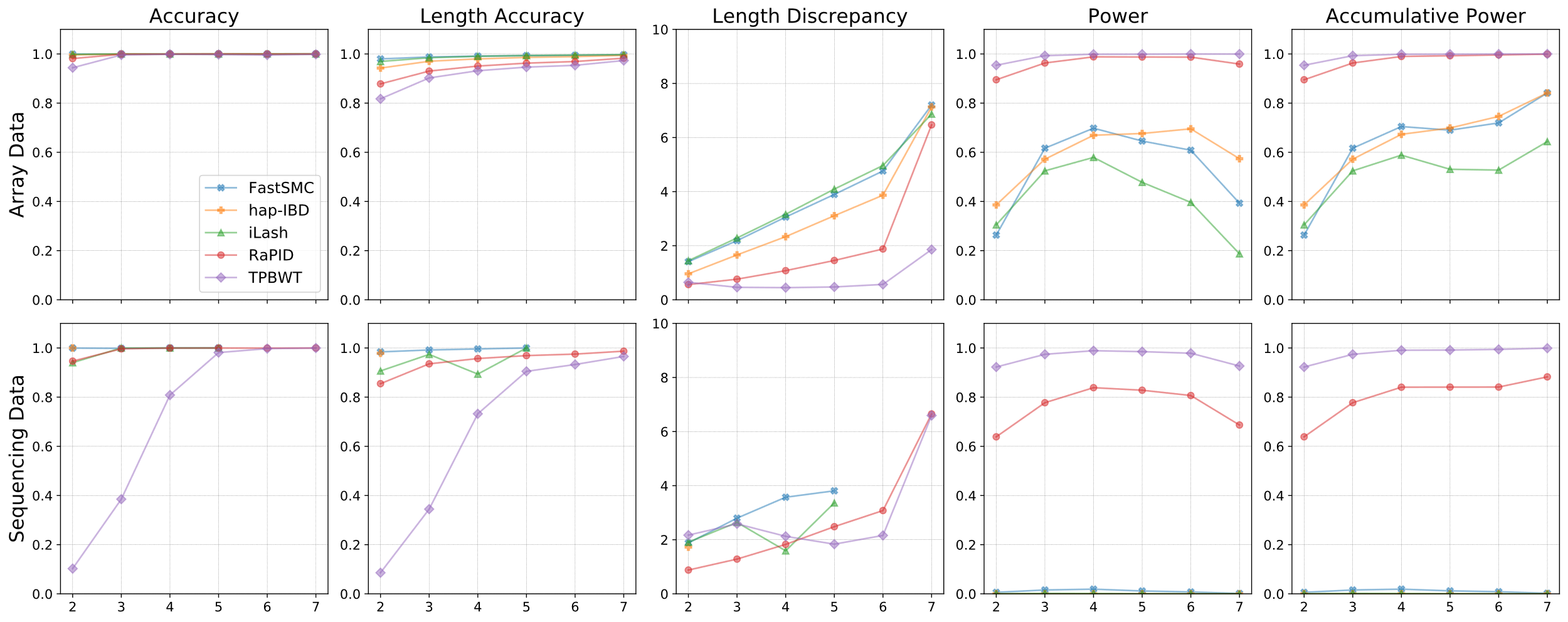


Supplementary Figure S11. Benchmarking results of different IBD detection tools in EAS array and sequencing data with a genotyping error rate of 0.4%. The length discrepancy is measured by cM.


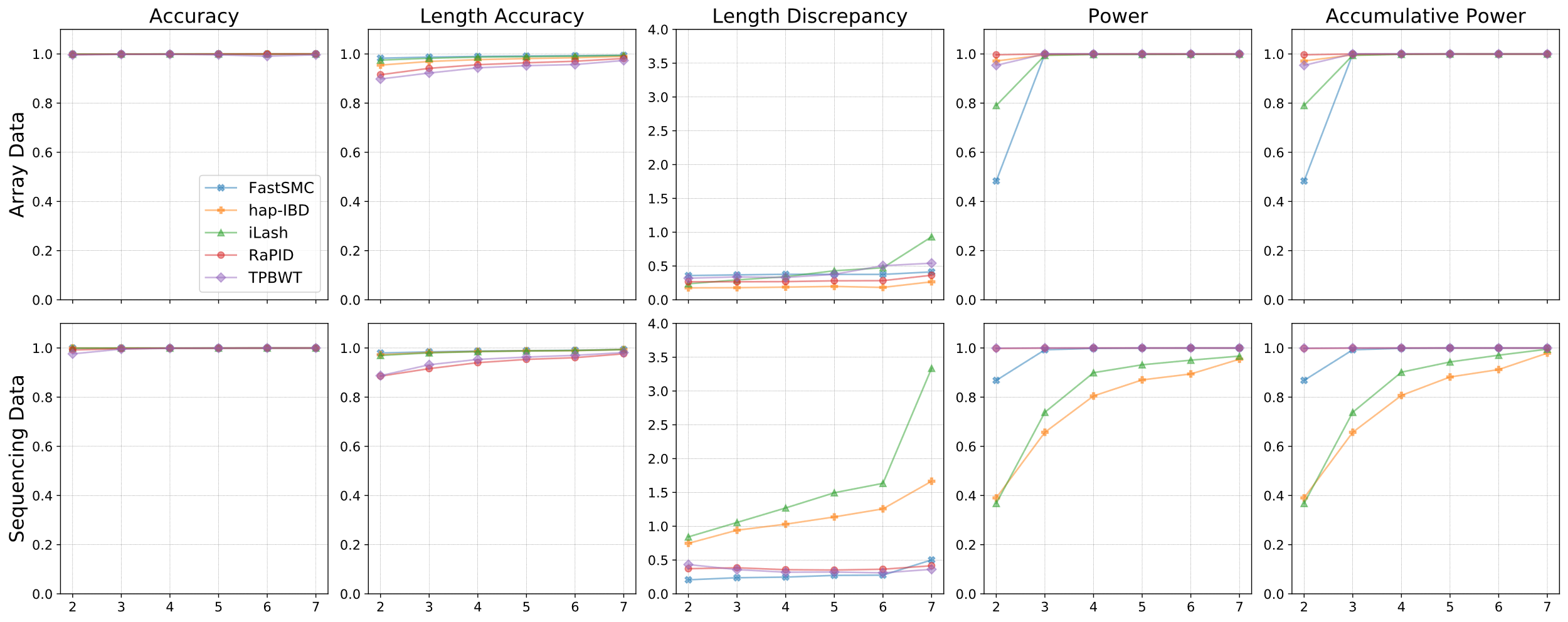


Supplementary Figure S12. Benchmarking results of different IBD detection tools in AFR array and sequencing data with a genotyping error rate of 0%. The length discrepancy is measured by cM.


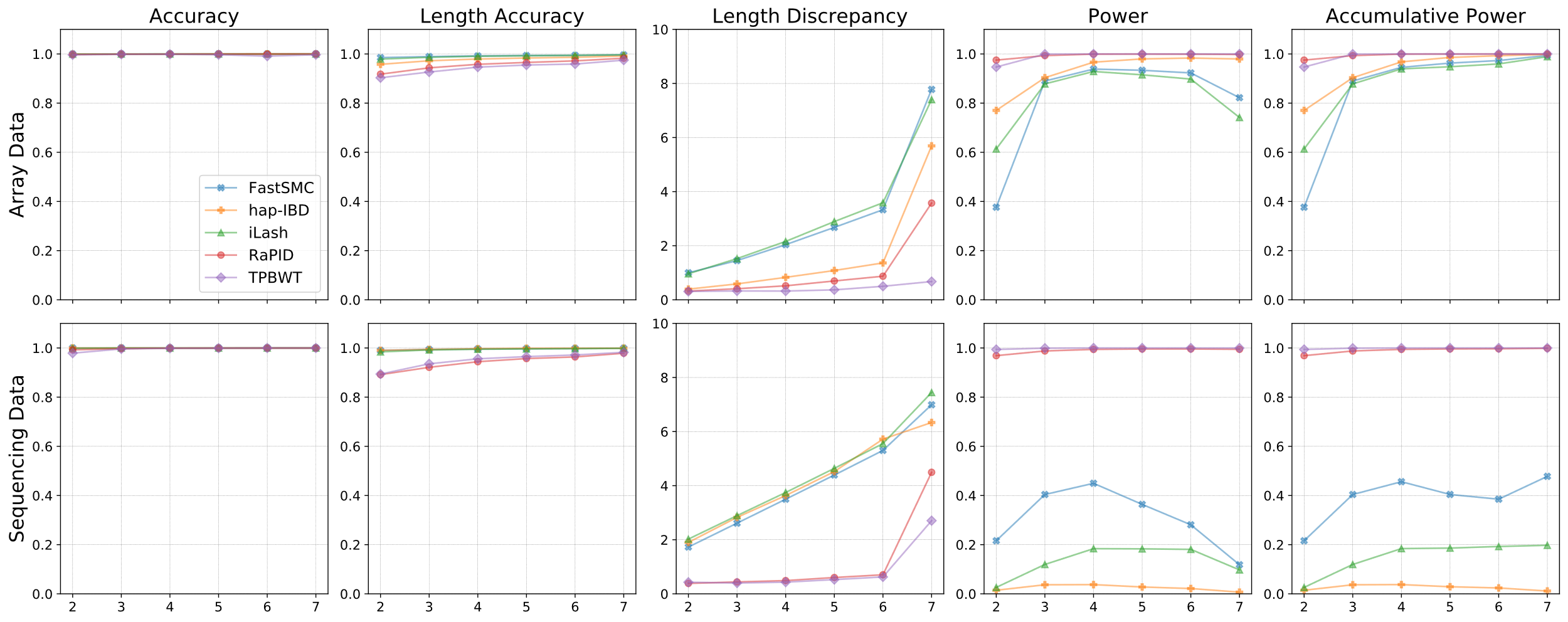


Supplementary Figure S13. Benchmarking results of different IBD detection tools in AFR array and sequencing data with a genotyping error rate of 0.1%. The length discrepancy is measured by cM.


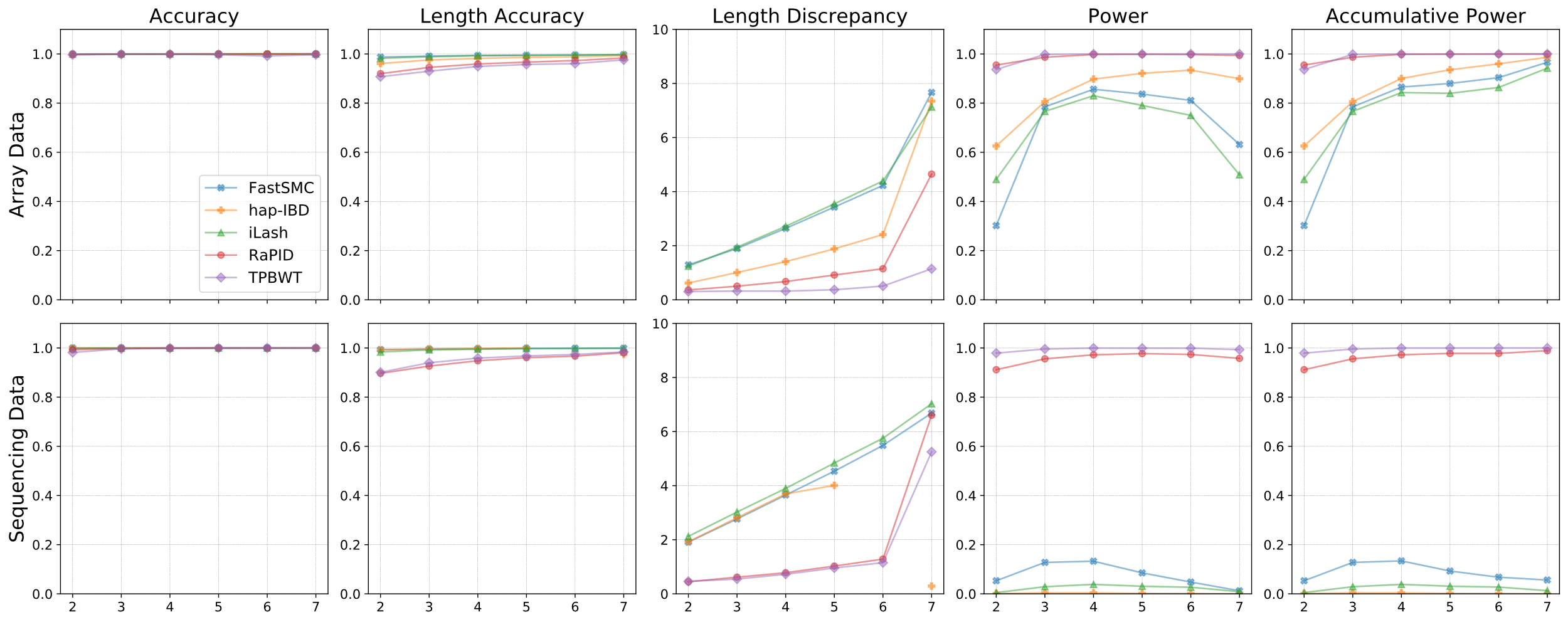


Supplementary Figure S14. Benchmarking results of different IBD detection tools in AFR array and sequencing data with a genotyping error rate of 0.2%. The length discrepancy is measured by cM. Some lines may be discontinued or dipped due to low power.


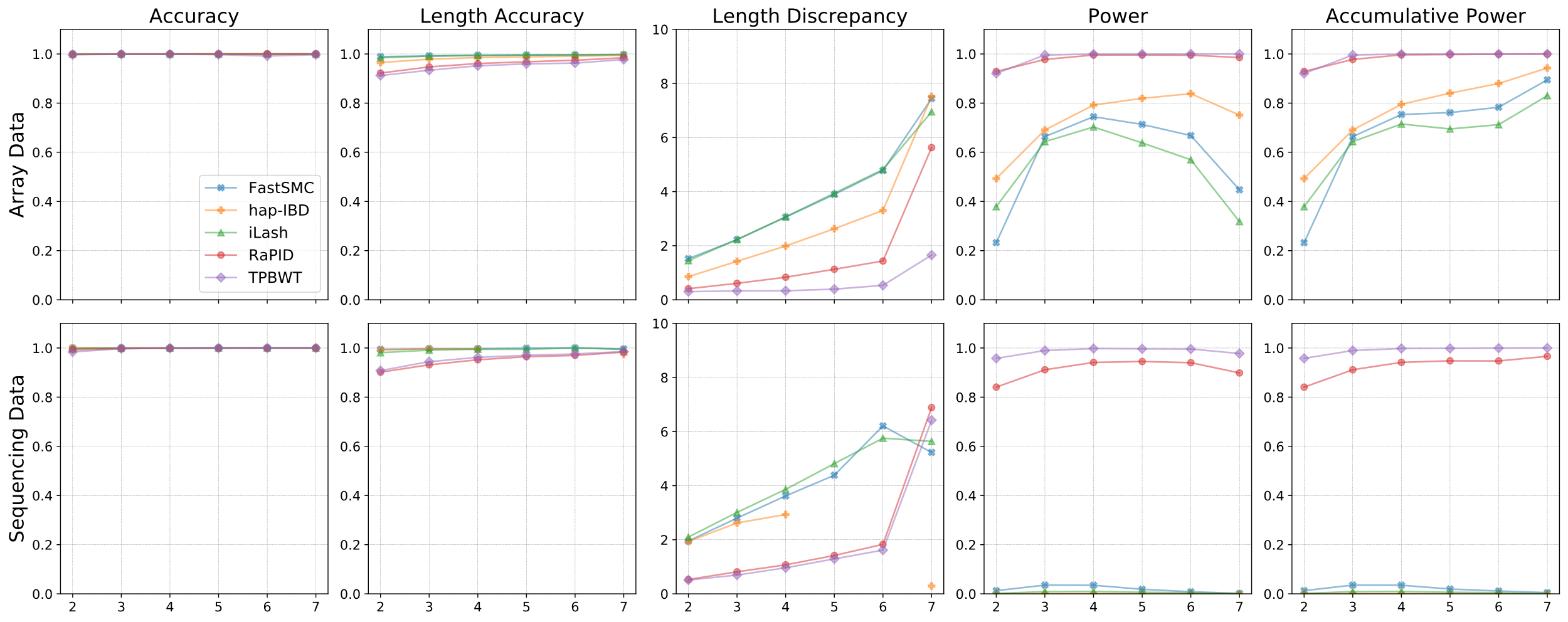


Supplementary Figure S15. Benchmarking results of different IBD detection tools in AFR array and sequencing data with a genotyping error rate of 0.3%. The length discrepancy is measured by cM, other measures are based on percentage. Some lines may be discontinued or dipped due to low power.


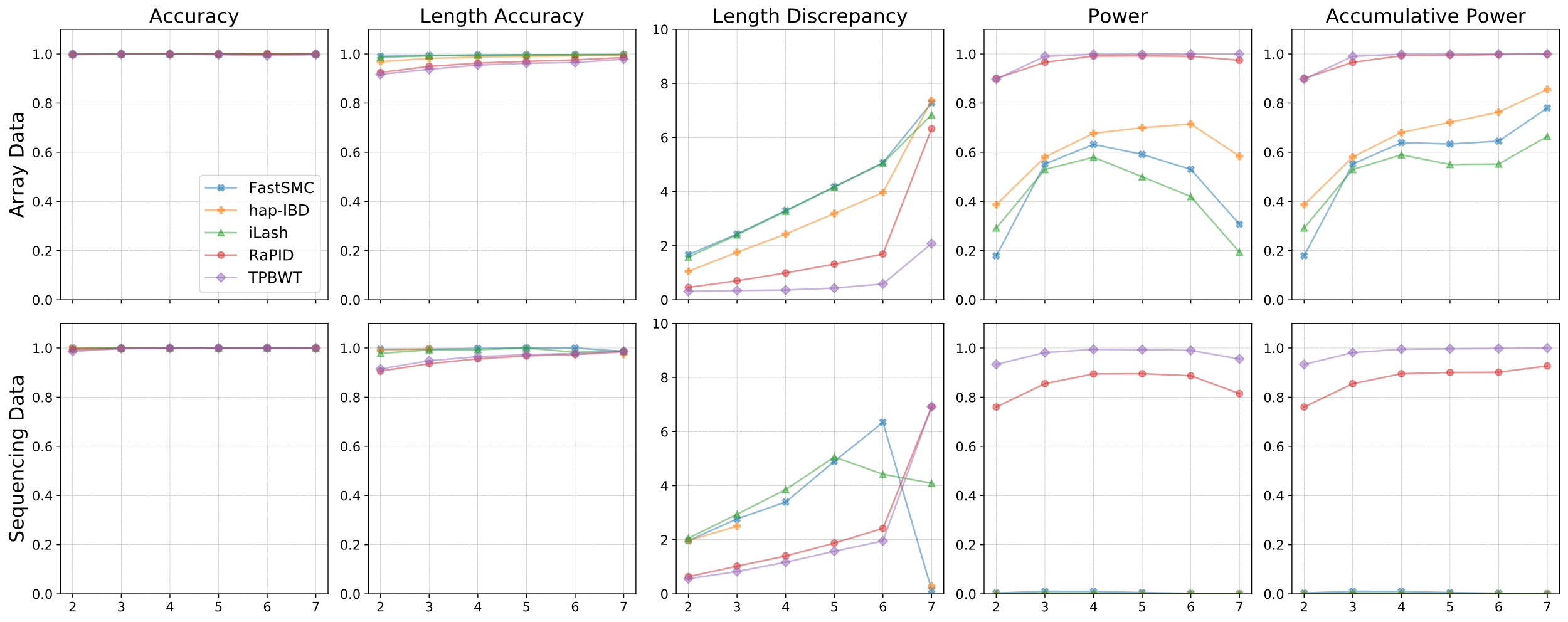


Supplementary Figure S16. Benchmarking results of different IBD detection tools in AFR array and sequencing data with a genotyping error rate of 0.4%. The length discrepancy is measured by cM, other measures are based on percentage. Some lines may be discontinued or dipped due to low power.


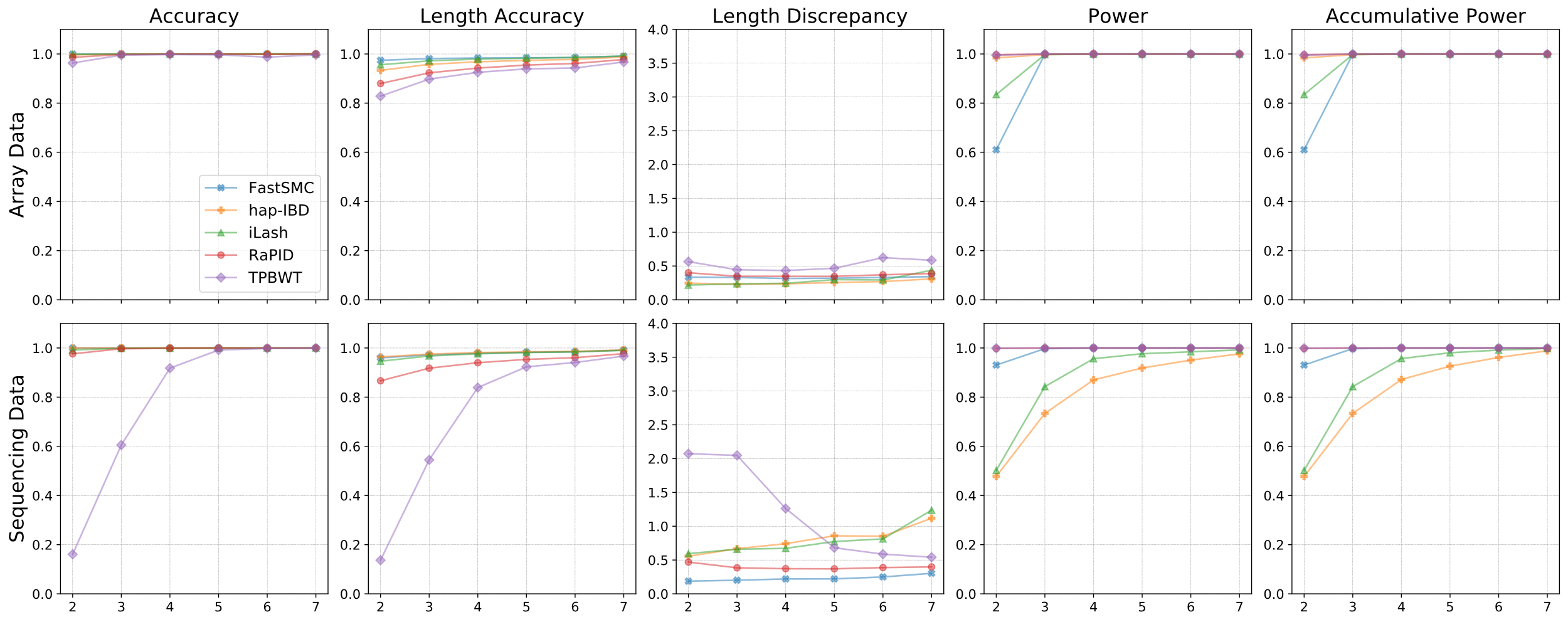


Supplementary Figure S17. Benchmarking results of different IBD detection tools in Mixed array and sequencing data without genotyping. The length discrepancy is measured by cM, other measures are based on percentage.


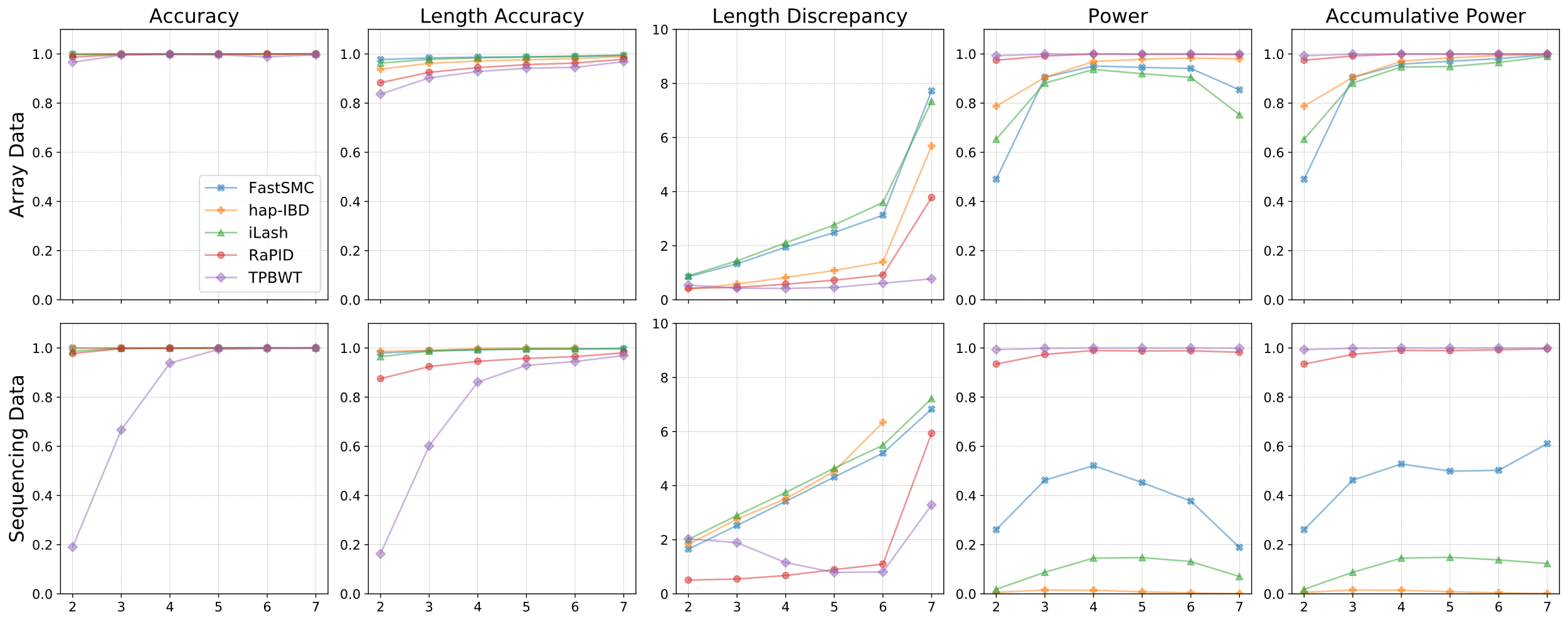


Supplementary Figure S18. Benchmarking results of different IBD detection tools in Mixed array and sequencing data with a genotyping error rate of 0.1%. The length discrepancy is measured by cM, other measures are based on percentage. Some lines may be discontinued or dipped due to low power.


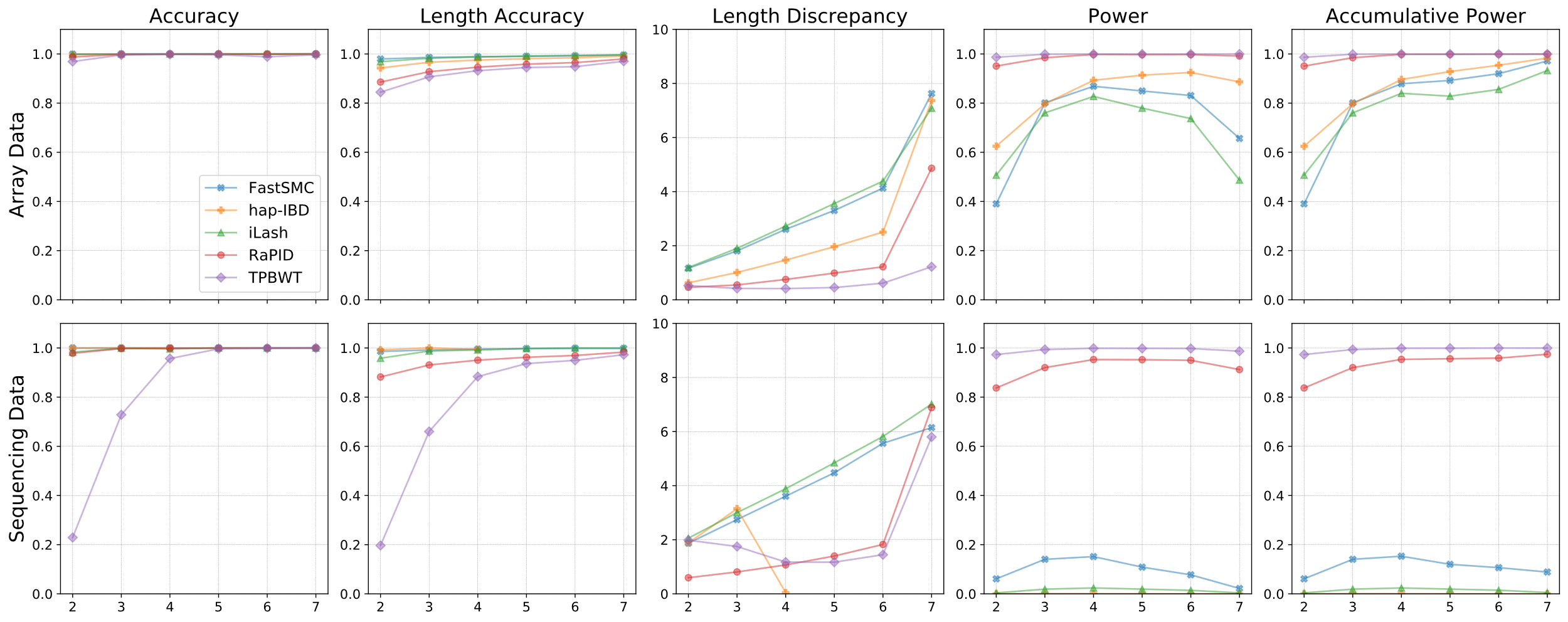


Supplementary Figure S19. Benchmarking results of different IBD detection tools in Mixed array and sequencing data with a genotyping error rate of 0.2%. The length discrepancy is measured by cM, other measures are based on percentage. Some lines may be discontinued or dipped due to low power.


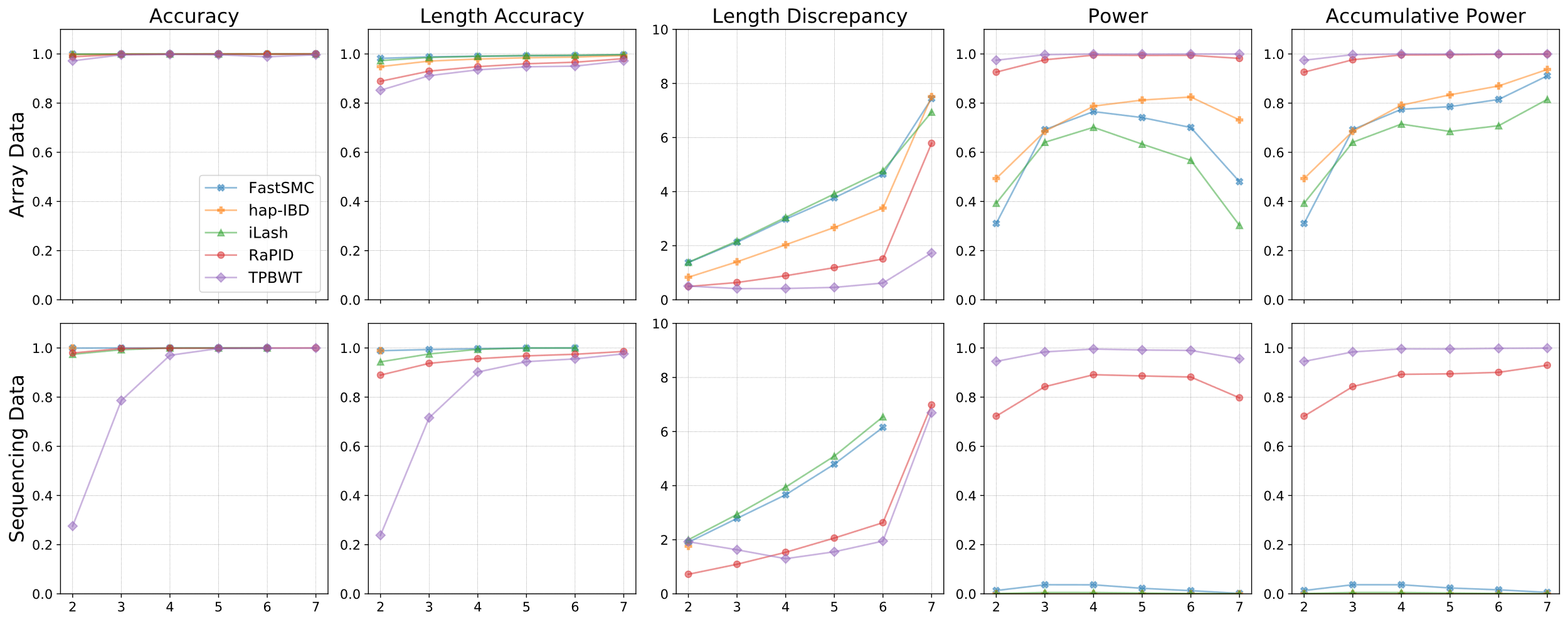


Supplementary Figure S20. Benchmarking results of different IBD detection tools in Mixed array and sequencing data with a genotyping error rate of 0.3%. The length discrepancy is measured by cM, other measures are based on percentage. Some lines may be discontinued or dipped due to low power.


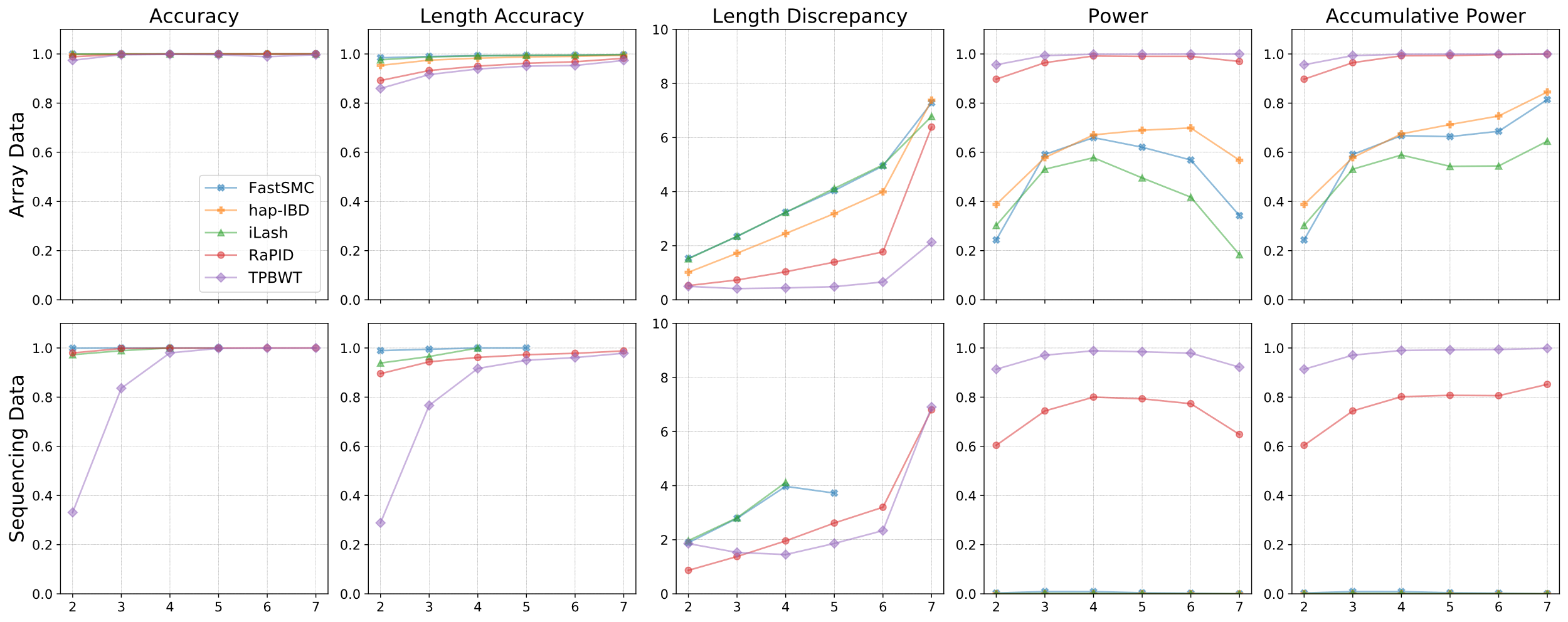


Supplementary Figure S21. Benchmarking results of different IBD detection tools in Mixed array and sequencing data with a genotyping error rate of 0.4%. The length discrepancy is measured by cM, other measures are based on percentage. Some lines may be discontinued or dipped due to low power.


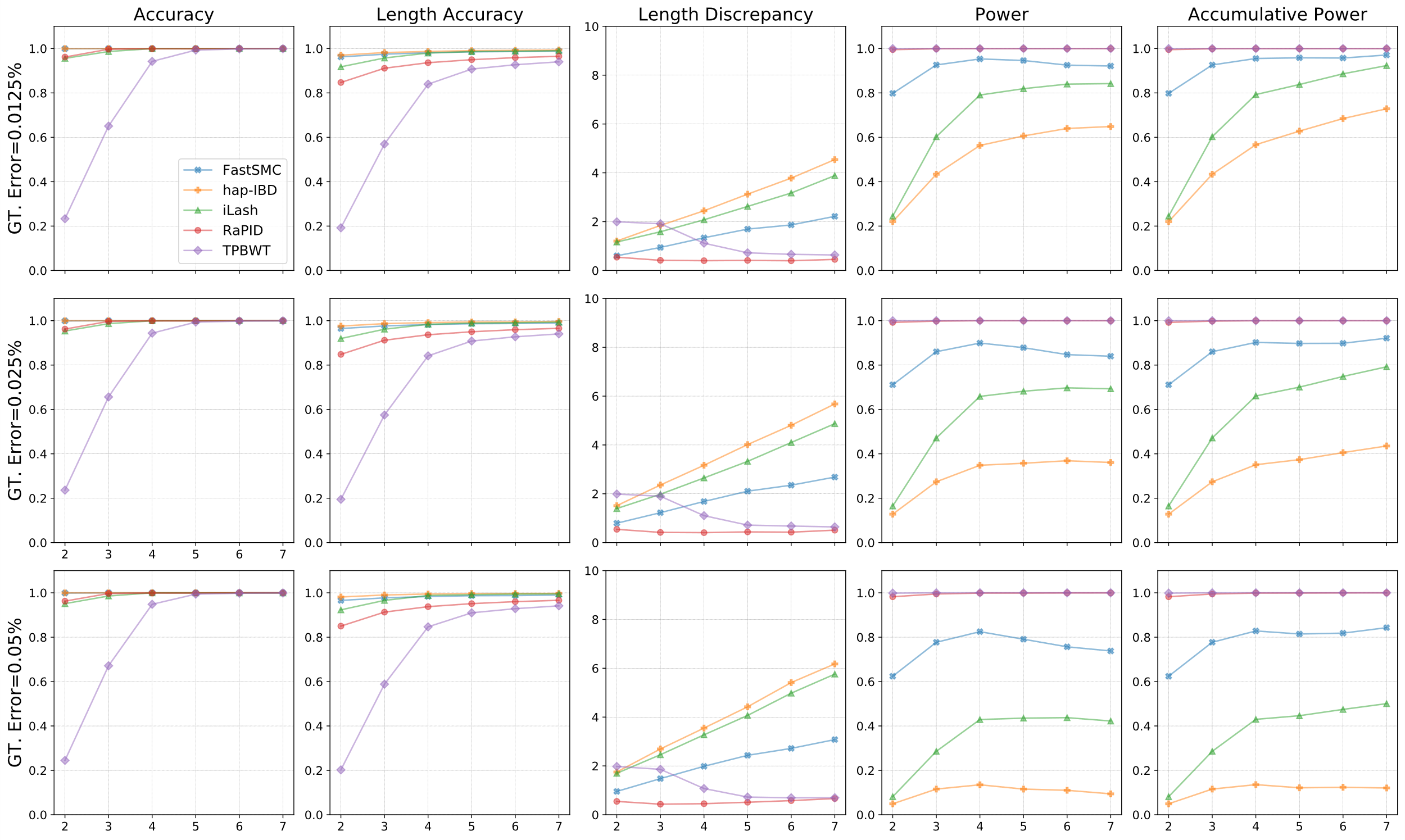
 Supplementary Figure S22. Benchmarking results of different tools in EUR sequencing data with low genotyping error rates.


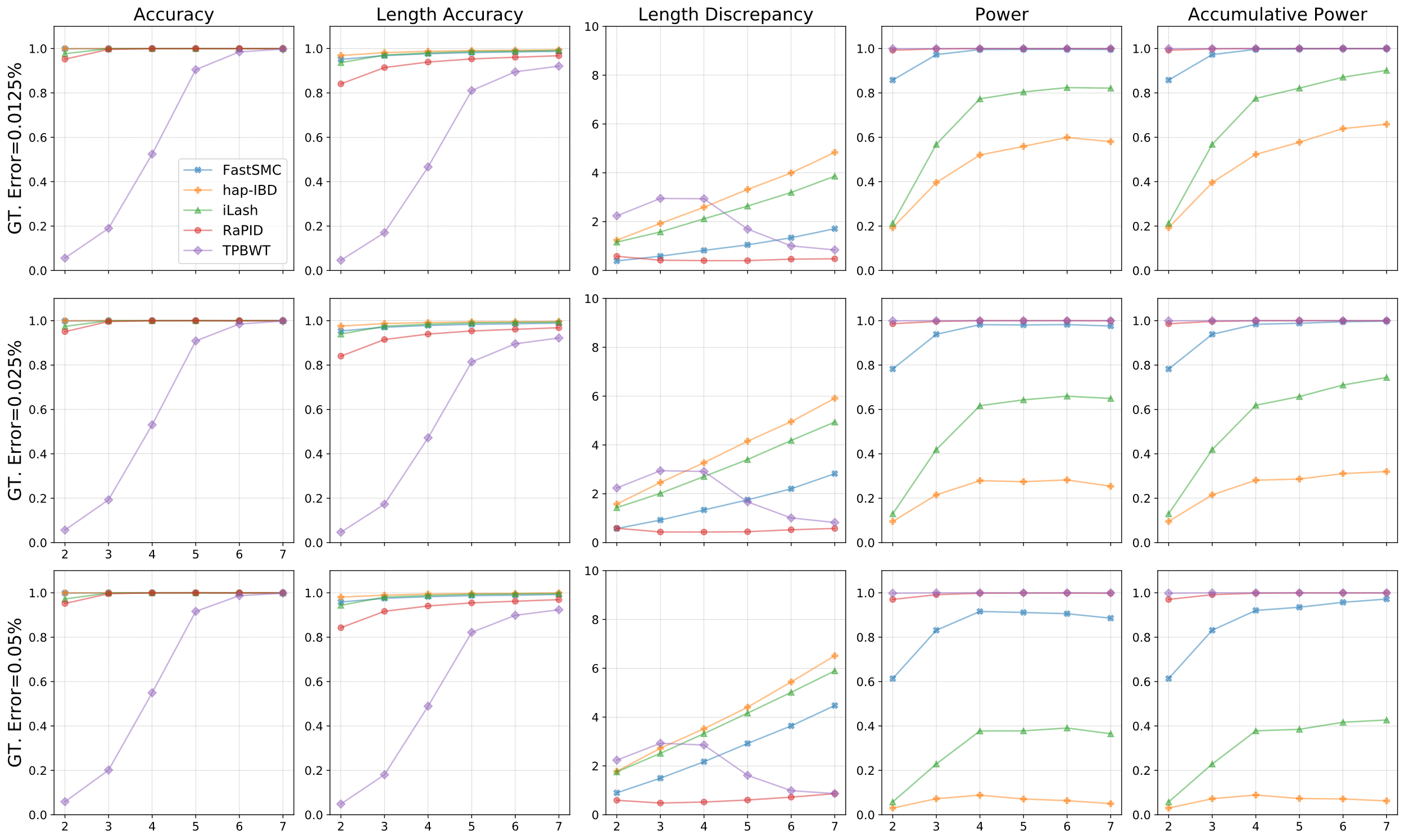
Supplementary Figure S23. Benchmarking results of different tools in EAS sequencing data with low genotyping error rates.


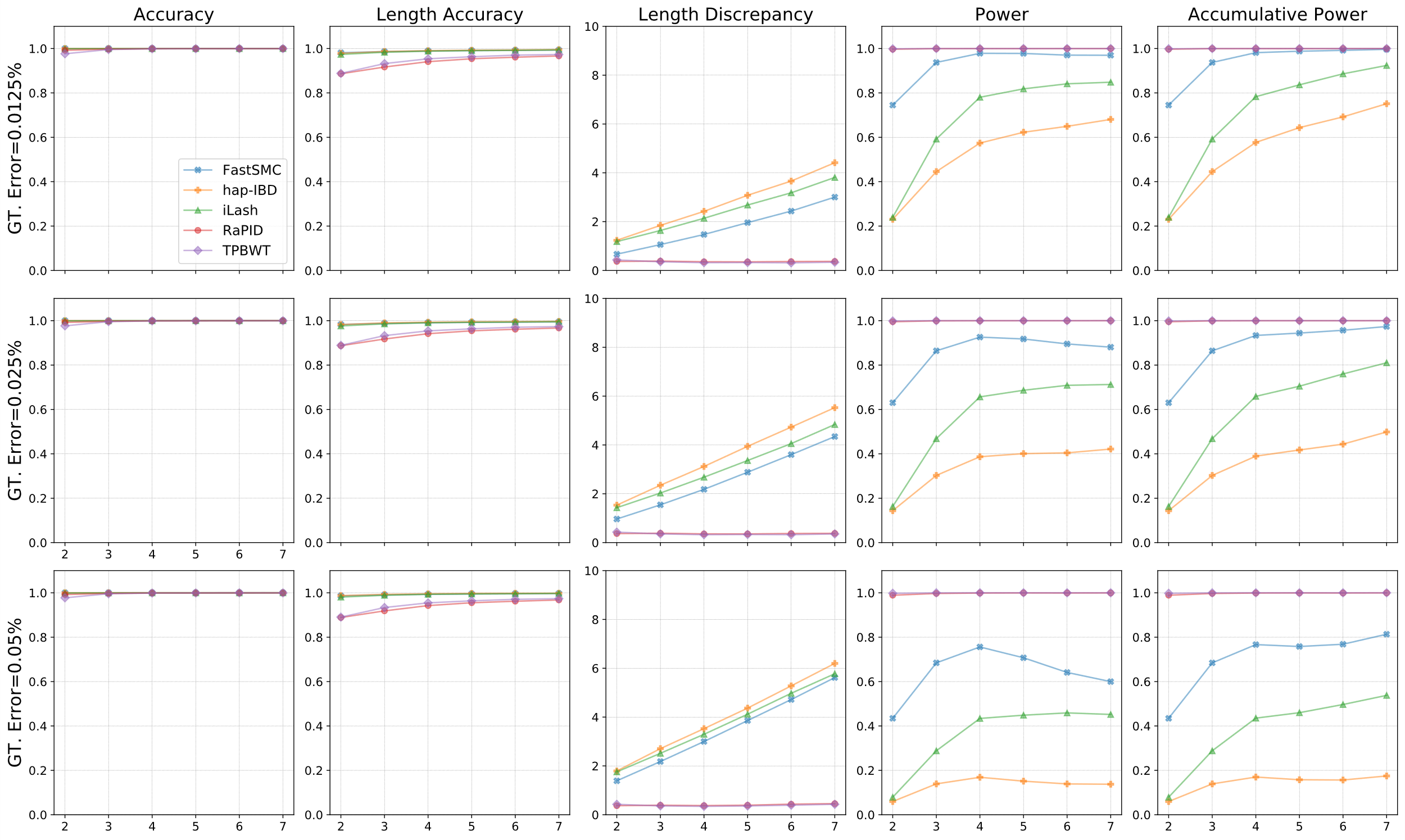


Supplementary Figure S24. Benchmarking results of different tools in AFR sequencing data with low genotyping error rates.


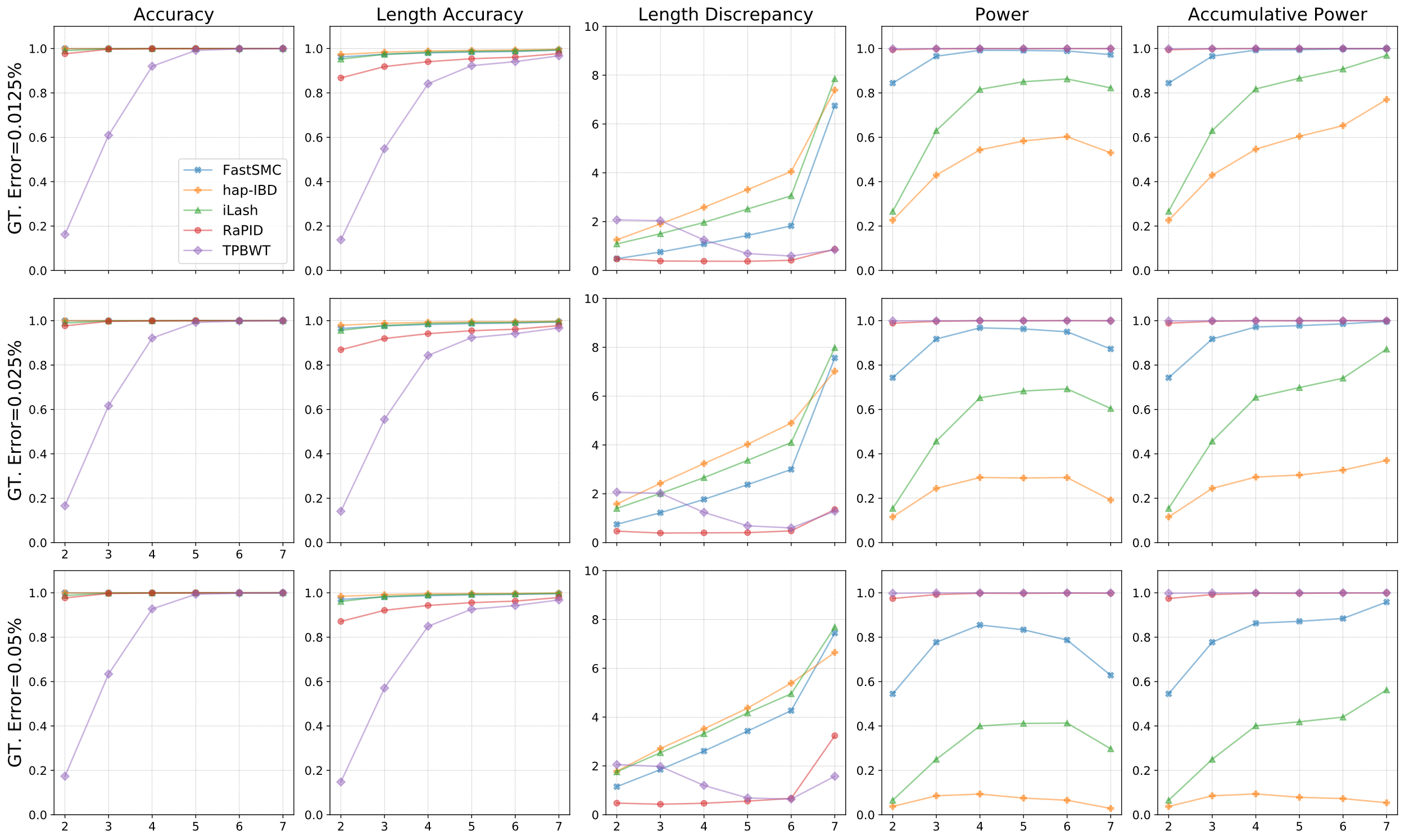


Supplementary Figure S25. Benchmarking results of different tools in Mixed sequencing data with low genotyping error rates.


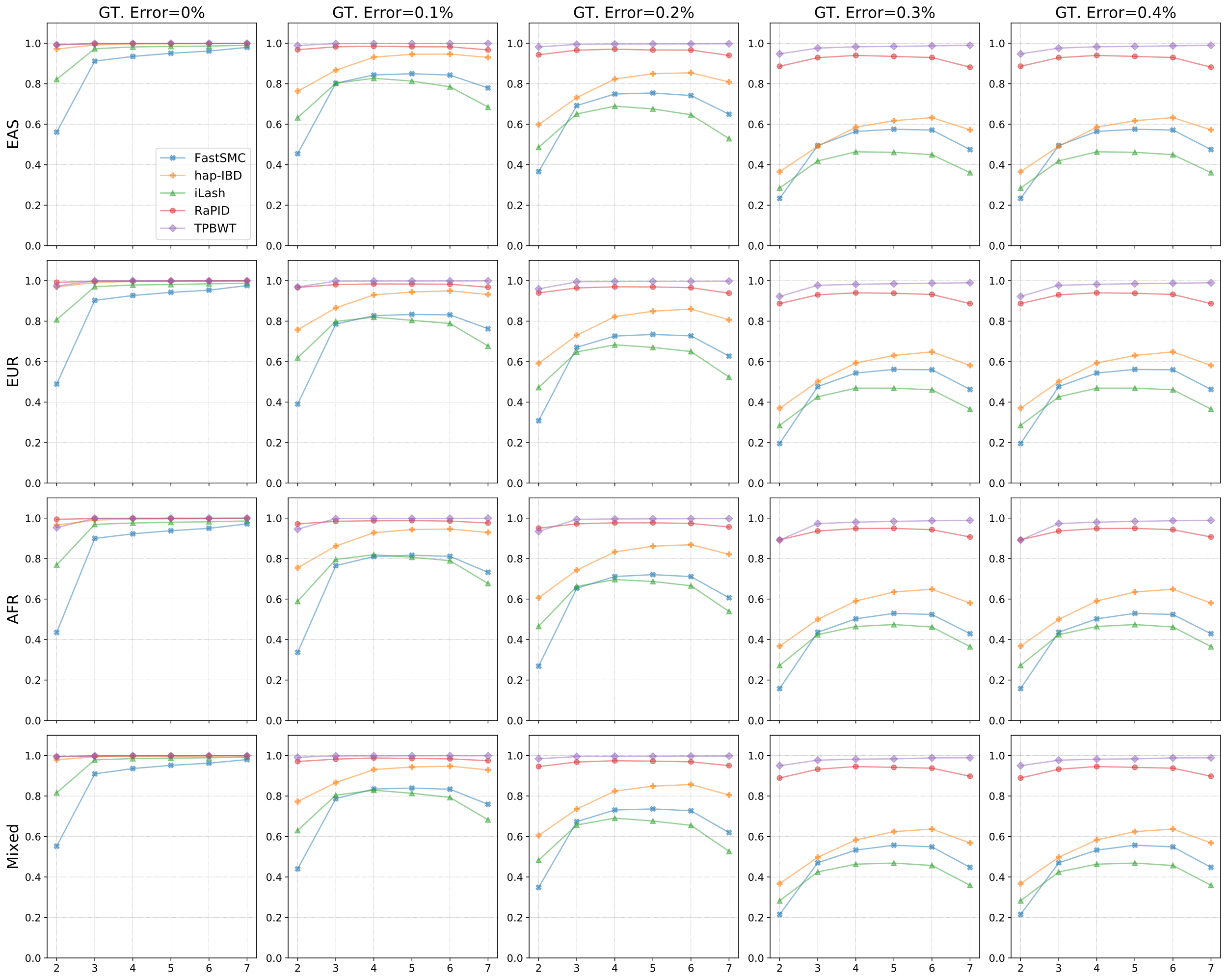


Supplementary Figure S26. IBD Recall of array datasets. Genotyping errors can affect the recall. X-axis in each subplot denotes the IBD length cut-off. TPBWT and RaPID’s recall values are not impacted significantly.


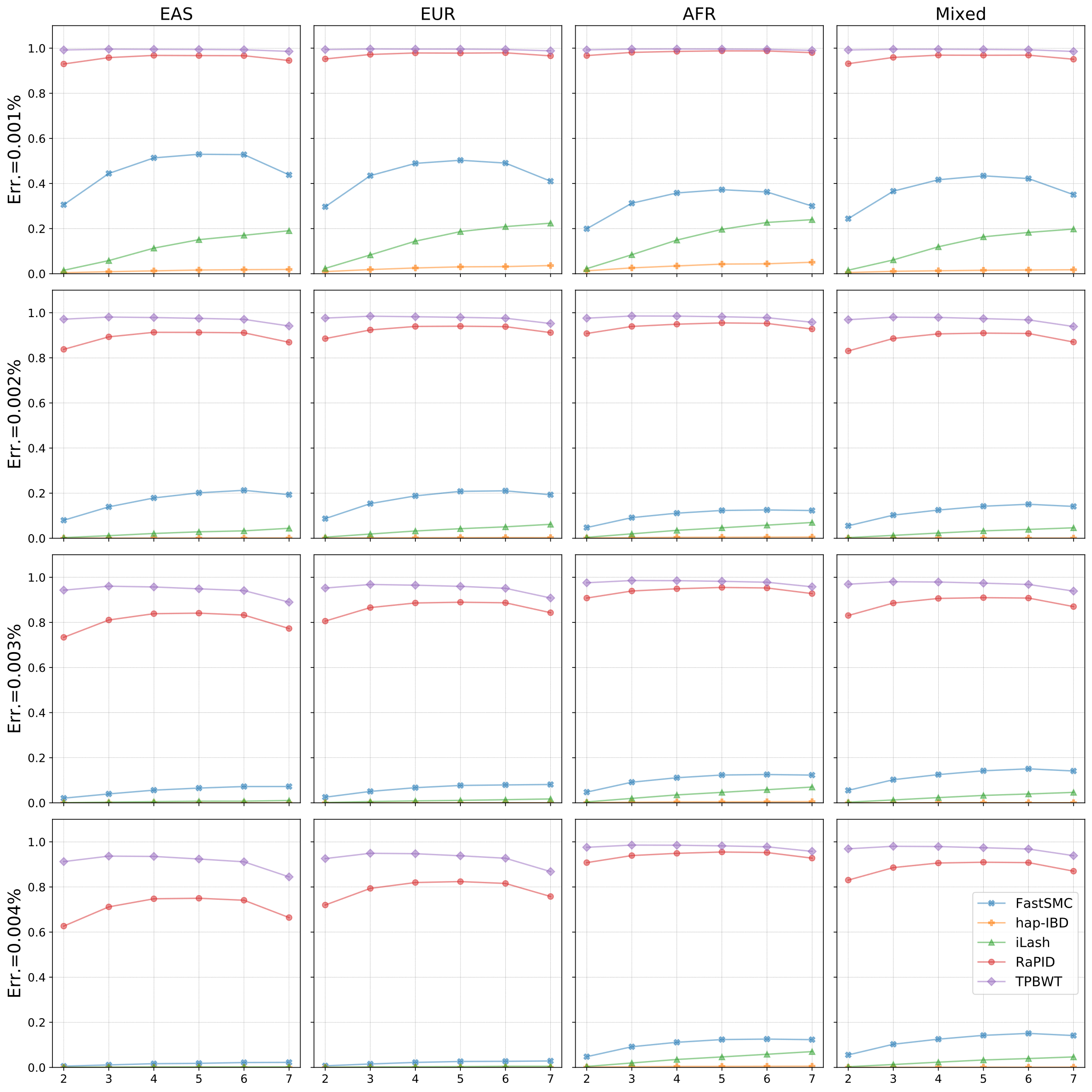


Supplementary Figure S27. IBD Recall of sequencing dataset with low genotyping error rates. Genotyping errors can affect the recall. X-axis in each subplot denotes the IBD length cut-off. TPBWT and RaPID’s recall values are not impacted significantly.


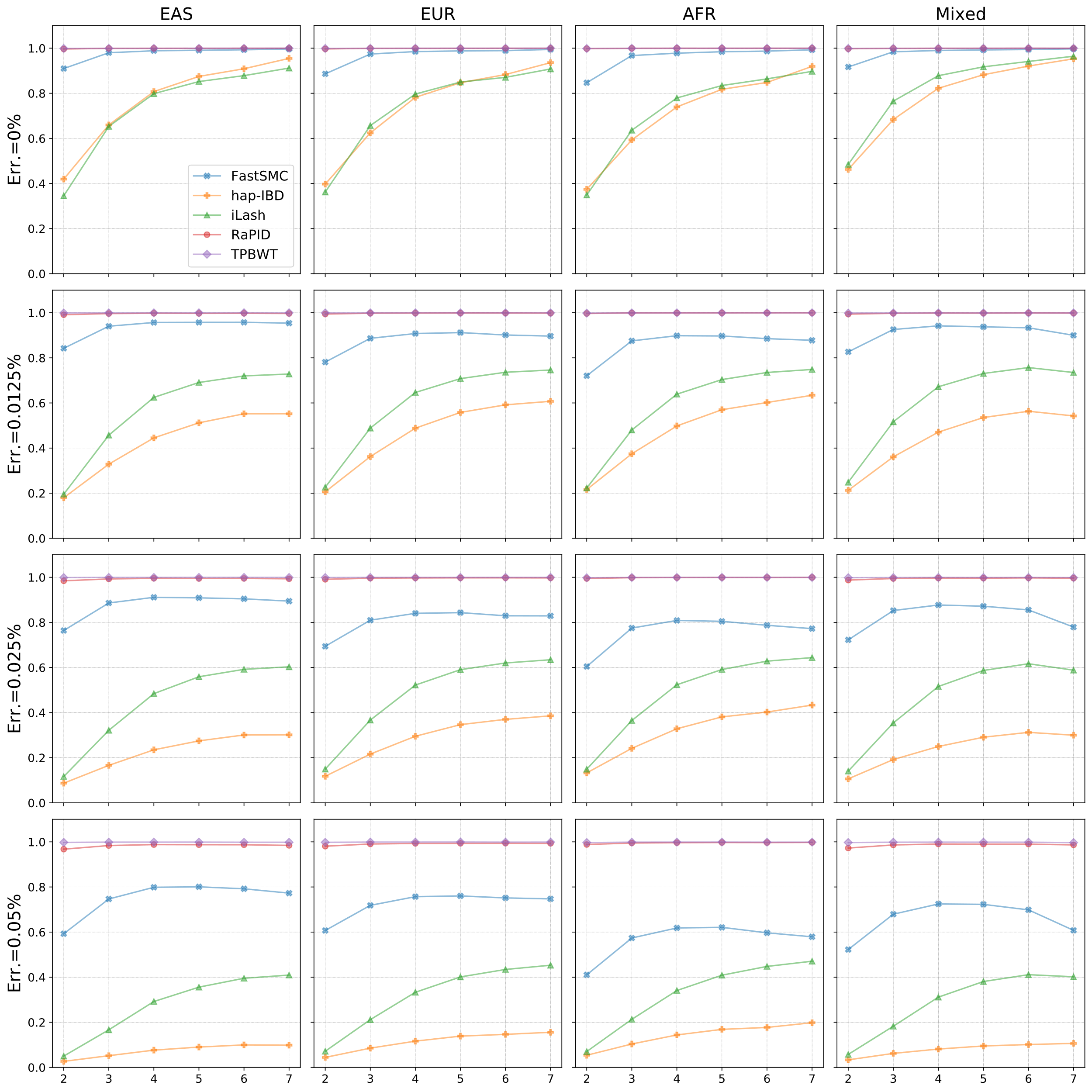


Supplementary Figure S28. IBD Recall of sequencing dataset with high genotyping error rates (≥0.1%). Genotyping errors can affect the recall. X-axis in each subplot denotes the IBD length cut-off. TPBWT and RaPID’s recall values are not impacted significantly.


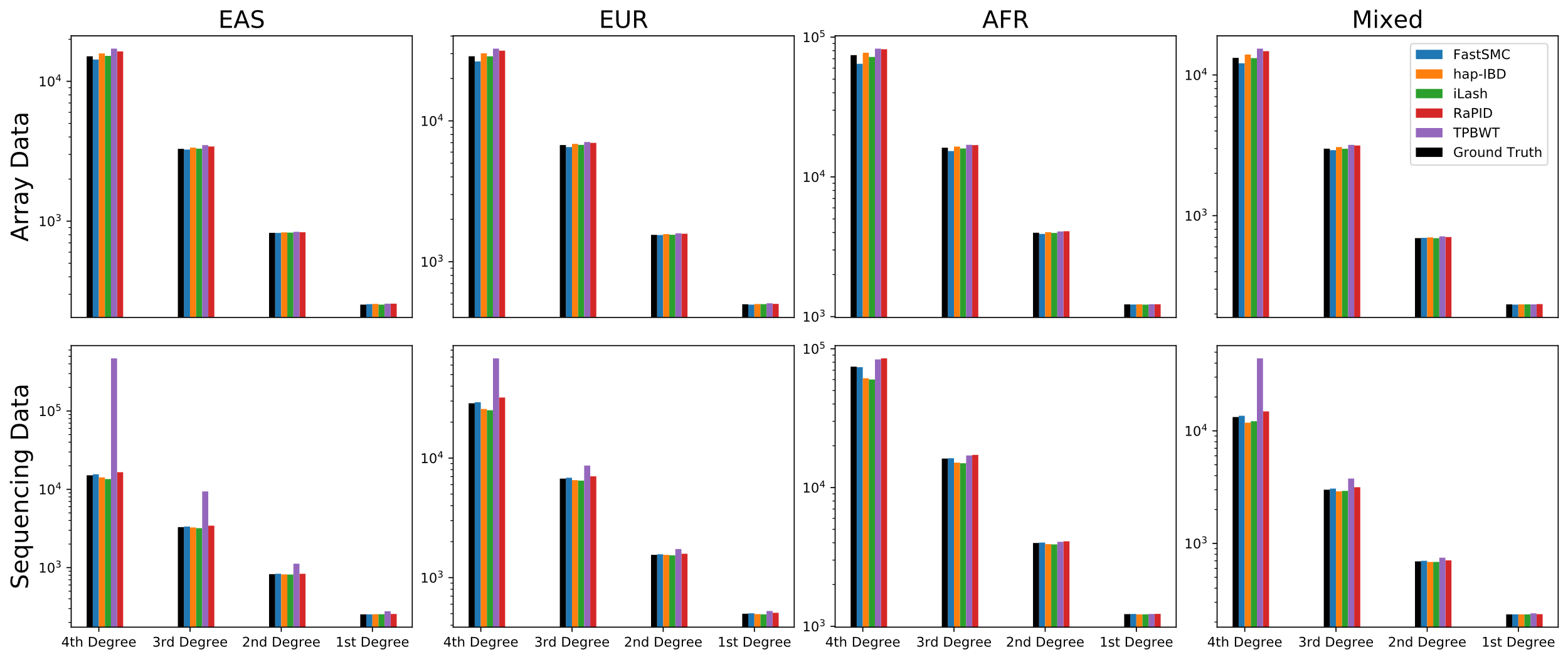


Supplementary Figure S29. Relatedness detection results in array and sequencing data without genotyping error rate on four population panels. The y-axises are number of individual pairs were found in each category. The ground truths were displayed (black bars) for reference purpose.


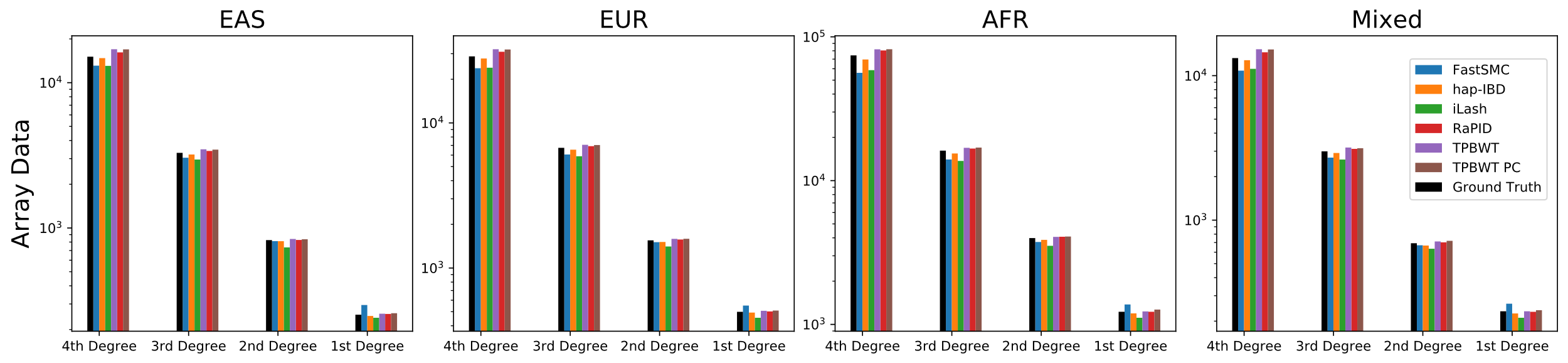


Supplementary Figure S30. Relatedness detection results in array data 0.1% genotyping error rate and phasing errors (0.31% for AFR, 0.45% for EAS, 0.38% for EUR, and 0.61% for the mixed) on four population panels. The y-axises are number of individual pairs were found in each category. “TPBWT PC” denotes the result of TPBWT with phasing error correction enabled. The ground truths were displayed (black bars) for reference purpose.


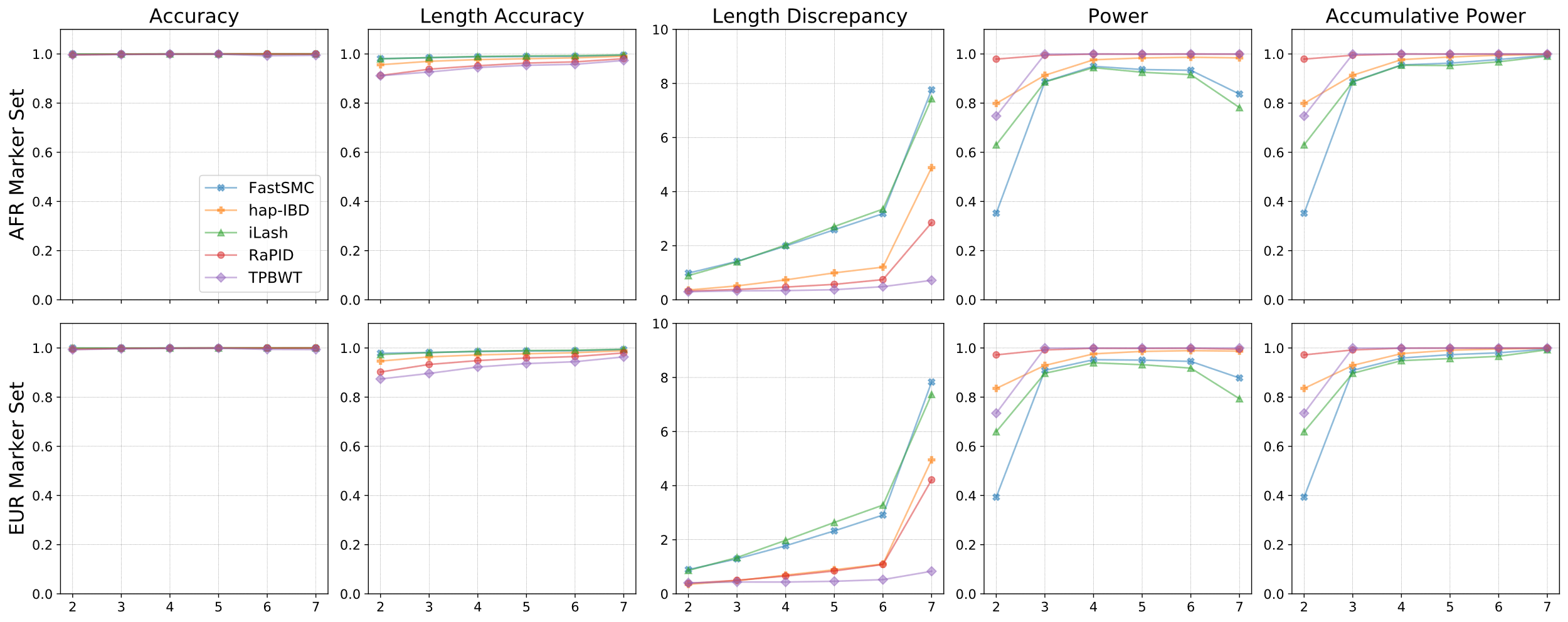


Supplementary Figure S31. Benchmarking results of different IBD detection tools in AFR array data using AFR and EUR marker set with a genotyping error rate of 0.1%. The length discrepancy is measured by cM, other measures are based on percentage.


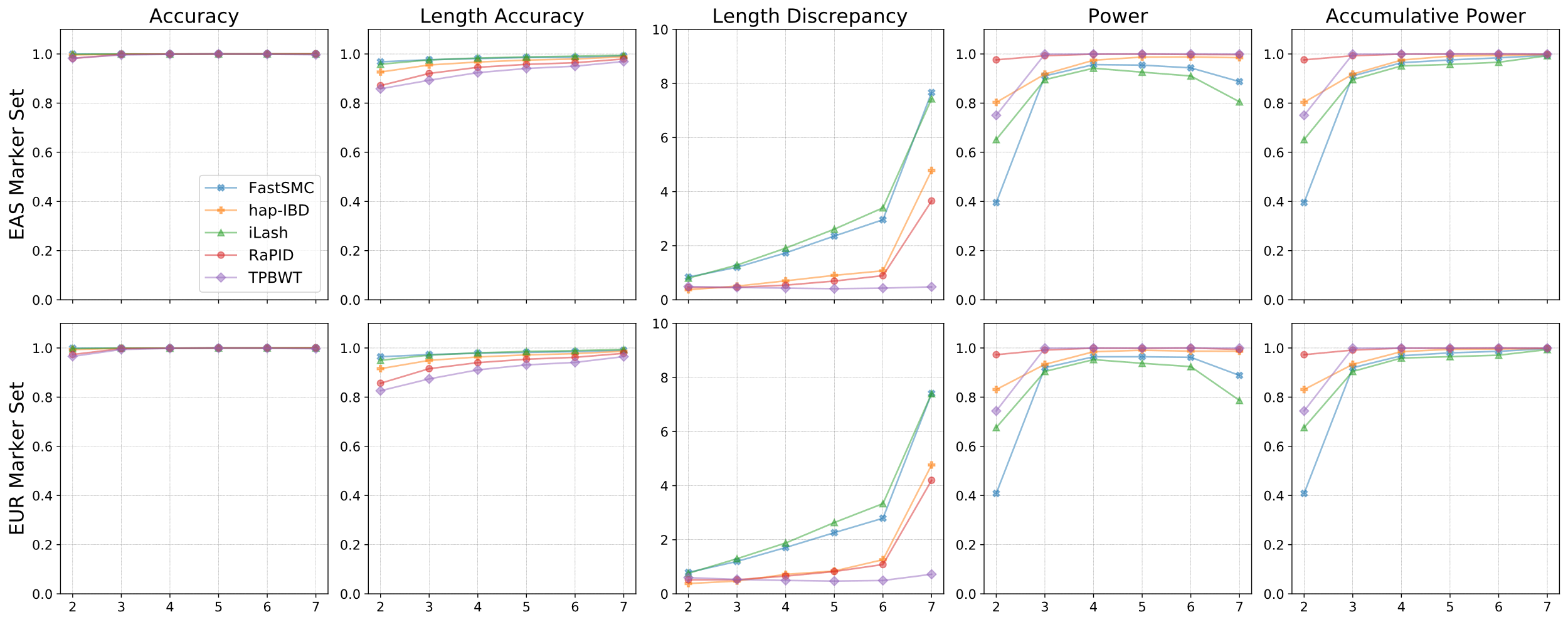


Supplementary Figure S32. Benchmarking results of different IBD detection tools in EAS array data using EAS and EUR marker set with a genotyping error rate of 0.1%. The length discrepancy is measured by cM, other measures are based on percentage.

|  | Parameters | Command Line |
| --- | --- | --- |
| FastSMC | --min_m 2 | bcftools convert ge.seq.e0.000125.vcf --hapsample ge.seq.e0.000125  ./FastSMC_exe --inFileRoot ge.seq.e0.000125\  --outFileRoot F_ge.seq.e0.000125_2\  --decodingQuantFile decodingQuantities.gz\  --mode array\  --min_m 2\  --segmentLength\  --hashing\  --perPairPosteriorMeans\  --perPairMAP\  --noConditionalAgeEstimates |
| hap-IBD | min-output=2 | java -Xmx50g -jar hap-ibd.jar gt=ge.seq.e0.000125.vcf out=H_ge.seq.e0.000125_2cM map=ge.seq.e0.0005.vcf.pMap min-output=2 |
| iLash | iLa_ge.seq.e0.000125_2.config:  ped ge.seq.e0.000125.ped  map ge.seq.e0.000125.map  output I_ge.seq.e0.000125_2.match  slice_size 350  step_size 350  perm_count 20  shingle_size 15  shingle_overlap 0  bucket_count 5  max_thread 20  match_threshold 0.99  interest_threshold 0.70  max_error 0  min_length 2  auto_slice 1  cm_overlap 1  minhash_threshold 55 | ./IBD iLa_ge.seq.e0.000125_2.config |
| RaPID | w=30  //30 for sequencing 3 for array  r=10  s=2  d=2 | ./RaPID_v.1.7 -i ge.seq.e0.000125.vcf.gz -o ge.seq.e0.000125.vcf_2w_30 -w 30 -r 10 -s 2 -d 2 -g ge.seq.e0.0005.vcf.rMap |
| TPBWT | use_phase_correction=False  L_f=2 | runTPBWT.py:  import phasedibd as ibd  haplotypes = ibd.VcfHaplotypeAlignment(path+sys.argv[2], path+sys.argv[3])  tpbwt = ibd.TPBWTAnalysis()  ibd_results = tpbwt.compute_ibd(haplotypes,L_f=float(sys.argv[1]),use_phase_correction=False,segments_out_path=path+"T_"+sys.argv[2]+"_"+sys.argv[1]+"cM.csv")  python3 runTPBWT.py 2 ge.seq.e0.000125.vcf ge.seq.e0.0005.vcf.pMap |
| Hapmap II combined map |  | ftp://ftp.ncbi.nlm.nih.gov/hapmap/recombination/2011-01_phaseII_B37/genetic_map_HapMapII_GRCh37.tar.gz |
| Data simulation |  | stdpopsim HomSap -c chr20 -o output_file -g HapMapII_GRCh37 -d OutOfAfrica_2T12 0 500000 |
| Filtering data |  | bcftools view -q 0.1:minor 500k_hapmap_chr20_p2.vcf.gz |

Supplementary Table S1. Parameters and command lines for benchmarking different IBD detection tools.

| Rel. Accuracy | [2,3) | [3,4) | [4,5) | [5,7.5) | [7.5,10) | [10,12.5) | [12.5,15) | [15, ∞) |
| --- | --- | --- | --- | --- | --- | --- | --- | --- |
| FastSMC | 0.0086 | 0.0011 | 0.0058 | 0.0018 | 0.0060 | 0.0000 | 0.0000 | 0.0000 |
| hapIBD | 0.0196 | 0.0016 | 0.0006 | 0.0028 | 0.0001 | 0.0000 | 0.0000 | 0.0024 |
| iLash | 0.0151 | 0.0028 | 0.0016 | 0.0037 | 0.0000 | 0.0169 | 0.0000 | 0.0000 |
| RaPID | 0.0278 | 0.0103 | 0.0028 | 0.0000 | 0.0001 | 0.0000 | 0.0000 | 0.0012 |
| TPBWT NPC | 0.0127 | 0.0149 | 0.0107 | 0.0001 | 0.0018 | 0.0000 | 0.0000 | 0.0006 |
| TPBWT PC | 0.0127 | 0.0149 | 0.0107 | 0.0001 | 0.0018 | 0.0000 | 0.0000 | 0.0006 |

Supplementary Table S2. Effect of phasing error on the absolute delta Accuracy of IBD detection tools using relaxed IBD segment cut-off. “TPBWT NPC” represents runs without enabling phasing error correction, and “TPBWT PC” represents runs with phasing error correction enabled. As observed the effect of phasing error is negligible in our experiment. However, enabling TPBWT phasing error correction had reduced the accuracy, since there is possibility of overcorrection by wrongfully extending/merging adjacent matches.

| Rel. Len. Acc | [2,3) | [3,4) | [4,5) | [5,7.5) | [7.5,10) | [10,12.5) | [12.5,15) | [15, ∞) |
| --- | --- | --- | --- | --- | --- | --- | --- | --- |
| FastSMC | 0.0179 | 0.0096 | 0.0119 | 0.0046 | 0.0033 | 0.0008 | 0.0009 | 0.0001 |
| hapIBD | 0.0307 | 0.0134 | 0.0082 | 0.0070 | 0.0019 | 0.0002 | 0.0011 | 0.0018 |
| iLash | 0.0240 | 0.0120 | 0.0088 | 0.0066 | 0.0021 | 0.0174 | 0.0007 | 0.0000 |
| RaPID | 0.0293 | 0.0162 | 0.0091 | 0.0058 | 0.0008 | 0.0008 | 0.0004 | 0.0015 |
| TPBWT NPC | 0.0141 | 0.0176 | 0.0138 | 0.0056 | 0.0038 | 0.0020 | 0.0006 | 0.0015 |
| TPBWT PC | 0.0141 | 0.0176 | 0.0138 | 0.0056 | 0.0038 | 0.0020 | 0.0006 | 0.0015 |

Supplementary Table S3. Effect of phasing error on the absolute delta Length Accuracy of IBD detection tools using relaxed IBD segment cut-off. “TPBWT NPC” represents runs without enabling phasing error correction, and “TPBWT PC” represents runs with phasing error correction enabled. As observed the effect of phasing error is negligible in our experiment. However, enabling TPBWT phasing error correction had reduced the accuracy, since there is possibility of overcorrection by wrongfully extending/merging adjacent matches.

| Rel. Len. Disc. | [2,3) | [3,4) | [4,5) | [5,7.5) | [7.5,10) | [10,12.5) | [12.5,15) | [15, ∞) |
| --- | --- | --- | --- | --- | --- | --- | --- | --- |
| FastSMC | 0.0312 | 0.0634 | 0.0508 | 0.0898 | 0.2183 | 0.1199 | 1.2482 | 0.2060 |
| hapIBD | 0.0992 | 0.0814 | 0.1622 | 0.2662 | 0.2192 | 0.1424 | 0.2716 | 1.0283 |
| iLash | 0.0489 | 0.0817 | 0.0485 | 0.0215 | 0.1638 | 0.6426 | 0.2979 | 0.8880 |
| RaPID | 0.0757 | 0.1277 | 0.1935 | 0.2789 | 0.6141 | 0.8268 | 0.0362 | 0.2054 |
| TPBWT NPC | 0.0251 | 0.0578 | 0.1227 | 0.1958 | 0.6640 | 1.1012 | 1.8560 | 3.6484 |
| TPBWT PC | 0.0251 | 0.0578 | 0.1227 | 0.1958 | 0.6640 | 1.1012 | 1.8560 | 3.6484 |

Supplementary Table S4. Effect of phasing error on the absolute delta Length Discrepancy of IBD detection tools using relaxed IBD segment cut-off. “TPBWT NPC” represents runs without enabling phasing error correction, and “TPBWT PC” represents runs with phasing error correction enabled. As observed the effect of phasing error is negligible in our experiment. However, enabling TPBWT phasing error correction had reduced the accuracy, since there is possibility of overcorrection by wrongfully extending/merging adjacent matches.

| Rel. Power | [2,3) | [3,4) | [4,5) | [5,7.5) | [7.5,10) | [10,12.5) | [12.5,15) | [15, ∞) |
| --- | --- | --- | --- | --- | --- | --- | --- | --- |
| FastSMC | 0.0058 | 0.0176 | 0.0074 | 0.0239 | 0.0286 | 0.0537 | 0.0236 | 0.0145 |
| hapIBD | 0.0269 | 0.0194 | 0.0113 | 0.0215 | 0.0342 | 0.0553 | 0.0326 | 0.0509 |
| iLash | 0.0125 | 0.0187 | 0.0186 | 0.0215 | 0.0290 | 0.0539 | 0.0233 | 0.0095 |
| RaPID | 0.0505 | 0.0179 | 0.0135 | 0.0202 | 0.0274 | 0.0313 | 0.0362 | 0.0385 |
| TPBWT NPC | 0.0458 | 0.0147 | 0.0127 | 0.0147 | 0.0162 | 0.0229 | 0.0139 | 0.0414 |
| TPBWT PC | 0.0458 | 0.0147 | 0.0127 | 0.0147 | 0.0162 | 0.0229 | 0.0139 | 0.0414 |

Supplementary Table S5. Effect of phasing error on the absolute delta Power of IBD detection tools using relaxed IBD segment cut-off. “TPBWT NPC” represents runs without enabling phasing error correction, and “TPBWT PC” represents runs with phasing error correction enabled. As observed the effect of phasing error is negligible in our experiment.

| Rel. Acc. Power | [2,3) | [3,4) | [4,5) | [5,7.5) | [7.5,10) | [10,12.5) | [12.5,15) | [15, ∞) |
| --- | --- | --- | --- | --- | --- | --- | --- | --- |
| FastSMC | 0.0071 | 0.0175 | 0.0069 | 0.0258 | 0.0263 | 0.0379 | 0.0019 | 0.0082 |
| hapIBD | 0.0257 | 0.0191 | 0.0110 | 0.0150 | 0.0199 | 0.0185 | 0.0098 | 0.0152 |
| iLash | 0.0132 | 0.0185 | 0.0197 | 0.0228 | 0.0187 | 0.0264 | 0.0007 | 0.0091 |
| RaPID | 0.0474 | 0.0167 | 0.0096 | 0.0099 | 0.0070 | 0.0023 | 0.0076 | 0.0027 |
| TPBWT NPC | 0.0434 | 0.0137 | 0.0105 | 0.0077 | 0.0037 | 0.0042 | 0.0001 | 0.0016 |
| TPBWT PC | 0.0434 | 0.0137 | 0.0105 | 0.0077 | 0.0037 | 0.0042 | 0.0001 | 0.0016 |

Supplementary Table S6. Effect of phasing error on the absolute delta Accumulated Power of IBD detection tools using relaxed IBD segment cut-off. “TPBWT NPC” represents runs without enabling phasing error correction, and “TPBWT PC” represents runs with phasing error correction enabled. As observed the effect of phasing error is negligible in our experiment.

| Rst. Power | [2,3) | [3,4) | [4,5) | [5,7.5) | [7.5,10) | [10,12.5) | [12.5,15) | [15, ∞) |
| --- | --- | --- | --- | --- | --- | --- | --- | --- |
| FastSMC | 0.0058 | 0.0195 | 0.0042 | 0.0136 | 0.0233 | 0.0154 | 0.0322 | 0.0162 |
| hapIBD | 0.0269 | 0.0314 | 0.0285 | 0.0183 | 0.0615 | 0.1124 | 0.0079 | 0.0516 |
| iLash | 0.0125 | 0.0188 | 0.0163 | 0.0235 | 0.0126 | 0.0102 | 0.0322 | 0.0051 |
| RaPID | 0.0505 | 0.0445 | 0.0528 | 0.0514 | 0.0812 | 0.0988 | 0.1029 | 0.0903 |
| TPBWT NPC | 0.0458 | 0.0413 | 0.0471 | 0.0428 | 0.0504 | 0.0733 | 0.0494 | 0.0902 |
| TPBWT PC | 0.0458 | 0.0413 | 0.0471 | 0.0428 | 0.0504 | 0.0733 | 0.0494 | 0.0902 |

Supplementary Table S7. Effect of phasing error on the absolute delta Power of IBD detection tools using restricted IBD segment cut-off. “TPBWT NPC” represents runs without enabling phasing error correction, and “TPBWT PC” represents runs with phasing error correction enabled. As observed the effect of phasing error is negligible in our experiment.

| CPU Time | | Sample Size (K) | | | | | |
| --- | --- | --- | --- | --- | --- | --- | --- |
| (second) | | 15 | 31 | 62 | 125 | 250 | 500 |
| 2cM Run | hapIBD | 597 | 1,447 | 4,352 | 15,400 | 61,239 | 260,976 |
|  | iLash | 13,187 | 51,711 | 232,720 | na | na | na |
|  | TPBWT | 740 | 2,738 | 10,690 | 42,715 | na | na |
| 3cM Run | hapIBD | 568 | 1,365 | 3,916 | 14,813 | 55,265 | 246,938 |
|  | iLash | 4,205 | 16,881 | 59,838 | 295,056 | na | na |
|  | TPBWT | 402 | 1,465 | 5,420 | 24,134 | na | na |
| 5cM Run | hapIBD | 555 | 1,340 | 3,846 | 13,356 | 53,396 | 227,153 |
|  | iLash | 1,482 | 4,907 | 18,557 | 66,363 | 272,303 | na |
|  | TPBWT | 278 | 863 | 3,321 | 13,011 | na | na |
| 7cM Run | hapIBD | 593 | 1,335 | 3,794 | 13,310 | 53,600 | 223,048 |
|  | iLash | 612 | 1,972 | 6,418 | 24,417 | 93,262 | 426,768 |
|  | TPBWT | 242 | 845 | 3,036 | 14,838 | na | na |

Supplementary Table S8. CPU time results of simulated large data set. na entry means the tool could not finish the task due to limited resources.

| Wall Clock Time | | Sample Size (K) | | | | | |
| --- | --- | --- | --- | --- | --- | --- | --- |
| (second) | | 15 | 31 | 62 | 125 | 250 | 500 |
| 2cM Run | hapIBD | 28 | 64 | 168 | 503 | 1,666 | 6,762 |
|  | iLash | 1,070 | 4,296 | 42,259 | na | na | na |
|  | TPBWT | 740 | 2,738 | 10,690 | 42,715 | na | na |
| 3cM Run | hapIBD | 21 | 45 | 126 | 424 | 1,488 | 6,296 |
|  | iLash | 313 | 1,133 | 4,465 | 71,698 | na | na |
|  | TPBWT | 402 | 1,465 | 5,420 | 24,134 | na | na |
| 5cM Run | hapIBD | 20 | 47 | 126 | 394 | 1,416 | 5,805 |
|  | iLash | 97 | 323 | 1,176 | 4,558 | 27,817 | na |
|  | TPBWT | 278 | 863 | 3,321 | 13,011 | na | na |
| 7cM Run | hapIBD | 22 | 45 | 124 | 390 | 1,409 | 5,970 |
|  | iLash | 47 | 175 | 665 | 2,414 | 9,832 | 83,780 |
|  | TPBWT | 242 | 845 | 3,036 | 14,838 | na | na |

Supplementary Table S9. Wall clock time results of simulated large data set. na entry means the tool could not finish the task due to limited resources.

| Memory | | Sample Size (K) | | | | | |
| --- | --- | --- | --- | --- | --- | --- | --- |
| Consumption (MB) | | 15 | 31 | 62 | 125 | 250 | 500 |
| 2cM Run | hapIBD | 2 | 7,418 | 13,542 | 27,507 | 46,150 | 103,466 |
|  | iLash | 18,684 | 65,992 | 266,363 | na | na | na |
|  | TPBWT | 7,698 | 30,593 | 122,159 | 488,396 | na | na |
| 3cM Run | hapIBD | 2 | 24,726 | 56,700 | 129,360 | 179,328 | 200,135 |
|  | iLash | 7,680 | 22,802 | 75,226 | 285,415 | na | na |
|  | TPBWT | 7,698 | 30,593 | 122,159 | 488,396 | na | na |
| 5cM Run | hapIBD | 2 | 18,153 | 59,011 | 115,340 | 88,849 | 177,534 |
|  | iLash | 3,405 | 9,829 | 27,218 | 83,688 | 302,312 | na |
|  | TPBWT | 7,698 | 30,593 | 122,159 | 488,396 | na | na |
| 7cM Run | hapIBD | 2 | 21,134 | 42,615 | 115,236 | 116,580 | 216,078 |
|  | iLash | 2,260 | 6,370 | 16,425 | 43,086 | 124,207 | 407,224 |
|  | TPBWT | 7,698 | 30,593 | 122,159 | 488,396 | na | na |

Supplementary Table S10. Memory consumption results of simulated large data set. na entry means the tool could not finish the task due to limited resources.

|  | 1 | 2 | 4 | 8 | 15 | 16 | 20 | 25 | 31 |
| --- | --- | --- | --- | --- | --- | --- | --- | --- | --- |
| Memory | 2,354,024 | 2,478,084 | 2,837,276 | 4,302,708 | 9,452,232 | 9,742,996 | 14,090,264 | 21,131,916 | 29,413,480 |
| Time | 67 | 201 | 626 | 2,157 | 8,342 | 8,736 | 13,154 | 20,978 | 34,398 |

Supplementary Table S11. FastSMC runtime and memory result on a 6 core 3.5 GHz CPU and 32 GB memory PC. The regression formula used to estimate memory consumption was y=26026x^2^+82909x+2E+06 with R^2^=0.9989. The predicted memory consumption for UK BioBank whole chromosome 1 was 6.50TB. The regression formula used to estimate runtime was y=37.574x^2^ - 80.999x + 294.52 with R² = 0.9982, at the end matched to the server 3.0 GHz clock speed, the final predicted run time was 126 days.

|  | Sample Size (K) | | | | | |
| --- | --- | --- | --- | --- | --- | --- |
|  | 15 | 31 | 62 | 125 | 250 | 500 |
| hap-IBD | 1.76 MB | 7.49 GB | 8.91 GB | 16.78 GB | 45.18 GB | 105.11 GB |
| iLash | 3.20 GB | 9.54 GB | 23.11 GB | 59.14 GB | 178.00 GB | na |
| RaPID | 456.64 MB | 1.03 GB | 1.53 GB | 2.42 GB | 3.93 GB | 6.45 GB |
| TPBWT^*^ | 7.70 GB | 30.60 GB | 122.16 GB | 488.40 GB | na | na |

Supplementary Table S12. Memory Consumptions based on UK BioBank Chromosome 1 with IBD 3cM length cut-offs by increasing sample size. The TPBWT’s results were based on the simulated panels. na entry means the tool cannot finish the task due to limited resources.

|  | Sample Size (K) | | | | | |
| --- | --- | --- | --- | --- | --- | --- |
|  | 15 | 31 | 62 | 125 | 250 | 500 |
| hap-IBD | 1.77 MB | 6.77 GB | 13.95 GB | 15.80 GB | 27.99 GB | 112.77 GB |
| iLash | 1.61 GB | 4.87 GB | 12.95 GB | 32.07 GB | 78.76 GB | 200.29 GB |
| RaPID | 386.13 MB | 718.72 MB | 1.44 GB | 2.42 GB | 3.91 GB | 6.34 GB |
| TPBWT^*^ | 7.70 GB | 30.60 GB | 122.16 GB | 488.40 GB | na | na |

Supplementary Table S13. Memory Consumptions based on UK BioBank Chromosome 1 with IBD 5cM length cut-offs by increasing sample size. The TPBWT’s results were based on the simulated panels. na entry means the tool cannot finish the task due to limited resources.
