# Supplementary material for: Open-source benchmarking of IBD segment detection methods for biobank-scale cohorts
Source: Gigascience. 2022 Dec 6;11:giac111. doi: 10.1093/gigascience/giac111 (PMC9724555; doi:10.1093/gigascience/giac111)
Supplement: giac111_Response_to_Reviewer_Comments_Revision_1 [file giac111_response_to_reviewer_comments_revision_1.pdf]

Author's Response To Reviewer Comments

Close

Please see R2_IBD_Benchmark_response.pdf for detailed response.

Close
